# T160-phosphorylated CDK2 defines threshold for HGF-dependent proliferation in primary hepatocytes

**DOI:** 10.15252/msb.20156032

**Published:** 2015-03-14

**Authors:** Stephanie Mueller, Jérémy Huard, Katharina Waldow, Xiaoyun Huang, Lorenza A D'Alessandro, Sebastian Bohl, Kathleen Börner, Dirk Grimm, Steffen Klamt, Ursula Klingmüller, Marcel Schilling

**Affiliations:** 1 Division Systems Biology of Signal Transduction, German Cancer Research Center (DKFZ)Heidelberg, Germany; 2 Analysis and Redesign of Biological Networks, Max Planck Institute for Dynamics of Complex Technical SystemsMagdeburg, Germany; 3 Translational Lung Research Center (TLRC), Member of the German Center for Lung Research (DZL)Heidelberg, Germany; 4 Centre for Infectious Diseases, Virology, Heidelberg University Hospital, Cluster of Excellence CellNetworksHeidelberg, Germany; 5 German Center for Infection Research (DZIF), Partner Site HeidelbergHeidelberg, Germany

**Keywords:** G1/S transition, hepatocyte proliferation, HGF, mathematical model, threshold

## Abstract

Liver regeneration is a tightly controlled process mainly achieved by proliferation of usually quiescent hepatocytes. The specific molecular mechanisms ensuring cell division only in response to proliferative signals such as hepatocyte growth factor (HGF) are not fully understood. Here, we combined quantitative time-resolved analysis of primary mouse hepatocyte proliferation at the single cell and at the population level with mathematical modeling. We showed that numerous G1/S transition components are activated upon hepatocyte isolation whereas DNA replication only occurs upon additional HGF stimulation. In response to HGF, Cyclin:CDK complex formation was increased, p21 rather than p27 was regulated, and Rb expression was enhanced. Quantification of protein levels at the restriction point showed an excess of CDK2 over CDK4 and limiting amounts of the transcription factor E2F-1. Analysis with our mathematical model revealed that T160 phosphorylation of CDK2 correlated best with growth factor-dependent proliferation, which we validated experimentally on both the population and the single cell level. In conclusion, we identified CDK2 phosphorylation as a gate-keeping mechanism to maintain hepatocyte quiescence in the absence of HGF.

## Introduction

The liver possesses the ability to restore its mass upon intoxication or upon surgically removing up to two-thirds of the organ. During the regenerative process, usually quiescent hepatocytes proliferate to compensate for the lost tissue (Michalopoulos, 2007). During the regeneration phase, the liver receives a variety of signals that coordinate its timely progression. Hepatocyte growth factor (HGF) is a major driver of hepatocyte proliferation during this *in vivo* process and also a direct mitogen to these cells in culture (Runge *et al*, 1999; Nakamura *et al*, 2011). Animals harboring a deletion of the HGF receptor c-Met show strongly impaired regeneration upon two-thirds partial resection of the liver (partial hepatectomy, PHx), and deletion of c-Met cannot be compensated by other ligands or receptors (Borowiak *et al*, 2004). Thus, HGF appears to be an irreplaceable factor orchestrating liver regeneration (Michalopoulos, 2007).

To enable proliferation, adult hepatocytes exit their quiescent state and re-enter the cell cycle that is divided into the four phases G1, S, G2, and M (Malumbres & Barbacid, 2001; Morgan, 2007). A major hallmark of the G1 phase is that cells integrate the signals they receive from their environment (Coller, 2007). Based on this information, cells may commit to proliferation by crossing the restriction point during the transition from G1 into S phase or withdraw from proliferation (Pardee, 1974; Malumbres & Barbacid, 2001; Massague, 2004; Coller, 2007). Due to the liver's central role in metabolism and detoxification, hepatocytes are regularly subjected to a variety of signals and stress cues of metabolic origin, some of which can have undesired, pro-proliferative properties potentially triggering abnormal proliferation (Rahman *et al*, 2013). Thus, control mechanisms within the G1/S transition must exist that enable hepatocytes to distinguish between true proliferative signals such as HGF released during the liver regeneration process and other, non-specific stress cues, thus permitting proliferation only in the presence of the correct signal.

Orderly progression through the cell cycle is orchestrated by the sequential activation of Cyclin-dependent kinases (CDK). These serine–threonine kinases form complexes with a regulatory Cyclin subunit to phosphorylate and thereby control numerous substrates (Morgan, 1997). However, Cyclin binding is insufficient to trigger full kinase activity. In the case of CDK2, for example, phosphorylation at T160 within the T loop of the kinase domain by the nuclear CDK-activating kinase (CAK) is essential for full activation, as unphosphorylated T160 blocks ATP binding (De Bondt *et al*, 1993; Morgan, 1997; Malumbres & Barbacid, 2005). During the G1 phase, the complexes Cyclin D1:CDK4 and Cyclin E:CDK2 are successively activated leading to the stepwise phosphorylation, and thus, inhibition of the retinoblastoma protein (Rb) (Malumbres & Barbacid, 2001, 2005; Obaya & Sedivy, 2002). This process results in the release and autocatalytic activation of the transcription factor E2F-1 and creates a bistable switch at the single cell level, permitting cells to cross the restriction point (Yao *et al*, 2008).

Growth factor- and specifically HGF-induced signaling is known to modulate the transition from G1 to S phase at multiple levels. Firstly, HGF induces the expression of various genes. For example, Cyclin D1 expression is induced following activation of the mitogen-activated protein kinase (MAPK) signaling cascade in response to HGF stimulation (Wilkinson & Millar, 2000). Furthermore, HGF-dependent signaling influences the stability of G1 components by regulating protein degradation. For instance, HGF-dependent inhibition of GSK3β activity allows accumulation of Cyclin D1, and thus, formation of Cyclin D1:CDK4 complexes (Liang & Slingerland, 2003). Lastly, HGF-dependent phosphorylation of the CIP/KIP family of CDK inhibitors, that is, p21 and p27, by ERK or Akt critically influences their binding to and interaction with CDKs. Binding of these proteins on the one hand enhances complex formation between Cyclin and CDK. On the other hand, however, depending on the phosphorylation state, p21 and p27 can also block the catalytic kinase domain of CDKs, thereby inactivating the complex. Additionally, binding of p21 and p27 enables nuclear localization of Cyclin:CDK complexes, which is a prerequisite for nuclear T loop phosphorylation and therefore full activation of CDK, as well as for bringing the kinase in close proximity to its nuclear targets (Morgan, 1997; Sherr & Roberts, 1999; Coqueret, 2003; Besson *et al*, 2008). Because of these activating and inhibiting functions of p21 and p27, CDK regulation is highly nonlinear.

Evidently, multiple components contribute to the regulation of cell cycle progression in hepatocytes. Due to the complexity of these interactions, mechanistic mathematical models provide useful tools to gain insights into the interlaced regulatory network. Previously, several mathematical models analyzing the progression of cells through G1 and S phase have been reported (Qu *et al*, 2003; Novak & Tyson, 2004; Swat *et al*, 2004; Haberichter *et al*, 2007; Chauhan *et al*, 2008; Yao *et al*, 2008; Alfieri *et al*, 2009; Csikasz-Nagy, 2009; Conradie *et al*, 2010). Since these models have not been calibrated to a specific cellular context or cell type, they reveal general properties of cell cycle control and thus describe the behavior of a “generic” proliferating cell (Csikasz-Nagy, 2009). The model parameters were often extracted from literature and originated from various biological systems like yeast or immortalized cell lines that harbor alterations in cell cycle control. Since cell type-specific characteristics such as the relative abundance of pathway proteins and kinetic rate constants critically determine the behavior of a biological system, such generic mathematical models may have limited predictive power when investigating the behavior of a specific cell type (Aldridge *et al*, 2006). Most importantly, primary and tumor cells display major differences especially with respect to cell cycle control as malignant cells often lack G1/S checkpoint control (Berthet & Kaldis, 2007).

While many mathematical models were calibrated based on data obtained by the analysis of cell populations, recently increasing efforts have been directed toward explaining single cell level behaviors. Experimental methods for single cell analysis include live cell microscopy in combination with fluorescent reporters. Such combinations, for example, enabled the characterization of the coupling between cell cycle and circadian clock in mouse fibroblasts (Bieler *et al*, 2014) and the mathematical modeling of extrinsic apoptosis in single cells (Albeck *et al*, 2008). Recently, a live cell sensor for CDK2 activity was introduced, demonstrating that CDK2 activity constitutes a threshold that controls the proliferation–quiescence decision (Spencer *et al*, 2013). Furthermore, the development of the Fluorescence ubiquitination-based cell cycle indicator (Fucci) reporter system facilitates time-resolved monitoring of cell cycle phases in single cells (Sakaue-Sawano *et al*, 2008). The combination of the Fucci reporter with live cell microscopy was utilized for the measurement and mathematical modeling of the duration of cell cycle phases in proliferating lymphocytes (Dowling *et al*, 2014).

To characterize HGF-driven progression through the G1 phase in primary mouse hepatocytes, we established a cell type-specific mathematical model of the HGF-induced input signals and the G1/S transition components based on ordinary differential equations (ODE). It was calibrated using quantitative time-resolved measurements of protein species and DNA content in primary mouse hepatocytes. To determine the mechanism preventing transition from G1 to S in the absence of HGF, we performed model analyses quantifying the influence of HGF on G1/S transition components. We identified phosphorylation of CDK2 on T160 as the crucial determinant for hepatocyte proliferation. This model prediction was experimentally verified at both the population and the single cell level by demonstrating a linear relationship between phosphorylation of CDK2 at T160 and the number of primary mouse hepatocytes in S/G2/M phase. This approach enabled us to identify phosphorylation of CDK2 at T160 as a gate-keeping mechanism for hepatocyte proliferation.

## Results

### Determination of restriction point timing in primary mouse hepatocytes

**Figure 1:**
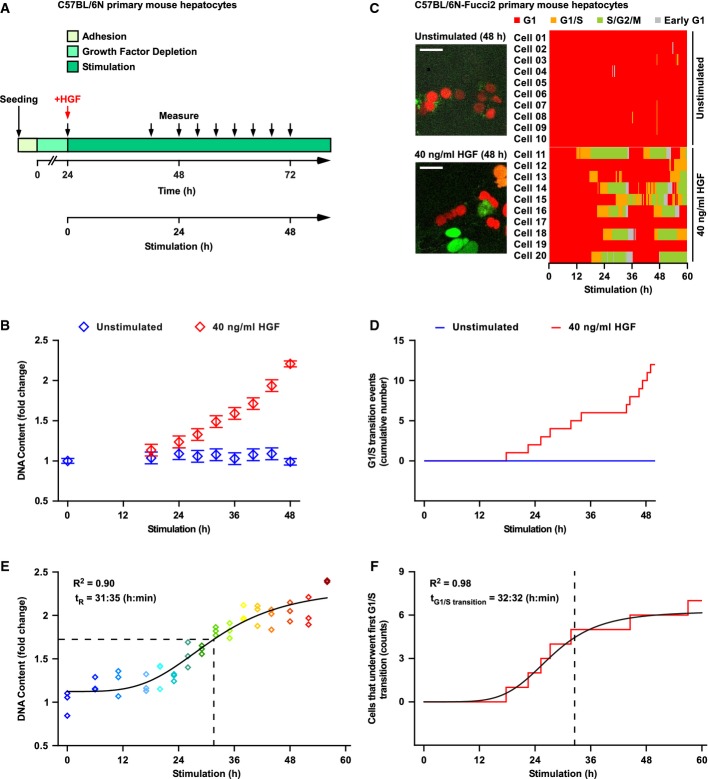
Hepatocytes require HGF for DNA synthesis and pass the restriction point after 32 h of stimulation with HGF

We established a highly standardized workflow for the isolation and *ex vivo* cultivation of primary mouse hepatocytes (Fig1A). Hepatocytes were isolated by *in situ* liver perfusion. For culturing, cells were allowed to adhere in serum-supplemented cultivation medium for 4 h, followed by growth factor depletion for 24 h under serum-free conditions. Hepatocytes were stimulated with 40 ng/ml HGF or left unstimulated. They were subsequently collected at the indicated time points for up to 48 h of stimulation, and DNA content was measured by Sybr Green staining. While unstimulated hepatocytes showed no change, the DNA content of HGF-stimulated hepatocytes doubled within 48 h (Fig1B).

Primary mouse hepatocytes were isolated by liver perfusion and allowed to attach, and growth factors were depleted for 24 h. Then, cells were stimulated with 40 ng/ml HGF or remained untreated for the entire experiment. After distinct time intervals (black arrows), cells were collected for DNA content measurement.

Primary mouse hepatocytes cultivated according to the scheme depicted in (A) were assayed for DNA content using Sybr Green I. Open diamonds represent the mean of three to 17 scaled and merged biological replicates. Error bars were estimated based on the Sybr Green I data using a linear error model.

Primary mouse hepatocytes from mice transgenic for the Fucci2 cell cycle sensors were isolated and cultivated as schematized in (A) and transduced with adeno-associated viral vectors encoding Histone2B–mCerulean to enable tracking of the cells. Live cell microscopy was performed with sampling rate of 15 min for up to 60 h, and 20 cells were tracked (Supplementary Fig S1A). The time-dependent cell cycle phases G1, G1/S, and S/G2/M and early G1 are displayed for primary mouse hepatocytes treated with 40 ng/ml HGF or left untreated. Scale bar: 50 μm.

Entries into the S/G2/M phase shown in (C) were quantified and defined as G1/S transition events. The cumulative number of G1/S transition events is displayed for both unstimulated and 40 ng/ml HGF-stimulated hepatocytes.

Primary mouse hepatocytes were stimulated with 40 ng/ml HGF 24 h after isolation or remained untreated for the entire experiment. After distinct time intervals (color coded), cells were washed three times with PBS and received stimulus-free cultivation medium supplemented with 2.5 μM PHA 665752 c-Met inhibitor. Cultivation was continued for a total time of 80 h, and cells were collected for DNA content measurement using Sybr Green I (Supplementary Fig S1B). One representative biological replicate is shown, which was performed in technical triplicates (open diamonds). Restriction point (t_R_) was determined by fitting a four-parameter Hill function to the data (solid line), and calculating the inflection point (dashed line). The experiment was performed in biological triplicates (Supplementary Table S1).

The number of cells stimulated with 40 ng/ml that underwent G1/S transition at least once was quantified from the data displayed in (C) at each time point. Cell counts are displayed in a time-resolved manner (red solid line). For visualization, a four-parameter Hill function was fitted to the data (black solid line). The average time point of G1/S transition (t_G1/S transition_) is indicated with a vertical dashed line.

Source data are available online for this figure.

Our population data show a continuous increase of DNA content, starting at 24 h after stimulation with 40 ng/ml HGF. To investigate whether the shape of this curve is caused by temporal variability of the initiation of DNA synthesis in each cell, we analyzed the cell cycle progression in individual hepatocytes in the same experimental setting. We isolated primary mouse hepatocytes from mice transgenic for the Fucci2 cell cycle sensors established by Abe *et al* (2013) (Fucci2 hepatocytes). These mice express mCherry-hCdt1 (amino acids 30–120) and mVenus-hGem (amino acids 1–110) under control of the Rosa26 promoter to distinguish cell cycle phases at the single cell level, but are otherwise of the same genetic background as the wild-type mice used for our population studies (C57BL/6N). Additionally, we transduced the Fucci2 hepatocytes with adeno-associated viral vectors encoding Histone2B–mCerulean to facilitate single cell tracking. We performed live cell microscopy of growth factor-depleted Fucci2 hepatocytes stimulated with 40 ng/ml HGF or left untreated (sampling rate of 15 min for up to 60 h) and manually tracked 20 cells (Supplementary Fig S1A). This data set allowed us to define the G1 (red), the G1/S (orange), the S/G2/M (green), and the early G1 (gray) phases of the cell cycle in a time-dependent manner. Fucci2 hepatocytes were in G1 phase after isolation. Without stimulation, hepatocytes remained in G1 throughout the observation period. A few cells entered the G1/S phase, but returned to G1 phase, indicating that the G1/S phase defined by the Fucci2 reporter does not necessarily lead to DNA replication. Stimulation with 40 ng/ml HGF induced most of the monitored hepatocytes to undergo G1/S transition. These cells entered S/G2/M phase, executed DNA replication, and performed mitosis and cytokinesis, as observed in the transmitted light channel. Immediately following mitosis, cells were in early G1 phase (Fig1C). To link these single cell results with our population data, we quantified time-dependent G1/S transition (entry into the S/G2/M phase) events in these cells. The cumulative number of G1/S transition events versus time (Fig1D) is consistent with the measured increase of the DNA content of the entire population during the observation period. This congruent behavior indicates a similar HGF-dependent proliferation of the hepatocyte population and the averaged Fucci2 hepatocytes. We observed that while most of the cells respond to 40 ng/ml HGF during the observation period, the timing of transition to S/G2/M varied between the individual cells. Therefore, we expected that analysis of the G1/S transition on the population level would reveal a sigmoidal response, rather than a step-like increase.

To determine the timing of the passage through the restriction point of the hepatocyte population, hepatocytes were subjected to pulsed stimulation with 40 ng/ml HGF (see workflow in Supplementary Fig S1B). The pulse was terminated by washing the cells and adding fresh, serum-free cultivation medium supplemented with the c-Met inhibitor PHA665752 to prevent effects triggered by residual ligand. A maximal HGF stimulation time of 56 h was tested, and the DNA content was measured as readout for cell proliferation. In line with the expectation from the single cell analysis, the DNA content of primary mouse hepatocytes responded to HGF withdrawal in a sigmoidal fashion (Fig1E). We approximated the average restriction point by fitting a four-parameter Hill function to the obtained data and calculating the inflection point of the resulting sigmoidal curve. The analysis of three biological replicates (Supplementary Table S1) showed that on average hepatocytes became insensitive to growth factor removal and thus passed the restriction point after 32 ± 2 h of HGF stimulation.

In single cells, G1/S transition occurs at a distinct time and is controlled by a specific threshold. In this case, the term threshold corresponds to a distinct value (a concentration of a specific signaling molecule) at which an event (G1/S transition) is triggered in a single cell. If in a cell population all hepatocytes underwent G1/S transition at the same time, the DNA content would increase at this time point. On the other hand, if the different hepatocytes in a population underwent G1/S transition at different times after exposure to HGF, the response would be more graded. To experimentally test this hypothesis, we quantified the time of G1/S transition in the Fucci2 hepatocytes. Figure1F shows the number of hepatocytes that underwent G1/S transition at least once, plotted in a time-resolved manner. The data could be approximated with a sigmoidal function featuring comparable slope and steepness compared to our cell population data. Our data showed that the timing of G1/S transition varies between individual hepatocytes, with an average G1/S transition time corresponding to ∽33 h. Therefore, we conclude that individual hepatocytes undergo G1/S transition at different times after HGF stimulation, converting a step-like individual decision to a sigmoidal population response.

### Analysis of G1/S phase progression and abundance of pathway proteins in primary mouse hepatocytes

**Figure 2:**
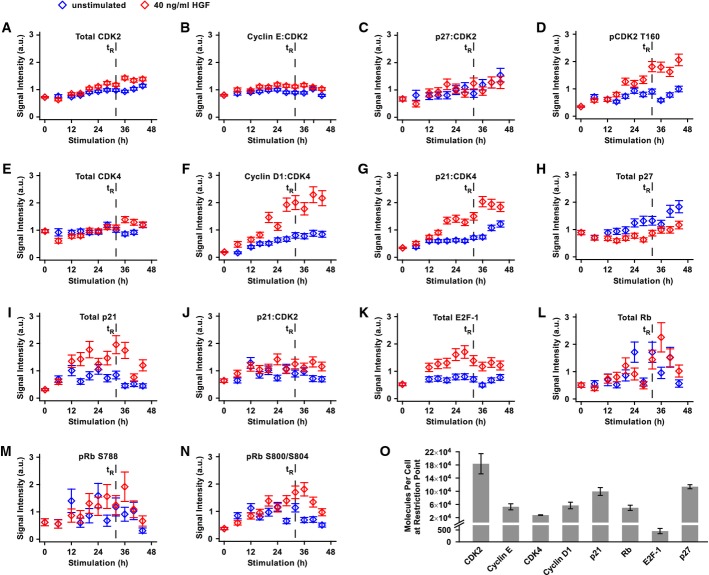
Dynamics and protein abundance of G1/S transition components in primary mouse hepatocytes

To analyze the dynamics of key pathway components contributing to G1/S phase progression in primary mouse hepatocytes, we acquired densely sampled time-resolved quantitative immunoblotting measurements of key G1/S transition components, that is, CDK2 (Fig2A), CDK4 (Fig2E), p27 (Fig2H), p21 (Fig2I), E2F-1 (Fig2K), and Rb (Fig2L). Additionally, we recorded complex formation of CDK2 with Cyclin E (Fig2B), p27 (Fig2C), and p21 (Fig2J) and the association of CDK4 with Cyclin D1 (Fig2F) and p21 (Fig2G) as well as the selected phosphoforms pCDK2 T160 (Fig2D), pRb S788 (Fig2M), and pRb S800/S804 (Fig2N). To discriminate stimulus-dependent and stimulus-independent effects, the dynamics of pathway components were monitored in primary mouse hepatocytes stimulated with 40 ng/ml HGF or in untreated, time-matched control cells. Again, the cultivation scheme depicted in Fig1A was used, applying continuous HGF stimulation. Representative immunoblots are depicted in Supplementary Fig S2, and the quantification results are shown in Fig2A–N. For these data, up to seven biological replicates were merged and the error was estimated using a linear error model (Supplementary Fig S3). By performing control experiments using blocking peptides or competing amounts of recombinant proteins (Supplementary Fig S4), we confirmed the specificity and the quality of our antibodies.

A-N Twenty-four hours after isolation, primary mouse hepatocytes were stimulated with 40 ng/ml HGF or remained untreated. Cells were lysed at indicated time points using total cell lysis buffer, and cell cycle proteins were subjected to immunoprecipitation. Data were recorded by quantitative immunoblotting. Open diamonds represent the mean of three to seven scaled and merged biological replicates. A linear error model was employed to calculate error bars (Supplementary Fig S3). The vertical dashed line corresponds to the calculated restriction point. t_R_: restriction point. a.u.: arbitrary units.

O Abundance of cell cycle proteins at the restriction point is depicted. Primary mouse hepatocytes were lysed at *t* = 56 h (32 h after stimulation with 40 ng/ml HGF, corresponding to the restriction point determined in Fig1) using total cell lysis buffer and subjected to immunoprecipitation. Prior to immunoprecipitation, a dilution series of recombinant calibrator protein was spiked into the lysates. Signals were recorded by quantitative immunoblotting (Supplementary Fig S5). Absolute protein amounts were calculated based on the standard curves depicted in Supplementary Fig S5, taking into account the molecular weights of recombinant calibrator and endogenous protein as well as the number of lysed cells. Error bars represent standard deviation of three to five biological replicates.

Source data are available online for this figure.

Subsequent to a minor initial drop, the expression of CDK4 (Fig2E) and CDK2 (Fig2A) increased slightly in stimulated hepatocytes, while they appeared rather constant in untreated hepatocytes. CDK4-bound Cyclin D1 (Fig2F) rose linearly upon HGF stimulation and reached a plateau at 30 h of HGF exposure and thus correlated with the timing of passage through the restriction point in primary hepatocytes. In addition, the dynamics of p21:CDK4 were similar to the dynamics of Cyclin D1:CDK4. Unstimulated hepatocytes showed only a minor increase in Cyclin D1:CDK4 over time. Interestingly, CDK2-containing complexes showed a rather unexpected behavior. CDK2-bound Cyclin E (Fig2B) displayed only a small increase upon HGF stimulation and CDK2-bound p21 (Fig2J) remained constant. CDK2-bound p27 (Fig2C) increased slightly over time, independent of HGF stimulation. Interestingly, while there was only a minor effect of HGF on Cyclin E binding, the activating phosphorylation of CDK2 at T160 (Fig2D) showed a pronounced HGF-specific behavior and increased strongly upon ligand stimulation. Slightly delayed compared to CDK4-bound Cyclin D1 and p21, it reached sustained levels at approximately 30 h of stimulation. The sustained phosphorylation of CDK2 at T160 could facilitate further rounds of hepatocyte replication as observed in Fig1C.

The Cip/Kip proteins p21 and p27 displayed distinctive behavior. Total p21 levels (Fig2I) showed a broad peak around 20 h, and this effect was more pronounced in HGF-stimulated than in untreated hepatocytes. On the contrary, total p27 (Fig2H) remained constant in the presence of ligand, but increased over time in its absence.

The E2F family of transcription factors is mainly inhibited by pocket proteins such as p107, p130, and Rb (Dyson, 1998; Stevaux & Dyson, 2002; Cobrinik, 2005; Dimova & Dyson, 2005). E2F-1 seems to be only required for the first round of proliferation, but not in constantly cycling cells (Kong *et al*, 2007). Because we focus here on the first round of DNA synthesis in hepatocytes, we only monitored this family member. Indeed, E2F-1 (Fig2K) was substantially up-regulated upon HGF stimulation and showed a rather broad peak after around 26 h of stimulation just before the restriction point and its levels remained elevated thereafter. Surprisingly, Rb expression (Fig2L) was not constant, but increased over time and showed a peak around 35–40 h after HGF stimulation. In accordance with the increase in total Rb protein levels, the phospho-species pRb S788 (Fig2M) followed this behavior. Interestingly, the second phosphorylation of Rb at S800/S804 (Fig2N) that is catalyzed by active CDK2 complexes showed a distinct HGF dependency at late time points.

For our further analyses, we selected total proteins or phosphorylated proteins as G1/S transition components based on the following criteria: The component is (i) regulated during the observed time frame, and (ii) the regulation is substantially different if cells are stimulated with 40 ng/ml HGF compared to the untreated condition. Additionally, we selected Cyclin D1:CDK4 as representative of the trimeric complex Cyclin D1:CDK4:p21 to avoid redundant information. Based on these criteria, we focused our further analyses on the following G1/S transition components in primary mouse hepatocytes: Cyclin E:CDK2 and Cyclin D1:CDK4 complexes, pCDK2 T160, total p21, the p21:CDK2 complex, total E2F-1, total Rb as well as the phosphorylated species pRb 788, and pRb S800/S804.

Apart from the dynamics, the relative abundance of the components contributing to the G1/S transition is critical. Therefore, the number of molecules per hepatocyte at the time of passage through the restriction point was determined by quantitative immunoblotting (Schilling *et al*, 2005). For the calculation, a calibration curve was established based on a dilution series of recombinant calibrator proteins that were added to primary mouse hepatocyte lysates prior to immunoprecipitation (Fig2O and Supplementary Fig S5). To convert these numbers into concentrations, we determined by confocal microscopy the total volume of primary mouse hepatocytes to be 12.92 ± 0.62 pl (Supplementary Fig S3D). Interestingly, primary mouse hepatocytes contained ∽7 times more CDK2 than CDK4 (Fig2O), which corresponded to a concentration of roughly 3.5 nM for CDK4, and approximately 23.6 nM for CDK2. The associated Cyclins E and D1 as well as p21, p27, and Rb showed expression levels in between these values. At the restriction point, the concentration of E2F-1 was very low, suggesting that its level might be limiting for the transition from G1 to S phase.

### Establishment of a quantitative dynamic model describing HGF-regulated G1/S phase progression in primary mouse hepatocytes

To understand the observed hepatocyte-specific cell cycle properties and to elucidate how the G1/S transition is prevented in the absence of HGF, we developed a mathematical model. The model presented here consists of two parts, a simplified input model and a quantitative dynamic model describing the activation of G1/S transition components and DNA synthesis.

**Figure 3:**
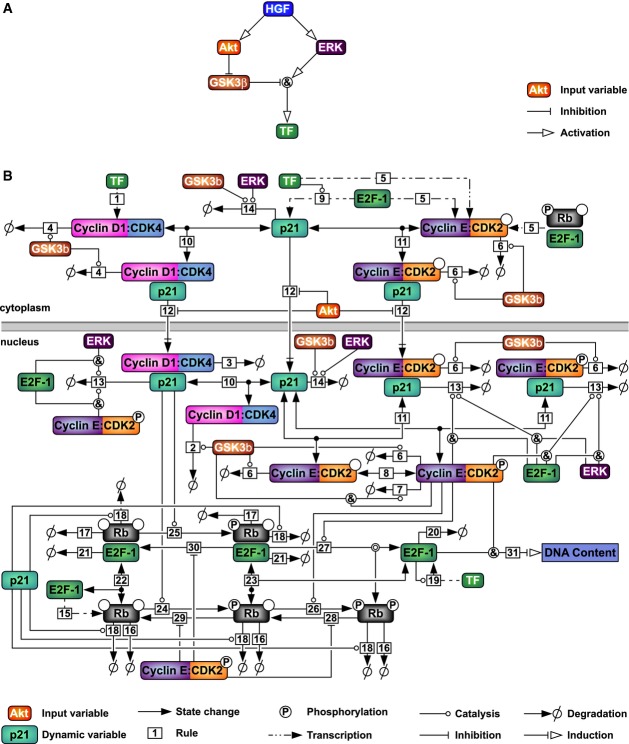
Hepatocyte-specific mathematical model of cell cycle progression

The input model (Fig3A) is modulated by the HGF concentration. HGF activates signaling networks including Akt, ERK, GSK3β, and the transcription factors (TF) AP-1, Myc, and p53. We incorporate these network components as input variables into our dynamic model and calculate their state using algebraic equations (Fig3A and Supplementary Table S4). The input variables have a value in the range of 0 (no activity) to 1 (maximum activity). The algebraic equations reflect the dependency between these variables and are based on our previously published hepatocyte-specific logical model (Huard *et al*, 2012). Additionally, we experimentally determined the basal activity of Akt and ERK as well as the dose-dependent activation of Akt in response to HGF in our cell system (Supplementary Fig S7).

The simplified input model is schematically depicted. Algebraic equations (reflecting signaling logic) are used to map HGF input to the state of key signaling species. Activating reactions are shown with arrows; inhibitory reactions are depicted with bar-ended edges. The ampersand represents a logical AND gate.

The quantitative dynamic mathematical model is schematically shown. Input variables corresponding to the network in (A) are depicted with white fonts; dynamic model variables are displayed with black fonts. Solid lines indicate kinetic reactions such as production, degradation, complex formation, and protein modifications. Nuclear import is depicted by a vertical bar followed by an arrow. Every edge is labeled with the number of the corresponding rule explained in detail in the Supplementary Information.

The variables of the simplified input model serve as inputs into our dynamic model, which is schematically depicted in Fig3B. Our model describes the regulation of the main G1/S transition components depicted in Fig3B: Cyclin D1:CDK4, Cyclin E:CDK2, p21, Rb, and E2F. To facilitate the establishment of differential equations for this large network, we employed rule-based modeling (Faeder *et al*, 2009). A detailed description of the model interactions and the equations employed is given in the Supplementary Information. Briefly, the quantitative dynamic model comprises the regulation of Cyclin D1:CDK4 complexes, the regulation of Cyclin E:CDK2 complexes, the phosphorylation of Rb, the release of E2F-1, and the link to DNA replication. The model structure is not only based on literature information, but also reflects hepatocyte-specific properties according to our experimental results. We employed the following simplifications and considered specific regulatory mechanisms in primary mouse hepatocytes.

We experimentally observed in hepatocytes that the complex of CDK2:p27 concentration does not depend on HGF stimulation and that p27 in contrast to the literature is not strongly degraded during the G1 phase (Fig2H). We therefore did not consider p27 in our model. To reduce model complexity, we did not consider individual Cyclins and CDKs, but focused on complexes only. In the case of Cyclin E:CDK2, this assumption is justified by our observation that CDK2 is present in excess compared to Cyclin E, suggesting that Cyclin E is predominantly bound to CDK2 (Fig2O). Cyclin D1:CDK4 is known to be rather unstable as dimer and requires p21 for assembly (Cheng *et al*, 1999). Because in our experimental data Cyclin D1:CDK4 and p21:CDK4 showed comparable dynamics (Fig2F and G), the species Cyclin D1:CDK4 in our model represents free Cyclin D1, free CDK4 proteins, and the complex Cyclin D1:CDK4:p21. A consequence of this assumption is that our model cannot distinguish between p21:CDK4 and Cyclin D1:CDK4. Furthermore, we assume that binding of p21 does not impair kinase activity of CDK4. CAK-mediated CDK4 phosphorylation, which is not modeled explicitly, as CAK is constitutively active (Ray *et al*, 2009), is therefore assumed to occur immediately after nuclear import, regardless of bound p21. p21 also mediates nuclear translocation of CDK2, but in contrast to CDK4, CDK2 can only be active if p21 is not bound to the complex (LaBaer *et al*, 1997), and we therefore assume that it can only be phosphorylated after release of p21. Also, we assume a sequential phosphorylation of Rb by firstly CDK4, and secondly CDK2. This is motivated by Hatakeyama *et al* (1994), who demonstrated that a combination of CDK4/6 and CDK2 kinase activities is required for full phosphorylation of Rb. This assumption is also supported by the work of Connell-Crowley *et al* (1997) who reported that phosphorylation by CDK4 alone is not sufficient to inactivate Rb. p21 expression is induced by p53 and E2F transcription factors. Similar to the auto-transcription of E2F-1 that requires Myc as co-factor (Leung *et al*, 2008), we hypothesize that p21 induction by E2F-1 requires p53. The stability of E2F was shown to depend on its binding status (Helin, 1998). Thus, we assume that the half-life of Rb increases after binding to E2F-1. Finally, to simplify the structure of our input network, our input species are assumed to be present in both cytoplasm and nucleus.

### G1/S transition regulation in unstimulated and HGF-stimulated primary mouse hepatocytes

It has been shown that stress induced by the isolation procedure and plating of the cells can activate signaling pathways including the PI3K/Akt and the Raf/MEK/ERK pathway (Fausto *et al*, 1986; Loyer *et al*, 1996), which may contribute to a growth factor-independent increase in G1/S transition components. To determine the basal activities of these pathways, we quantified the phosphorylation of Akt and ERK in unstimulated hepatocytes 24 h after adhesion (Supplementary Fig S7A and B) as well as in hepatocytes stimulated with 40 ng/ml HGF for 1 h. In addition, we inhibited the upstream kinases PI3K and MEK to determine the experimental background of our immunoblot measurements. This allowed us to calculate the two parameters αakt and αerk that represent the basal activity of Akt and ERK, respectively. Furthermore, we quantified in HGF-stimulated primary mouse hepatocytes the phosphorylation of Akt on Ser473 required for complete activation of Akt (Sarbassov *et al*, 2005), and the double phosphorylation of ERK (Thr203 and Tyr205 in murine ERK1; Thr183 and Tyr185 in murine ERK2) corresponding to active ERK (Boulton *et al*, 1991; Robbins *et al*, 1993). Additionally, we measured by a quantitative bead-based multiplex assay the levels of Akt Ser473 phosphorylation in HGF dose–response experiments (Supplementary Fig S7C).

**Figure 4:**
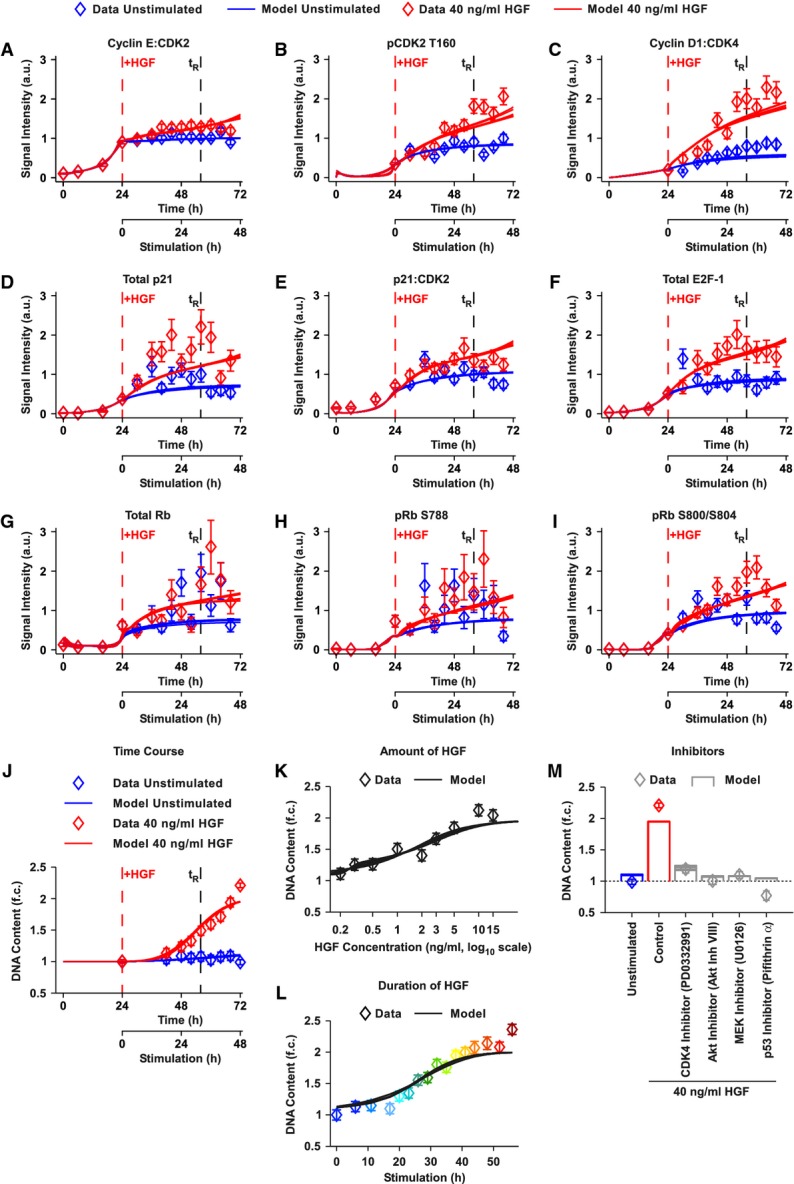
The calibrated mathematical model describes the dynamics of cell cycle proteins in both unstimulated and HGF-stimulated primary mouse hepatocytes

To assess the growth factor-independent impact on the dynamics of the G1/S transition components, we monitored their dynamics during the first 48 h following adhesion. This encompasses the 24-h growth factor depletion phase as well as the first 24 h of the stimulation period (Supplementary Fig S6). Interestingly, while the G1/S transition components were low or undetectable directly after isolation, they became significantly upregulated during the growth factor depletion phase. The combination of this data set with the data depicted in Fig2 and Supplementary Fig S2 revealed the regulation of the G1/S transition components in unstimulated as well as in HGF-stimulated primary mouse hepatocytes in our *ex vivo* setting (Fig4A–I, data points): After isolation and adhesion of the primary mouse hepatocytes, the concentration of the G1/S transition components is constantly increasing. Without external stimulation, the concentrations reach a new steady state level at around 30 h after adhesion. If the cells are additionally stimulated with 40 ng/ml HGF after 24 h of growth factor depletion, the concentrations reach an even higher steady state. Only this second increase appears to be sufficient to trigger passage through the restriction point, indicating the existence of a distinct threshold.

A-I The 10 best results of 500 parameter estimation rounds are shown (solid lines). Open diamonds represent the data shown in Fig2, supplemented with the dynamics of the cell cycle proteins between isolation and stimulation (Supplementary Fig S6). Data points represent the mean of three to nine scaled and merged biological replicates. Error bars were estimated based on the quantitative immunoblot data using a linear error model (Supplementary Fig S3). The vertical red dashed line indicates the time of stimulation with HGF; the vertical black dashed line corresponds to the calculated restriction point. t_R_: restriction point. a.u.: arbitrary units.

J-M Primary mouse hepatocytes were treated with 40 ng/ml HGF (red), left untreated (blue) (J), treated with different HGF concentrations (K), treated with 40 ng/ml HGF for a different amount of time (L, as shown in Fig1), or co-treated with 40 ng/ml HGF and different inhibitors (M). At the indicated time points (J) or after 72 h (48 h of treatment, K–M), cells were collected for DNA content measurement. DNA content was assayed using Sybr Green I. Open diamonds represent the mean of two to 17 scaled and merged biological replicates (four replicates per condition and time point were performed on average). Error bars were estimated based on the Sybr Green I data using a linear error model. Solid lines denote model trajectories, showing the 10 best results of 500 parameter estimation rounds. t_R_: restriction point. f.c.: fold change.

Source data are available online for this figure.

### Calibration of the mathematical model based on quantitative data

Since G1/S transition components increase despite the absence of growth factors in primary mouse hepatocytes, we hypothesized that the G1/S transition network contains tight control mechanisms for DNA synthesis. To disentangle these hepatocyte-specific properties, we calibrated our mathematical model based on our quantitative data sets (please refer to Supplementary Information, Calibration of the mathematical model, for detailed information). Figure4 shows the model simulations corresponding to the ten best parameter estimations as solid lines. The model trajectories are in accordance with the experimental data and quantitatively represent the dynamics of G1/S transition components in unstimulated and HGF-stimulated primary mouse hepatocytes (Fig4A–J). Furthermore, the model reproduces the extent of DNA synthesis for different doses of HGF (Fig4K) ranging from 0.2 to 40 ng/ml HGF and different stimulation times (corresponding to the experiment depicted in Fig1) (Fig4L). Additionally, the DNA content of primary mouse hepatocytes stimulated with 40 ng/ml HGF in combination with the CDK4 inhibitor PD0332991, the Akt inhibitor VIII, the MEK inhibitor U0126, or the p53 inhibitor Pifithrin α (inhibition of p21 induction) is in accordance with the model simulations (Fig4M). Thus, our model can explain the experimental data, indicating that our model assumptions are consistent with the regulation of G1/S transition in primary mouse hepatocytes.

Based on the ten best parameter sets, we investigated whether our model is able to uniquely describe the dynamics of G1/S transition components in primary mouse hepatocytes. Most of the G1/S transition components do not correspond to a single model species, but comprise several model variables. For example, a protein might participate in additional complexes or become phosphorylated at different sites. Therefore, even if a model can uniquely describe the observed species, other model species that are not amenable to experimental measurements may not be correctly described. If this was the case, the dynamics of the non-observed model species would be very different for each parameter set. To verify whether our model is able to uniquely describe the non-observed model species, we plotted the time-resolved dynamics of the internal model variables (Supplementary Fig S8B). For several variables, we could observe similar temporal dynamics in the absence or presence of HGF for the ten best parameter sets. This indicates that our model can provide information about species that are not directly experimentally addressable. In the same manner, we plotted the input variables Akt, ERK, GSK3β, and TF as a function of HGF (Supplementary Fig S8C). Interestingly, the input variable TF summarizing the transcription factors AP-1, Myc, and p53 displays rather high activation even at low doses of HGF.

To determine whether our model parameters can be uniquely determined, we performed an identifiability analysis as previously described (Raia *et al*, 2011). In Supplementary Fig S9, the estimated parameter values of the ten best parameter runs are shown as box plots. Additionally, all parameter values that were estimated over up to 12 orders of magnitude are depicted in Supplementary Fig S10. While not all parameter values can be uniquely determined, some parameters are identifiable, as indicated by the narrow extent of the corresponding box plot (Supplementary Fig S9). These analyses indicate that our calibrated mathematical model can be used to perform quantitative predictions, taking the detected uncertainties into account.

### Model analysis and experimental validation reveal CDK2 phosphorylation on T160 as gatekeeper for HGF-induced proliferation

**Figure 5:**
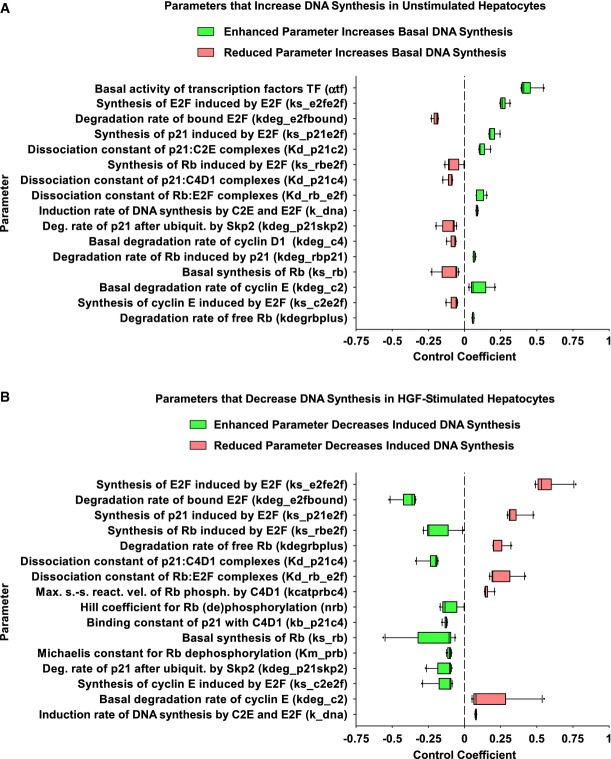
Basal activity of transcription factors and E2F auto-synthesis control basal and HGF-induced DNA synthesis, respectively

To unravel which parameters have a major impact on DNA synthesis, we performed sensitivity analyses. First, we calculated the parameter changes that increase DNA synthesis in unstimulated hepatocytes (Fig5A). The basal activity of the transcription factors TF is one of the most important parameters for basal DNA synthesis. Furthermore, an increase in auto-synthesis or a decrease in the degradation rate of Rb-bound E2F-1 enhances DNA synthesis. Similarly, we calculated parameters that decrease DNA synthesis in HGF-stimulated hepatocytes (Fig5B). Again, auto-synthesis and the degradation rate of Rb-bound E2F-1 have the highest control, followed by induction of p21 and Rb by E2F-1. This demonstrates the importance of the E2F-1 regulatory loop for the G1/S transition. While Fig5 summarizes the most important parameters for basal and HGF-induced DNA synthesis, Supplementary Fig S11 shows the control coefficients for all parameters.

Control coefficients of the model parameters with respect to basal DNA synthesis (DNA content at 72 h without stimulation) were calculated for the 10 best parameter estimations. Control coefficients are sorted by the absolute value of the median, and the 16 parameters with the largest absolute value of the median are shown as box plots (see Supplementary Fig S11 for all parameters).

Control coefficients of the model parameters with respect to induced DNA synthesis (DNA content at 72 h, 48 h of treatment with 40 ng/ml HGF) were calculated for the 10 best parameter estimations. Control coefficients are sorted by the absolute value of the median, and the 16 parameters with the largest absolute value of the median are shown as box plots (see Supplementary Fig S11 for all parameters). Box plots display the median, 10^th^, 25^th^, 75^th^, and 90^th^ percentiles as horizontal boxes with error bars. Outliers (5^th^ and 95^th^ percentiles) are shown as dots.

**Figure 6:**
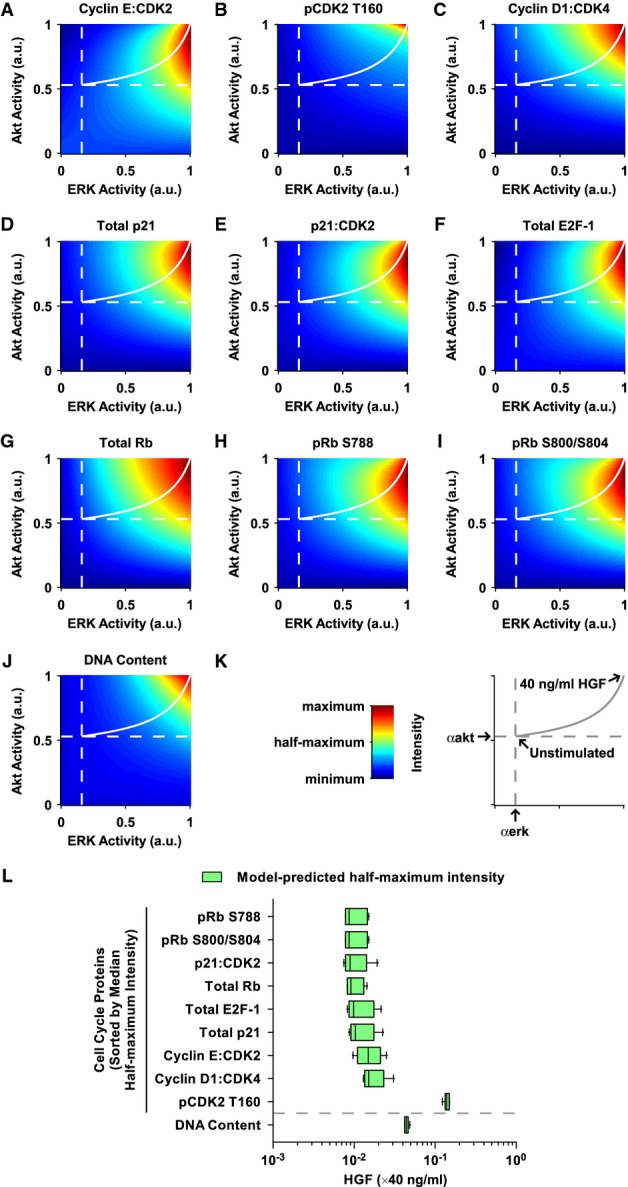
Distinct sensitivities of G1/S transition components to HGF concentrations predicted by the mathematical model

To identify the threshold mechanism that allows DNA synthesis only in HGF-stimulated primary hepatocytes, we calculated the intensity of all G1/S transition components and DNA synthesis (at *t* = 72 h/*t* = 48 h HGF) as a function of ERK and Akt activity. The intensity corresponds to the concentration of the complexes Cyclin E:CDK2 (Fig6A), Cyclin D1:CDK4 (Fig6C), and p21:CDK2 (Fig6E), and the total amounts of p21 (Fig6D), E2F-1 (Fig6F), and Rb (Fig6G) as well as the phosphorylation levels of pCDK2 T160 (Fig6B), pRb S788 (Fig6H), and pRb S800/S804 (Fig6I). The basal activities of ERK and Akt, as determined in Supplementary Fig S7, are indicated with dashed white lines. The intersection of these lines corresponds to the unstimulated scenario, while the top right corner of the plots (ERK activity = Akt activity = 1) coincides with the condition in which the cells were stimulated with 40 ng/ml HGF (Fig6K). Interestingly, many G1/S transition components display substantial intensity already in the presence of moderate ERK and Akt levels (Fig6A and C–I). However, highest intensity of pCDK2 T160 (Fig6B) and DNA content (Fig6J) can only be achieved if both ERK and Akt are fully active. We performed the same analysis with basal TF activity and HGF as inputs (Supplementary Fig S12A) and demonstrated, in line with our sensitivity analysis, that an increase of basal TF activity could induce DNA synthesis. However, the G1/S transition components react differentially to HGF and the basal TF activity. While most of the components can be strongly intensified by basal TF activity, pCDK2 T160 requires HGF for maximum intensity.

A-K The intensity of the cell cycle proteins and of the DNA content at 72 h was calculated as a function of ERK activity and Akt activity. Intensity corresponding to the concentration of the complexes Cyclin E:CDK2, Cyclin D1:CDK4, and p21:CDK2, the total amounts of p21, E2F-1, and Rb as well as the phosphorylation levels of pCDK2 T160, pRb S788, and pRb S800/S804 and the relative DNA amount is depicted with colors from blue (minimum intensity) to red (maximum intensity) for the cell cycle proteins. For DNA content, the range is displayed between one (no change) and two (doubling of the DNA). Basal activities of ERK and Akt as determined in Supplementary Fig S7 are indicated with dashed white lines. Solid white lines show the increasing activity of ERK and Akt with raising HGF concentration. Analysis is based on the best parameter estimation round. a.u.: arbitrary units.

L For each cell cycle protein and for DNA content, the HGF concentration inducing half-maximum intensity was calculated based on the 10 best parameter estimations (Supplementary Fig S12B). Cell cycle proteins are sorted by the absolute value of the corresponding median HGF concentration and displayed with the median and 10^th^, 25^th^, 75^th^, and 90^th^ percentiles as horizontal boxes with error bars. Outliers (5^th^ and 95^th^ percentiles) are shown as dots. The same analysis was performed for DNA content.

These analyses were based on the best parameter estimation run. To verify that the results are consistent and to quantify the dependence of the G1/S transition components on HGF, we calculated the intensity of the G1/S transition components and of the DNA content as a function of HGF for the ten best parameter estimations (Supplementary Fig S12B). We then determined the HGF concentration that is necessary for half-maximum intensity of each component. This model-predicted EC_50_ is plotted as a box plot in Fig6L. Interestingly, only little HGF is necessary for increasing the intensity of Rb and its phosphorylated forms, followed by p21 and E2F-1, and finally the Cyclin E:CDK2 and Cyclin D1:CDK4 complexes. On the other hand, the half-maximum intensity of CDK2 phosphorylated at T160 and also the DNA content require substantially higher concentrations of HGF.

**Figure 7:**
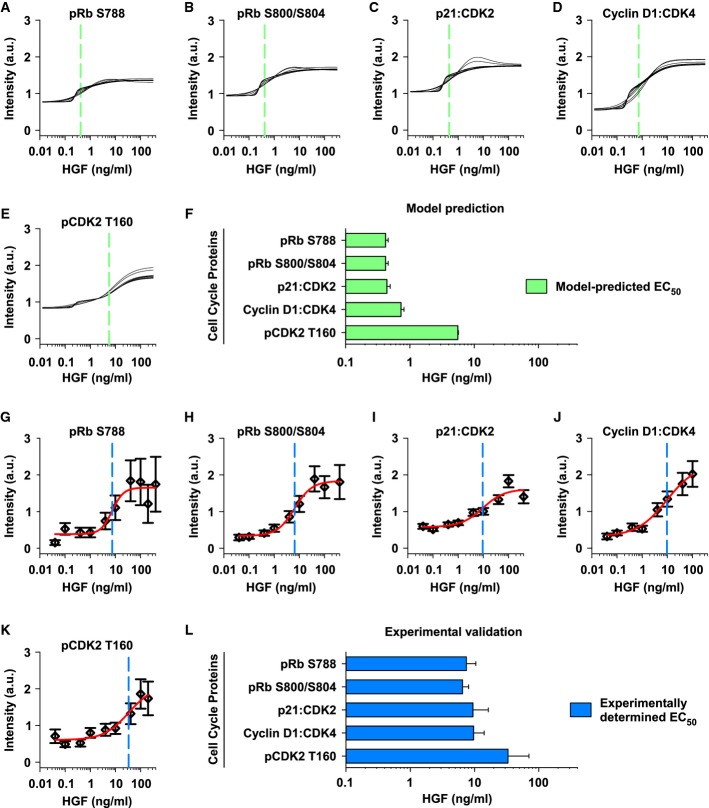
Experimental validation of pCDK2 T160 as gatekeeper for DNA synthesis in hepatocytes

To experimentally validate these model predictions, we analyzed the dependency of G1/S transition components on HGF concentration. We focused on the G1/S transition components that were addressable by robust measurement, that is, the complexes of p21:CDK2 and Cyclin D1:CDK4 as well as the phospho-species pRb 788, pRb S800/S804, and pCDK2 T160. Figure7A–E displays the model-predicted dose–response curves for these components assuming 24 h stimulation with HGF at concentrations ranging from 0.01 to 400 ng/ml indicating the EC_50_ as shown in Fig6L. The corresponding EC_50_ values for these five components (Fig7F) suggest a specific ranking with pCDK2 T160 requiring the highest HGF concentration. To experimentally verify our model predictions, we quantified these G1/S transition components by quantitative immunoblotting in primary mouse hepatocytes stimulated with HGF concentrations ranging from 0.04 to 400 ng/ml for 24 h (Supplementary Fig S13). For each averaged data set, we estimated a four-parameter Hill function that was calculated by performing a nonlinear regression of signal intensity as a function of HGF concentration (Fig7G–K). Interestingly, in line with our model predictions, pCDK2 T160 is the cell cycle component with the highest EC_50_ value (around 30 ng/ml HGF).

A-E The model-based intensity of the G1/S components pRb S788 (A), pRb S800/S804 (B), p21:CDK2 (C), Cyclin D1:CDK4 (D), and pCDK2 T160 (E) after 48 h of HGF stimulation was calculated as a function of HGF. Analysis is based on the ten best parameter estimation rounds. Vertical dashed green lines indicate half-maximum intensity.

F The average HGF concentration inducing half-maximum intensity was calculated, corresponding to the model-predicted EC_50_, and depicted with standard error of the mean.

G-K The concentration of the G1/S components pRb S788 (G), pRb S800/S804 (H), p21:CDK2 (I), Cyclin D1:CDK4 (J), and pCDK2 T160 (K) after 48 h of stimulation with different HGF concentrations was experimentally determined (Supplementary Fig S13). Experiments were performed at least three times. Data were processed, and error bars were estimated based on the quantitative immunoblot data using a linear error model (Supplementary Fig S3). Red lines depict a four-parameter Hill function that was calculated by performing a nonlinear regression of signal intensity as a function of HGF concentration. Vertical dashed blue lines indicate estimated inflection points of the regression function.

L EC_50_ values were calculated as the inflection point of the four-parameter Hill functions displayed in (G–K). Error bars represent standard errors of the estimated inflection points.

Source data are available online for this figure.

**Figure 8:**
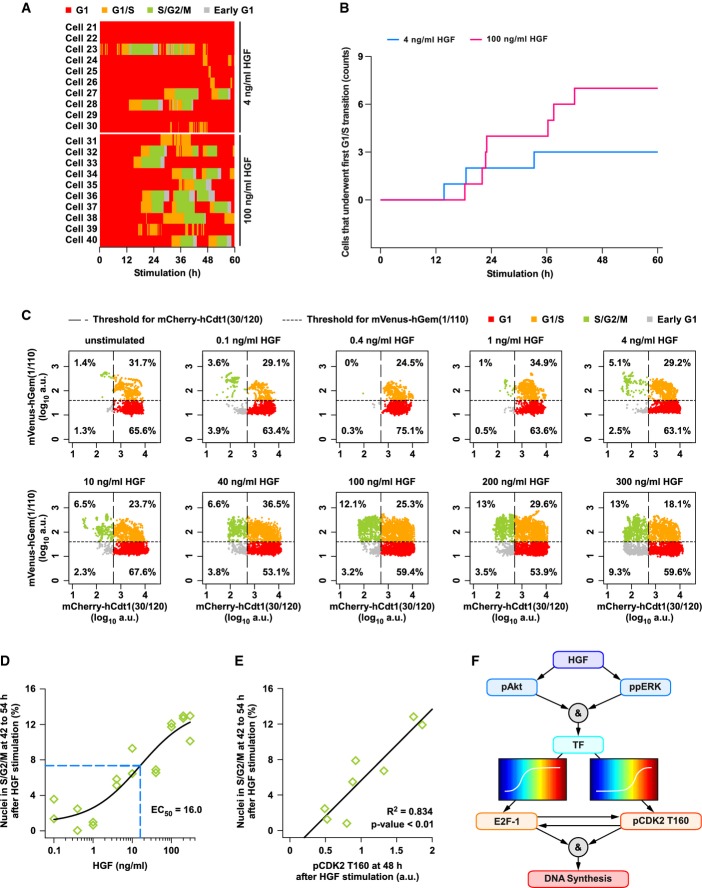
Phosphorylation of CDK2 at T160 correlates with dose-dependent proliferation of HGF-stimulated hepatocytes

To quantitatively link G1/S transition components to cell cycle entry, we analyzed the impact of different doses of HGF on hepatocyte proliferation at the single Fucci2 hepatocyte level. In principle, increasing concentrations of HGF could either induce proliferation at earlier times or increase the number of responding cells. To answer this question, we exemplarily selected 4 ng/ml HGF, a stimulus that induces moderate levels of DNA synthesis (Fig4K), and 100 ng/ml, a saturating concentration. We performed live cell imaging of Fucci2 primary mouse hepatocytes stimulated with these HGF concentrations and manually tracked 20 cells (Supplementary Fig S14A). We exemplarily show the G1, G1/S, S/G2/M, and early G1 phases of the cell cycle in a time-dependent manner for these 20 hepatocytes in Fig8A. Interestingly, displaying the number of cells that underwent G1/S transition at least once in a time-resolved manner for these two HGF doses (Fig8B) indicated that an increase in HGF concentration does not result in an accelerated entry into S phase. Rather, the higher HGF concentration caused an increase in the number of responding cells.

Primary mouse hepatocytes from mice transgenic for the Fucci2 cell cycle sensors (Fucci2 hepatocytes) were isolated, cultivated, and transduced with adeno-associated viral vectors encoding Histone2B–mCerulean to enable tracking of the cells. Live cell microscopy was performed with sampling rate of 15 min for up to 60 h, and 20 cells were tracked (Supplementary Fig S14A). The time-dependent cell cycle phases G1, G1/S, S/G2/M, and early G1 are displayed for primary mouse hepatocytes treated with 4 ng/ml HGF or 100 ng/ml HGF.

The number of cells that underwent G1/S transition at least once was quantified from the data displayed in (A) at each time point. Cell counts are displayed in a time-resolved manner (solid lines).

Live cell imaging was performed with Fucci2 hepatocytes stimulated with the indicated concentrations of HGF (sampling rate of 15 min for up to 60 h). Fucci2 signals of cell nuclei between 42 and 54 h after stimulation were plotted, and percentage of nuclei in the respective cell cycle phases were calculated based on criteria defined for the Fucci2 signals.

Percentages of Fucci2 primary mouse hepatocyte nuclei in S/G2/M phase (mCherry-hCDt1(30/120)^low^/mVenus-hGem(1/110)^high^) were plotted against HGF concentrations. A four-parameter Hill function was calculated by performing a nonlinear regression of the percentage in S/G2/M phase as a function of HGF concentration (black solid line). Dashed blue line indicates the estimated inflection point of the regression function. Data represent two biological replicates as displayed in (C) and Supplementary Fig S14B.

A linear regression of the average percentage of cells in S/G2/M phase at 42–54 h after HGF stimulation in dependence of the average pCDK2 T160 intensity at 48 h after HGF stimulation was performed. *R*^2^ and the Bonferroni adjusted *P*-value are shown (Supplementary Table S2).

A summary scheme of cell cycle information flow in primary mouse hepatocytes is displayed. The ampersands represent logical AND gates. Distinctive sensitivities of E2F-1 and pCDK2 T160 toward HGF are schematically shown.

Source data are available online for this figure.

To analyze the response of all hepatocytes to HGF at the single cell level, we segmented cell nuclei of Histone2B–mCerulean-labeled Fucci2 hepatocytes and quantified the Fucci2 markers in a time window from 42 to 54 h post-stimulation. We calculated the percentage of cells in the respective cell cycle phases using the same criteria for the Fucci2 cell cycle markers as above. As expected, the percentage of cells in S/G2/M phase was increasing as a function of HGF (Fig8C and Supplementary Fig S14B). The percentage of cells in S/G2/M phase in this snapshot analysis did not correspond to the total number of proliferating cells, because a cycling cell only remains in S/G2/M phase for a certain period of time, as shown in Fig8A. To determine the relationship between HGF concentration and the number of cycling cells, we plotted the number of hepatocyte nuclei in S/G2/M phase versus HGF concentration, performed a sigmoidal regression, and calculated the EC_50_ (Fig8D). The EC_50_ of 16 ng/ml HGF was close to the EC_50_ of pCDK2 T160 to HGF (Fig7K). This prompted us to hypothesize a linear relationship between pCDK2 T160 and induction of DNA synthesis. We therefore performed a linear regression between the experimentally measured phosphorylation levels of CDK2 at T160 (at 48 h post-HGF stimulation) and hepatocyte nuclei in S/G2/M (at 42–54 h post-HGF stimulation) for corresponding HGF concentrations. We indeed observed a highly significant linear relationship between these two experimental measurements. While all G1/S transition components increased with HGF and correlated with G1/S transition, the correlation with CDK2 T160 phosphorylation had the lowest *P*-value (Supplementary Table S2), confirming our model-predicted importance of pCDK2 T160 as gatekeeper for G1/S transition in hepatocytes.

We summarize our results in Fig8F. HGF induces phosphorylation of Akt and ERK, which in turn leads to induction of the transcription factors (TF) AP1, Myc, and p53. These factors promote an increase in E2F-1 and in the phosphorylation of CDK2 at T160, with complex auto- and cross-regulation. Because the dose–response profile of pCDK2 T160 displays low sensitivity toward upstream activators, it requires full activation of Akt and ERK to reach substantial levels. Free E2F-1 and pCDK2 T160 finally cooperate to induce DNA synthesis. Thus, we here identify a mechanism that allows DNA synthesis only if the Akt and ERK pathways are sufficiently activated by HGF. Taken together, we conclude that phosphorylation of CDK2 at T160 constitutes a threshold for safeguarding an HGF-induced G1/S transition in hepatocytes.

## Discussion

Under unperturbed conditions, the liver shows only a minimal rate of self-renewal that does not exceed 5% of the whole cell population (Guguen-Guillouzo, 2002). This is in line with the fact that this organ mainly fulfills metabolic functions, while proliferation is a rare event only induced upon severe liver damage and regulated by growth factors such as HGF. Here, by combining experimental data with mathematical modeling, we dissect the molecular mechanism that allows the G1/S transition and consequently DNA synthesis and proliferation to occur only in HGF-stimulated hepatocytes.

We have previously shown that in our *ex vivo* culture system, primary mouse hepatocytes complete DNA replication after 48 h of HGF stimulation (Huard *et al*, 2012). This result is congruent with *in vivo* data of partially hepatectomized mice where the peak of DNA synthesis occurs between 36 and 48 h post-surgery (Satyanarayana *et al*, 2004; Michalopoulos, 2007). Furthermore, we determined that the restriction point occurs after approximately 32 h of HGF stimulation in primary mouse hepatocytes. This is in line with gene expression studies addressing the G1/S transition after two-thirds PHx in C57BL/6 mice that identified a time frame of 30–36 h for this crossing point (Satyanarayana *et al*, 2004). These data indicate a good correlation for the timing of the G1/S transition and the restriction point between mouse hepatocytes *ex vivo* and *in vivo*. It has previously been reported that the peak of DNA synthesis is faster in rat than in mouse hepatocytes with a shift of 6–12 h (Michalopoulos, 2007). Primary rat hepatocytes have been shown to cross the restriction point after 40–44 h of growth factor stimulation (Loyer *et al*, 1996; Albrecht & Hansen, 1999). However, the experimental procedure in these two studies did not include an adhesion and growth factor depletion phase, but rather placed the cells directly in growth factor-supplemented medium following isolation. We determined the restriction point in primary mouse hepatocytes at 32 h after HGF stimulation, which corresponds to a total cultivation time of 62 h including adhesion, growth factor depletion, and stimulation phases. Thus, we conclude that similar to the timing of DNA synthesis, the timing of the restriction point is faster in rat than in mouse hepatocytes.

Recently, a bifurcation point at the end of mitosis was detected by a live cell sensor for CDK2 activity in constantly cycling cells (Spencer *et al*, 2013). In this report, it was discovered that a large fraction of MCF10A mammalian epithelial cells retains CDK2 activity and immediately commits to the next cell cycle. Additionally, CDK2 activity was shown to act as a threshold for the quiescence or proliferation decision. Interestingly, our single cell results in primary mouse hepatocytes are in line with these observations. We observed a linear correlation between CDK2 phosphorylation at T160 and proliferating hepatocytes for different concentrations of HGF, suggesting that CDK2 phosphorylation acts as a threshold in primary mouse hepatocytes. Additionally, we detected sustained high levels of pCDK2 T160 by quantitative immunoblotting (Fig4B) in the presence of 40 ng/ml HGF. In line with the findings of Spencer *et al* (2013), these sustained levels correlate with an immediate second round of replication for most of the cycling cells, as shown by single cell experiments (Fig1C).

In our study, we determined the dynamics of key G1/S transition components for up to 48 h of HGF stimulation in primary mouse hepatocytes. The obtained data indicated that while in some aspects the behavior of primary mouse hepatocytes correlated with the paradigms of cell cycle progression observed in most immortalized cell lines, some G1/S transition components feature exceptional kinetics in primary hepatocytes. A major concept of cell cycle progression is that the concentration of Cyclins varies depending on the cell cycle phase, while the levels of CDKs do not change over time. Concordantly, the amounts of CDK4 and CDK2 remained fairly constant during the G1 and early S phase in primary mouse hepatocytes. Cyclin D1 is known to act as a mitogenic sensor (Coqueret, 2002; Musgrove, 2006). While complexes were low in the absence of growth factor, Cyclin D1:CDK4 complex formation continuously increased upon HGF stimulation. The obtained data are in line with previous reports by Albrecht *et al* (1995, 1998) analyzing cell cycle progression upon two-thirds PHx in mice. In these studies, CDK levels did not change over time, and while Cyclin D1 was undetectable in the quiescent liver, levels of Cyclin D1:CDK4 complexes increased during 72 h post-surgery.

In our experiments, CDK4-bound p21 increased upon HGF stimulation in primary mouse hepatocytes. Interestingly, its dynamic was similar to that of Cyclin D1:CDK4, supporting a role of p21 as assembly factor for the otherwise inefficient formation of Cyclin D1:CDK4 complexes (Morgan, 1997). While total p21 levels were low in unstimulated cells, its amount increased upon HGF stimulation. These observations are consistent with the findings of Ilyin *et al* (2003) showing that in primary rat hepatocytes, p21 increased upon mitogen stimulation and was predominantly associated with Cyclin D1. Furthermore, p21 is induced upon two-thirds PHx in mice and co-precipitates with Cyclin D1 and CDK4 (Albrecht *et al*, 1998). Wierod *et al* (2008) furthermore demonstrated that in primary rat hepatocytes, EGF-induced p21 expression is PI3K dependent via the transcription factor p53 and that inhibition of p53 attenuates DNA synthesis and reduces Rb phosphorylation.

It has been postulated that Cyclin E is of key importance in enabling the transition from G1 to S phase, where it induces its expression by a self-amplifying feedback loop via E2F-1 (Sherr & Roberts, 1999). In a variety of cell lines, its expression peaks in S phase (Sherr, 1993; Moroy & Geisen, 2004). Strikingly, in primary mouse hepatocytes, the amount of Cyclin E:CDK2 complexes was only slightly increased during the G1 and early S phases. These data are, however, in line with the observation of Loyer *et al* (1996). In this report, no significant variation in Cyclin E:CDK2 complexes was observed in primary rat hepatocytes over time although the cells underwent DNA synthesis and thus completed S phase.

Our observation that p27 co-immunoprecipitating with CDK2 did not change significantly over time and that total p27 levels remained constant rather than decreased upon growth factor treatment is supported by similar reports for primary rat hepatocytes (Ilyin *et al*, 2003). Furthermore, expression of p27 has been shown to remain stable in regenerating mouse livers following two-thirds PHx (Albrecht *et al*, 1998). Hepatocytes usually only undergo two to three rounds of replication until the original liver mass is re-established (Michalopoulos, 2007). Thus, it might be possible that high p27 levels are retained to allow a rather quick return to quiescence once regeneration has been completed. Interestingly, in our data on Cyclin E:CDK2:p27 complexes, the only modification that showed a distinct change upon HGF stimulation was the activating threonine phosphorylation of CDK2 at T160. This suggests that in primary mouse hepatocytes, CDK2 kinase activity is mainly regulated via this CAK-dependent activation, which is supported by similar observations of Albrecht *et al* (1998) in regenerating mouse livers following two-thirds PHx.

The activity of the tumor suppressor Rb is mainly regulated by post-translational modification, that is, phosphorylation by CDKs, and its level remains constant in most immortalized cell lines during the course of the cell cycle (Fan *et al*, 1995; Herwig & Strauss, 1997). Thus, it was surprising to observe a fluctuation in the amount of total Rb during the G1 and early S phase of primary mouse hepatocytes. Fan *et al* (1995) investigated Rb expression in regenerating rat livers using a combination of immunoblotting and immunofluorescence microscopy. Interestingly, the authors reported a similar pattern of Rb expression after two-thirds PHx with a peak of nuclear Rb at 30 h after two-thirds PHx. Given the fact that *in vivo* liver regeneration progresses faster in the rat compared to the mouse (Weglarz & Sandgren, 2000; Michalopoulos, 2007), this observation nicely aligns with the quantitative immunoblot data presented in our study. Albrecht *et al* (1998) examined the kinase activity of CDK4 and CDK2 immunoprecipitated from regenerating mouse livers following two-thirds PHx in kinase assays using Rb-GST and histone H1 as artificial substrates. Interestingly, both kinases reached maximal activity at 36 h, that is, at the peak of DNA synthesis in this system. Congruently, in our HGF-stimulated primary mouse hepatocytes, we observe similar dynamics of both pRb S788 and pRb S800/S804 levels that indicate CDK4 and CDK2-specific phosphorylation, respectively.

The concentration of pathway components crucially determines the dynamic behavior of a system. Therefore, we determined the absolute concentrations of the main G1/S transition components by a recombinant calibrator protein standard curve. This analysis revealed that there is a sevenfold excess of CDK2 over CDK4 in primary mouse hepatocytes. This ratio could provide the basis for an amplification mechanism during the transition from G1 into S phase (Malumbres & Barbacid, 2001; Coqueret, 2002). The large difference in CDK2 and CDK4 levels could also be attributed to the distinct number of respective kinase substrates of either kinase. While the main target of CDK4 is the tumor suppressor Rb, CDK2 phosphorylates a great number of substrates (Malumbres & Barbacid, 2005). In a study by Chi *et al* (2008), approximately 180 potential CDK2 phosphorylation targets were identified by quantitative mass spectrometry in human cell lysates. This large difference between CDK4 and CDK2 is most likely attributed to the fact that CDK2 does form complexes not only with Cyclin E, but also with Cyclin A, and fulfills important functions in G1 as well as in the complete S phase to ensure proper DNA replication (Woo & Poon, 2003; Malumbres & Barbacid, 2005).

To elucidate our hepatocyte-specific observations on the G1/S transition further, we established a mathematical model based on differential equations connecting HGF-induced activation of signaling pathways, regulation of G1/S transition, and induction of DNA synthesis. The model was calibrated to our extensive time-resolved experimental data covering the dynamics of G1/S transition components as well as the DNA content in unstimulated and HGF-treated primary mouse hepatocytes. The DNA content measured under various conditions could be explained by the model, including the different HGF doses and pulses as well as the treatment with several small molecule inhibitors. Our dynamic experimental data were also nicely represented by the model simulations up to the restriction point and the timing of the G1/S transition. Future work could involve extending the model not only to describe the dynamics of cell cycle components of the G1/S transition, but to include the subsequent cell cycle phases as well. Furthermore, hepatocyte-specific ODE models describing HGF-dependent signal transduction could be implemented in more detail. Lastly, the p53 inhibitor Pifithrin α induces a certain amount of cell death as evidenced by the reduction of the DNA content below a value of 1. Because our mathematical model focuses on DNA synthesis rather than cell death, our model does not reproduce this data point.

Using a sensitivity analysis, we elucidated the critical reaction parameters in the G1/S transition of primary mouse hepatocytes. We focused on two aspects: DNA synthesis in unstimulated and HGF-stimulated hepatocytes. We here show that mainly the low levels of the transcription factors TF (Myc, AP-1, and p53) prevent DNA synthesis in unstimulated primary hepatocytes. If the activation of the Akt and ERK pathways and consequently the activity of these transcription factors TF cross a certain threshold, DNA synthesis occurs, which is then mainly controlled by the auto-synthesis of E2F-1.

We determined the absolute concentrations of the transcription factor E2F-1 at the restriction point to be only about 450 molecules per cell. Consistent with our observation, it has been shown that the levels of E2F-1 are very low in the quiescent hepatocytes of adult mice (Lukas *et al*, 1999). This indicates that E2F-1 is a limiting factor for the G1/S transition in primary hepatocytes. In line with this finding, the sensitivity analysis revealed a critical function of E2F-1 for HGF-induced DNA synthesis. The synthesis and degradation rate of E2F-1 as well as the parameters corresponding to gene expression induced by free E2F-1 showed the highest control over DNA synthesis in our system.

Our experimental data indicated a unique role for CDK2 phosphorylated at T160 in the G1/S transition control of hepatocytes. We elucidated the function of this species further using our mathematical model. Model analysis showed that while other G1/S transition components required only moderate ERK and Akt activity for maximal intensity, high signaling pathway activation and, thus, stimulation with sufficient amounts of HGF was necessary for full pCDK2 T160 induction. These results are in line with our experimental data showing that while a variety of G1/S transition components are induced upon hepatocyte isolation, and thus stress, DNA synthesis only occurs upon additional stimulation with HGF.

It has previously been suggested (Albrecht & Hansen, 1999) that Cyclin D1 expression is sufficient to promote progression through G1 in rat hepatocytes. On the other hand, Wierod *et al* (2008) reported that p21 expression is required for EGF-induced DNA synthesis in rat hepatocytes. Both results are in agreement with our findings of pCDK2 T160 as a gatekeeper, as CDK2 phosphorylation is a downstream process of both Cyclin D1:CDK4 activation and p21-mediated nuclear import of CDK2.

It is tempting to speculate why the T loop phosphorylation of CDK2 is more dependent on HGF stimulation than other species. We have implemented regulatory mechanisms in our model that resulted in a highly nonlinear CDK2 activation. While p21 is an activator of Cyclin D1:CDK4, it has a dual role with respect to Cyclin E:CDK2, being both activator and inhibitor. Consequently, because Cyclin E:CDK2 depends in a nonlinear manner on p21 concentrations, we hypothesize that particularly high HGF concentrations might be required for full CDK2 activation. While the Cyclin E:CDK2 complex is part of nonlinear regulatory mechanisms requiring high HGF concentration to be triggered, the activation of Cyclin D1:CDK4 depends rather linearly on the HGF concentration. While it does not exclude other mechanisms, the regulatory mechanisms in our model are sufficient to explain the predicted gatekeeper function of pCDK2 T160.

Importantly, we experimentally validated our model prediction using both population measurements and single cell data. At the population level, pCDK2 T160 was the G1/S transition component with the highest EC_50_ concerning HGF sensitivity, as suggested by the model. In general, the experimentally determined EC_50_ values of all analyzed G1/S transition components were approximately one order of magnitude higher than predicted. This might be due to the fact that we assumed a linear relationship between intracellular signaling, cell cycle regulation, and DNA synthesis. Nevertheless, the ranking of the experimentally derived EC_50_ values was in line with our model predictions. Our single cell experiments resulted in additional insights. At 48 h after stimulation, the EC_50_ value of CDK2 phosphorylation to HGF concentration was close to the EC_50_ value of hepatocyte nuclei in S/G2/M phase to HGF dose. In line with this result, we identified a linear relationship between the levels of CDK2 phosphorylated on T160 and hepatocyte nuclei in S/G2/M phase at this time point over the range of tested HGF concentrations.

Taken together, the dynamics of key G1/S transition species in primary mouse hepatocytes are distinct from the behavior observed in most mammalian immortalized cell lines, and these differences are not only observed in culture, but also *in vivo* following removal of two-thirds of the liver. Thus, the data presented here support a key mechanism for the regulation of the G1/S transition in hepatocytes, where phosphorylation of CDK2 at T160 acts as a gatekeeper preventing the transition from G1 to S phase upon cellular stress cues in the absence of proliferative signals such as HGF.

## Materials and Methods

### Chemicals

If not stated otherwise, chemicals were purchased from Sigma-Aldrich.

### Isolation of primary mouse hepatocytes

Primary mouse hepatocytes were isolated as described by Huard *et al* (2012). For isolation, 8- to 12-week-old male C57BL/6N mice (Charles River) housed at the DKFZ animal facility under a constant light/dark cycle, maintained on a standard mouse diet, and allowed *ad libitum* access to food and water were used. All animal experiments were approved by the governmental review committee on animal care of the state Baden-Württemberg, Germany (reference number A24/10). For the cell cycle components analysis, hepatocytes were seeded at subconfluence (2 × 10^6^ cells/10-cm dish) in full medium (phenol red-free Williams E medium (Biochrom) supplemented with 10% (v/v) fetal bovine serum (Life Technologies), 0.1 μM dexamethasone, 10 μg/ml insulin, 2 mM l-glutamine, and 1% (v/v) penicillin/streptomycin 100× (both Life Technologies) using collagen I-coated cell ware (BD Biosciences). Hepatocytes were cultured at 37°C, 5% CO_2_, and 95% relative humidity. Following cell adhesion, hepatocytes were washed with PBS (PAN Biotech) and subsequently cultivated in serum-free cultivation medium (phenol red-free Williams E medium supplemented with 0.1 μM dexamethasone, 2 mM l-glutamine and 1% (v/v) penicillin/streptomycin 100×). For the analysis of the basal activity of ERK and Akt, hepatocytes were seeded at confluence (2 × 10^6^ cells/6-cm dish) in full medium as described above. Following cell adhesion, hepatocytes were washed with PBS (PAN Biotech) and subsequently cultivated in serum-free cultivation medium (phenol red-free Williams E medium supplemented with 2 mM l-glutamine and 1% (v/v) penicillin/streptomycin 100×) for 6 h prior treatment.

### Sybr Green assay

Primary mouse hepatocytes were seeded at subconfluence (125,000 cells/well of six-well plate), and the Sybr Green assay was performed as described (Huard *et al*, 2012). After 24 h in serum-free cultivation medium, hepatocytes were washed with PBS, received fresh medium, and were stimulated with the indicated doses of rmHGF (R&D Systems) or remained untreated. For inhibitor experiments, cells additionally received 10 μM Akt inhibitor VIII (Merck Millipore), 10 μM U0126 (Cell Signaling Technology), 10 μM PD0332991 (Selleck Chemicals), 30 μM Pifithrin α (Merck Millipore), or equal volumes of DMSO. At indicated time points, cells were washed with PBS and frozen at −20°C for at least 24 h. To assay DNA content, cells were incubated with Sybr Green (Life Technologies) and fluorescence was read with λ_excitation_ = 485 nm and λ_emission_ = 535 nm.

### C57BL/6N-Fucci2 mice breeding and genotyping

R26p-Fucci2 mice were obtained from RIKEN Center for Developmental Biology (CDB) and recovered by embryo transfer. Heterozygous C57BL/6N-Fucci2 mice were bred with wild-type C57BL/6N to maintain the line. Genotyping PCR was performed with three primers in one reaction:

Forward: atggtgagcaagggcgaggag, mCherry_rev: catgaactgaggggacagga, mVenus_rev: gcttggactggtagctcagg.

### AAV vector production

AAV vectors were produced using a standard triple transfection protocol (Grimm, 2002). Briefly, an AAV helper plasmid encoding AAV rep and cap genes and an AAV vector plasmid carrying the transgene as well as an adenoviral helper plasmid were transfected into HEK293T cells. The cap gene in the AAV helper was derived from wild-type AAV serotype 9 through insertion of a short DNA oligonucleotide encoding a heptamer peptide. Human Histone2B (Weidemann *et al*, 2003) was kindly provided by Jörg Langowski, and mCerulean (Rizzo *et al*, 2004) was kindly provided by David W. Piston. The Histone2B–mCerulean cassette was amplified by PCR, digested with AgeI and AvrII restriction enzymes, and ligated into the AAV vector plasmid. The AAV vector plasmid expressing a fusion of Histone2B and mCerulean under the control of a cytomegalovirus (CMV) promoter was based on pSSV9, a plasmid that is routinely used for generation of single-stranded AAV vectors (Samulski *et al*, 1987). For the triple transfection, ten 15-cm^2^ dishes with 4 × 10^6^ HEK293T cells per dish were seeded 2 days prior to transfection. Using polyethylenimine (PEI) as transfection reagent, the cells were then triple-transfected with 14.6 μg of each plasmid per dish. 43.8 μg total DNA was diluted in DMEM without any supplements, mixed with 140 μl of PEI (1 mg/ml in H_2_O), and incubated for at least 30 min at room temperature. This mixture was next added dropwise to the cells and incubated at 37°C for 3 days, before the cells were harvested into the medium and pelleted at 400× *g* for 15 min. After one wash step with PBS, the pellet was resuspended in 6 ml lysis buffer (50 mM Tris–HCl pH 8.5, 50 mM NaHCO_3_) and subjected to five rounds of freezing and thawing −80°C/37°C). Following digestion of the cell lysate with 50 U/ml benzonase for 1 h at 37°C, cell debris was removed by centrifugation at 4,000× *g* for 20 min. For purification, the virus-containing lysate was added to a preformed gradient of 15, 25, 40 and 60% iodixanol (OptiPrep in PBS-MK; PBS with 1 mM MgCl_2_, 2.5 mM KCl) and centrifuged in a 70.1 Ti rotor (Beckman Coulter) at 171,000× *g* and 4°C for 2 h. Using needle and syringe, purified viral particles were retrieved from the 40% phase and stored in 50 μl aliquots at −80°C. A vector titer of 9.17 × 10^10^ genome copies per ml was determined using standard RT–PCR.

### Live cell imaging

Primary hepatocytes were seeded at subconfluence into 96-well plates pre-coated with collagen I (10 ± 2.5 × 10^3^ cells/well) in adhesion medium, supplemented with purified AAV encoding Histone2B–mCerulean (MOI between 7.34 × 10^3^ and 12.33 × 10^3^). Four hours after seeding, cells were washed once with pre-starvation medium and incubated in pre-starvation medium for 24 h before HGF stimulation. Shortly before time-lapse microscopy, media were changed to 100 μl fresh pre-starvation medium per well. Stimulation was performed by adding 100 μl 2× concentrated HGF diluted in pre-starvation medium. Hepatocytes were imaged with a Nikon Eclipse Ti Fluorescence microscope controlled with NIS-Elements software. Temperature (37°C), CO_2_ (5%), and humidity were held constant by an incubation chamber enclosing the microscope and a 96-well plate stage insert. Four channels were acquired for nine positions per well: brightfield channel, CFP channel (Histone2B–mCerulean), RFP channel (mCherry-hCdt1), and YFP channel (mVenus-hGem).

### Image analysis

Fiji software was used for image analysis. Background subtraction was performed with rolling ball method. For the triple-color data set, CFP channel was used for segmentation of nuclei using a standard thresholding-based algorithm. Mean RFP and YFP intensity was calculated for all segmented nuclei across all time points.

### Scatter plot analysis of Fucci2 data

To generate scatter plots for the analysis of the Fucci2 data, we applied filters to remove unsuitable fields. To achieve this, we used the RFP image at the time point 0 to segment nuclei and calculate the count of seeded nuclei, and CFP image at the time point 0 to calculate the number of infected nuclei. Only positions where 10–50 nuclei were seeded and at least 5 nuclei were infected with AAV encoding Histone2B–mCerulean were included in the scatter plots. We analyzed all segmented nuclei within a time window between 42 and 54 h after stimulation (12 h duration) to quantify percentage of subpopulations. We defined criteria for four subpopulations based on thresholds for RFP and YFP using unstimulated condition as a reference. The percentage of S/G2/M, namely RFP^low^/YFP^high^, was used for the estimation of the dose–response curve and for the regression analysis. The experiment was performed in independent biological duplicates.

### Single cell tracks

Manual segmentation and tracking were performed to obtain single cell tracks. Segmentation was performed by drawing region of interest (ROI) in individual nuclei in the image of CFP channel (H2B–mCerulean) frame by frame. When hepatocytes divide, only one of its daughter cells was followed until the end of the whole time lapse. The set of ROIs for single cells were copied to the RFP and YFP channels and mean RFP and YFP intensity was extracted after background subtraction. To better visualize and quantify cell cycle transitions, the criteria defined in the scatter plot analysis of Fucci2 data were employed to assign the cells to four different states, RFP^high^/YFP^low^, RFP^high^/YFP^high^, RFP^low^/YFP^high^, or RFP^low^/YFP^low^, which were represented by red, orange, green, and gray in a heatmap, respectively. To quantify G1/S transition events, we applied a logical function to identify G1/S transition, corresponding to four continuous time points with G1/S phase followed by four continuous time points with S/G2/M phase. False-negative transitions were manually corrected. Numbers of cells with at least one G1/S transition and accumulative numbers of G1/S transitions were plotted over time.

### Quantitative immunoblotting

After 24 h in serum-free cultivation medium, hepatocytes were washed with PBS, received fresh medium, and were stimulated with 40 ng/ml rmHGF or remained untreated. To determine the basal activity of ERK and Akt, hepatocytes were treated for 30–45 min with 20 μM U0126 (Cell Signaling Technologies) or 5 μM PI-103 (Calbiochem) prior stimulation with 40 ng/ml of rmHGF. To stop stimulation at designated time points, the medium was aspirated and cells were lysed on ice using total cell lysis buffer (50 mM Tris pH 7.4, 150 mM NaCl, 1 mM EDTA pH 8.0 (AppliChem), 1% (v/v) NP-40 (Roche Applied Sciences), 0.1% (w/v) sodium deoxycholate, 1 mM Na_3_VO_4_, 5 mM NaF, 0.1 mg/ml AEBSF, 1 μg/ml aprotinin). Samples for the cell cycle components analysis were additionally subjected to pulsed sonication. Supernatants constituting total cellular lysates were used for ppERK and pAkt analysis or they were subjected to immunoprecipitation (IP) using 1,000 μg of total protein. For IP α-CDK2, α-CDK4, α-Cyclin E (all Santa Cruz Biotechnology), α-E2F-1 (Cell Signaling Technology), α-p21, α-p27, or α-Rb (all BD Biosciences), antibodies were used. Recombinant calibrator proteins were spiked into IP reactions to enable subsequent data normalization or calculation of molecules per cell. For the control experiments using blocking peptides, we acquired the respective blocking peptides for the antibodies directed against CDK2, CDK4, and Cyclin E (all Santa Cruz) and pre-incubated the antibodies with a fivefold (by weight) excess of blocking peptide according to the manufacturer's instructions. For the control experiments using competing amounts of recombinant proteins, we pre-incubated the antibodies against Cyclin D1, Rb, and p21 with 500 ng SBP-Cyclin D1, 100 ng GST-Rb, and 1,000 ng SBP-p21, respectively, corresponding to a 50- to 100-fold excess compared to the endogenous proteins.

Precipitated proteins or total cellular lysates were subjected to SDS–PAGE and blotted on PVDF (CDK2-, CDK4-, p21-, p27-, and Rb-IP, ppERK, pAkt) (Merck Millipore) or nitrocellulose (E2F-1) (Whatman) membranes. Membranes were probed using α-E2F-1, α-pRb S807/811, α-pRb S795, α-pCDK2 T160, α-p27, a-α-p44/42 MAPK T202/Y204, α-pAkt S473 (Cell Signaling Technology), α-actin (Sigma), α-PDI (Enzo Life Sciences), α-Rb, α-p21 (BD Biosciences), α-CDK2, α-CDK4, α-Cyclin D1 (Santa Cruz Biotechnology), or α-Cyclin E (Merck Millipore) antibodies. Target proteins were visualized using enhanced chemiluminescence, and signals were acquired using a CCD camera-based device (ImageQuant LAS 4000 biomolecular imager, GE Healthcare). Immunoblot data were quantified using ImageQuant TL version 7.0 software (GE healthcare).

### Quantitative bead-based multiplex assay

Hepatocytes were seeded at confluence (2 × 10^6^ cells/6-cm dish) in full medium as described above, and following cell adhesion were washed with PBS (PAN Biotech) and subsequently cultivated in serum-free cultivation medium (phenol red-free Williams E medium supplemented with 2 mM l-glutamine and 1% (v/v) penicillin/streptomycin 100×) for 6 h prior to 10 min stimulation with the indicated doses of rmHGF. To stop stimulation, the hepatocytes were treated as described above. Supernatants constituting total cellular lysates were incubated over night with beads coupled with pAkt S473 antibody (Bio-Rad) and assayed with the Bioplex phosphoprotein detection kit (Bio-Rad). Washing procedures were performed using the Bio-Plex Pro™ II Wash Station (Bio-Rad). The pAkt S473 fluorescence intensity of the analyzed samples was acquired using the Bio-Plex Pro™ II instrument (Bio-Rad).

### Computational data processing and error estimation

All experiments were performed in biological replicates, and a linear error model was established based upon the method previously described (Raia *et al*, 2011) to estimate the relative error for each observation. Quantitative immunoblotting and Sybr Green assay data were processed using the software GelInspector to allow scaling of different data sets (Schilling *et al*, 2005). Scaling was performed using smoothing spline estimates, calculated as MATLAB csaps splines with a smoothness of 0.5. For each data point, between 2 and 7 biological replicates were available, where each replicate originates from hepatocytes of an individual animal. Quantitative immunoblotting data sets were additionally normalized employing the recombinant calibrator signal of the immunoprecipitation as described (Schilling *et al*, 2005). Subsequent to normalization and scaling, all data sets were merged by calculating the mean signal strength of each data point. In the case where three or more biological replicates were available, the standard deviation of the respective data point was calculated. For quantitative immunoblotting data, a linear error model was applied, meaning that a constant relative error was assumed for increasing signal strength. The mean signal strength was plotted against the corresponding standard deviation, and a linear regression without offset was performed of which the slope corresponded to the relative error. To calculate the absolute estimated error, data were multiplied with the calculated relative error and divided by the square root of the number of replicates (corresponding to standard error of the mean). The error distribution of the Sybr Green assay data was compatible with a constant error model. Therefore, the average standard deviation was calculated based on the complete data set for DNA content data. To prevent numerical problems during parameter estimation due to different scaling of the arbitrary units, the average intensity of each observation was set to 1 for all G1/S transition components. For DNA content measurements, the intensity at 24 h (time point of stimulation) was set to 1. For the EC_50_ calculations, quantitative immunoblotting data were merged as described above, and the EC_50_ was calculated as the inflection point of a four-parameter Hill function by performing a nonlinear regression of signal intensity as a function of HGF concentration. EC_50_ values are displayed with standard error of the estimated inflection points.

### Model construction and parameter estimation

The rules describing the interactions in the HGF-regulated G1/S transition model were formulated based upon established literature knowledge using the software *BioNetGen 2.2.2* (Faeder *et al*, 2009). The resulting model was exported in SBML format with a slightly modified version of the export routine and then imported to the *MATLAB* toolbox *PottersWheel 3.0.11* (Maiwald & Timmer, 2008) with appropriate modifications (both scripts are available on demand). The final model contains 24 ODEs, 69 reactions, 55 kinetic parameters, five inputs, 10 observables, and nine scaling parameters. Parameter estimation was performed using the *MATLAB* toolbox *PottersWheel 3.0.11* (Maiwald & Timmer, 2008). Parameters were estimated in logarithmic (log_10_) parameter space employing a trust-region algorithm and using *RADAU* with fast integration. For each parameter estimation run, up to 300 iterations with a χ^2^ tolerance of 10^−5^ and fit parameters tolerance of 10^−5^ were performed. Each parameter estimation run was started with parameter values that were disturbed with a strength of *s* = 1.5, so that *p*_new_ = *p*_original_ × 10^(*s* × ε)^ with ε being normally distributed with mean 0 and variance 1. One thousand parameter estimation runs were carried out in total, and the best ten runs were selected for further analysis. Fifty-two parameters were estimated (45 kinetic rate constants, four scaling parameters, and three parameters describing the input variables). The calibrated model is provided as SBML file and is available to the community at the Biomodels Database (MODEL1502090000).

### Sensitivity analysis

Each parameter was decreased by a factor of 10%, and DNA content at *t* = 72 h was then simulated with and without HGF set to 1. The same process was repeated with a 10% parameter increase. The control of each parameter over DNA synthesis at *t* = 72 h was calculated using the formula [*x*(*f* × *p*) − *x*(*p*)] / [(*f* − 1) × *x*(*p*)] where *x*(*p*) denotes the value of DNA at *t* = 72 h and *f* the factor used to perturb the parameter values. Finally, a ranking of the most influential parameters in two different contexts (increase of DNA content in unstimulated cells and decrease in stimulated cells) was established by sorting of the control coefficients with the correct sign.

### Identification of threshold mechanism

A dose–response for all species was simulated with HGF as input by defining the response as being the maximal value over a 72-h time period. The dose of half-maximum intensity was then evaluated. This process was repeated over the 10 best fits. Median values and standard deviation were then computed for every species, and a sequence of activation was determined by ranking the species according to their median doses for half-maximum intensity.

## Supporting Information

Supplementary Dataset S1

Review Process File

Source Data for Figure 1B

Source Data for Figure 1E

Source Data for Figure 2A–D

Source Data for Figure 2E–G

Source Data for Figure 2H

Source Data for Figure 2I, J

Source Data for Figure 2K

Source Data for Figure 2L–N

Source Data for Figure 2O

Source Data for Figure 4A–I

Source Data for Figure 4J

Source Data for Figure 4K

Source Data for Figure 4L

Source Data for Figure 4M

Source Data for Figure 7G

Source Data for Figure 7H

Source Data for Figure 7I

Source Data for Figure 7J

Source Data for Figure 7K

Source Data for Figure 8D

Source Data for Figure 8E
