# Supplementary material for: T160-phosphorylated CDK2 defines threshold for HGF-dependent proliferation in primary hepatocytes
Source: Mol Syst Biol. 2015 Mar 14;11(3):0795. doi: 10.15252/msb.20156032 (PMC4380929; doi:10.15252/msb.20156032)
Supplement: Supplementary file 1 [file msb0011-0795-sd1.pdf]

Supplementary Information

T160-phosphorylated CDK2 defines threshold for HGF-dependent proliferation in primary hepatocytes

Stephanie Mueller[§], Jérémy Huard[§], Katharina Waldow, Xiaoyun Huang, Lorenza A. D'Alessandro, Sebastian Bohl, Kathleen Börner, Dirk Grimm, Steffen Klamt, Ursula Klingmüller[*], Marcel Schilling[*]

Contents

1 Supplementary Tables **2**
1.1 Table S1 2
1.2 Table S2 2

2 Supplementary Figures **3**
2.1 Figure S1 3
2.2 Figure S2 4
2.3 Figure S3 5
2.4 Figure S4 6
2.5 Figure S5 7
2.6 Figure S6 8
2.7 Figure S7 9
2.8 Figure S8 10
2.9 Figure S9 11
2.10 Figure S10 12
2.11 Figure S11 13
2.12 Figure S12 14
2.13 Figure S13 15
2.14 Figure S14 16

3 Description of the model species and regulatory mechanisms **17**
3.1 Cyclins and cyclin-dependent kinases 17
3.2 p21 17
3.3 Rb 17
3.4 E2F-1 and DNA synthesis 17

4 Description of the model's ODEs **18**
4.1 Definition of the rules used to generate the ODEs 18
4.2 Species definitions used in the reaction rates 22

5 Algebraic equations **23**
5.1 Algebraic equations describing the signal transduction pathways 23
5.2 Definitions of the model's observables 23

6 Parameter descriptions and start values before fitting **24**

7 Determination of scaling factors and initial values **27**
7.1 Cytoplasmic and nuclear volumes of primary mouse hepatocytes 27
7.2 Scaling factors 27
7.3 Initial values 28

8 Calibration of the mathematical model **28**

Supplementary References **29**

1 Supplementary Tables

1.1 Table S1

Function	$y = y_0 + a \cdot \frac{x^b}{c^b + x^b}$				
**Replicate**	R^2	y_0	a	b	c
**1**	0.90	15781	16895	3.802	**31.59**
**2**	0.79	11453	13673	3.139	**29.52**
**3**	0.87	10636	15967	3.946	**34.44**
				**Average t_R [h]**	**31.85 ± 2.47**
				**Average t_R [h:min]**	**31:51 ± 2:28**

Table S1. The restriction point in primary mouse hepatocytes was calculated based on the inflection point of a four-parameter Hill function. A nonlinear regression of DNA content measured by Sybr Green I (y) as a function of time (x) was performed. The experiment was performed in biological triplicates and the restriction point (t_R) was calculated as the average inflection point (c) of the regression.

1.2 Table S2

Function	$y = \mathit{offset} + \mathit{slope} \cdot x$						
**Regression**	*n*	*offset*	*slope*	R^2	*adj.* R^2	*p-value*	*adj. p-value*
**pRb S788**	8	-0.0577	6.1484	0.6034	0.5373	0.02336	**0.11147**
**pRb S800/S804**	7	-0.1048	5.3980	0.6986	0.6383	0.01917	**0.09224**
**p21:CDK2**	7	-2.8960	8.1150	0.8491	0.8189	0.00318	**0.01580***
**CyclinD1:CDK4**	7	-1.2840	5.9240	0.8732	0.8479	0.00204	**0.01015***
**pCDK2 T160**	8	-2.4200	8.0480	0.8341	0.8064	0.00153	**0.00761****

Table S2. A linear regression of percentage of cells in S/G2/M phase (y) at 42 to 54 h after HGF stimulation as a function of the G1/S transition components (x) at 48 h after HGF stimulation was performed. Significance of the correlation based on Bonferroni adjusted p-values is shown (* < 0.05; ** < 0.01). Statistics was performed with the *lm* function in R [1].

2 Supplementary Figures

2.1 Figure S1

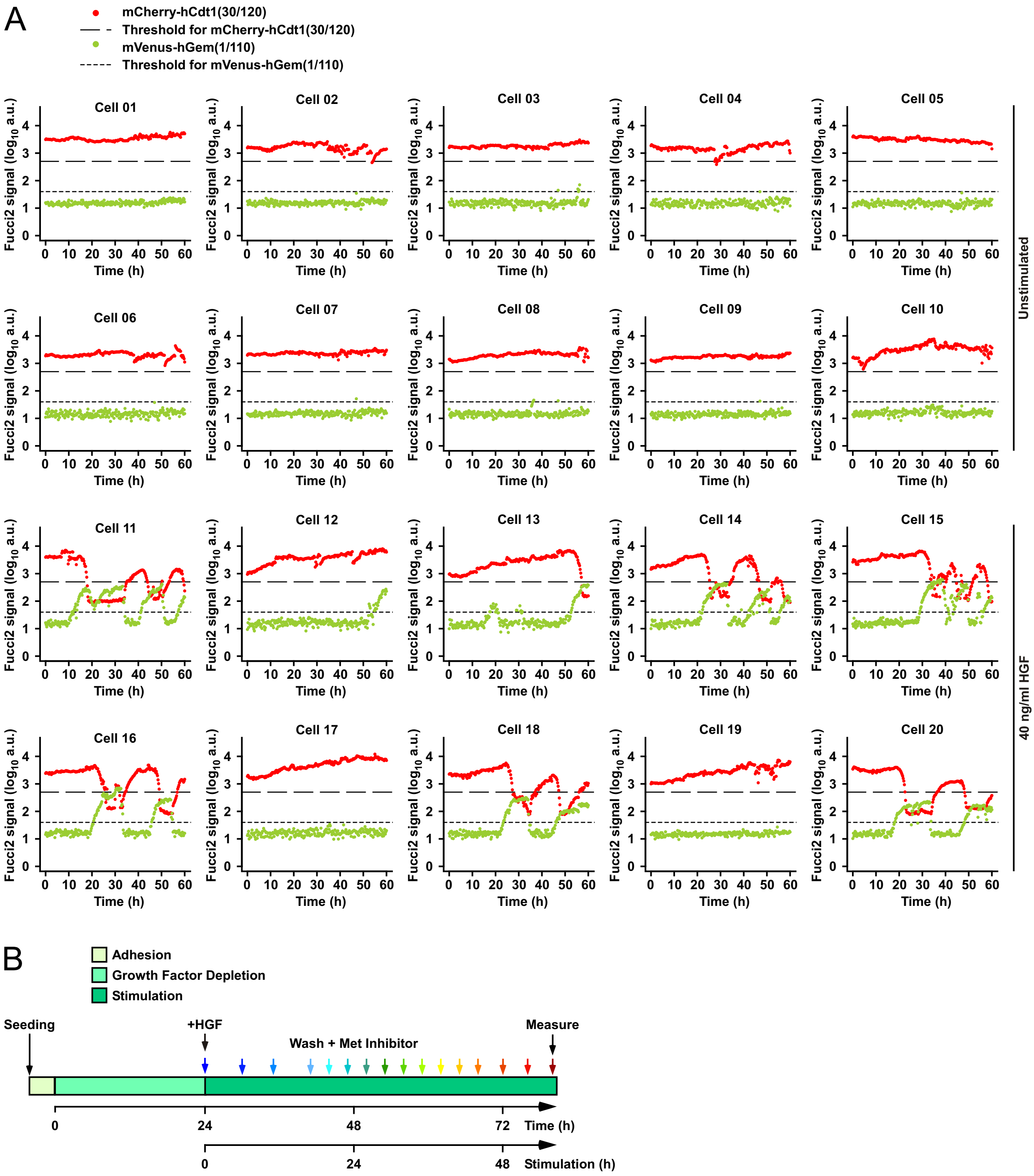

Figure S1. Single cells tracks of Fucci2 primary mouse hepatocytes and pulsed stimulation of primary hepatocytes to determine the restriction point in primary mouse hepatocytes. (**A**) Primary mouse hepatocytes of C57BL/6N-Fucci2 animals were transduced with Histone2B-mCerulean by an adeno-associated virus-based protocol to enable tracking of the cells. Live cell microscopy was performed of the Fucci2 primary mouse hepatocytes stimulated with 40 ng/ml HGF or left untreated (sampling rate of 15 min for up to 60 h) and 20 cells were manually tracked. Thresholds were defined for the Fucci2 signals. Figure 1C shows the cell cycle phases of this data set. (**B**) Primary mouse hepatocytes were stimulated with 40 ng/ml HGF 24 h after isolation or remained untreated for the entire experiment. After distinct time intervals (color coded) cells were washed three times with PBS, and received stimulus-free cultivation medium supplemented with 2.5 μM PHA 665752 c-Met inhibitor. Cultivation was continued for a total time of 80 h and cells were collected for DNA content measurement.

2.2 Figure S2

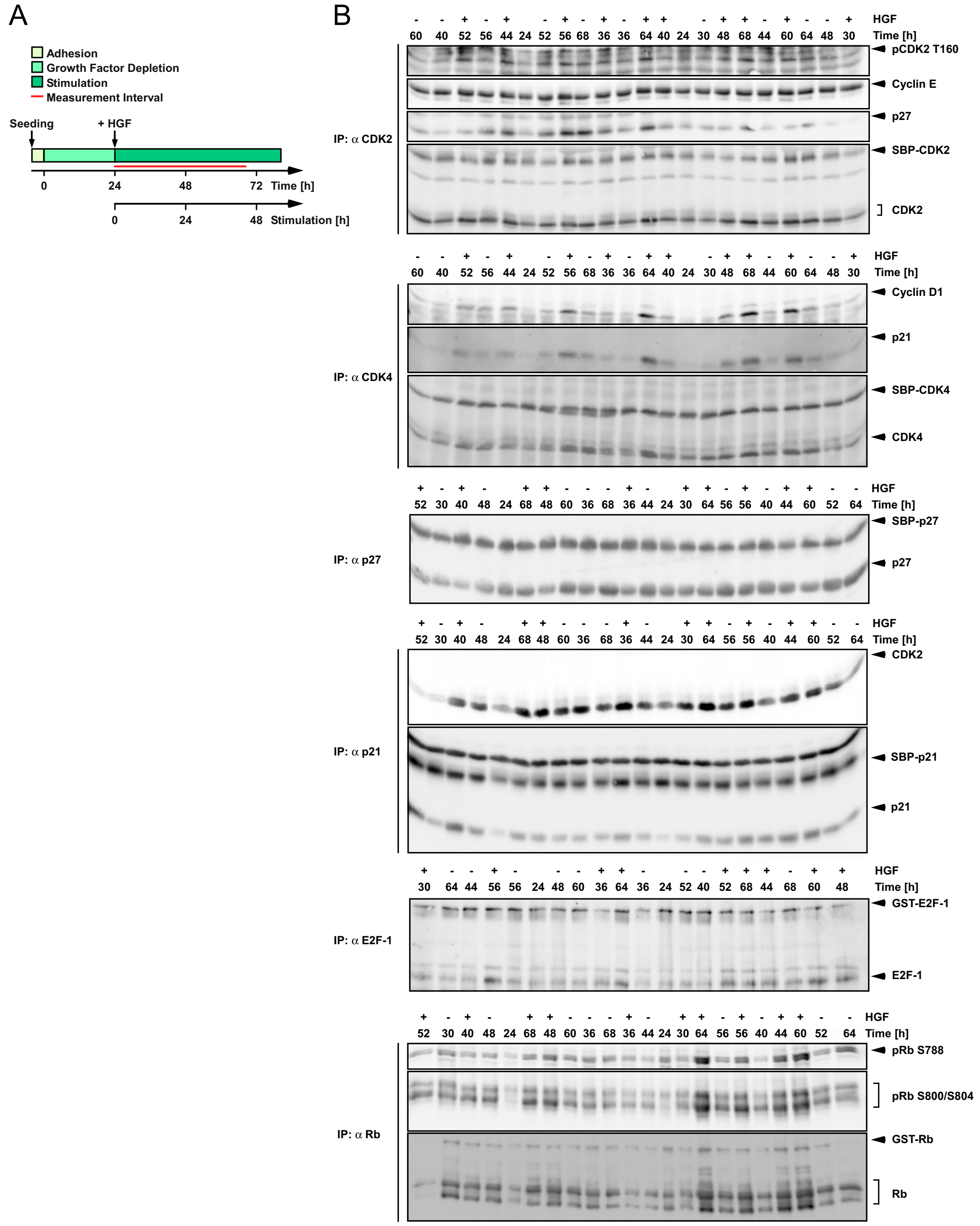

Figure S2. Quantitative immunoblot data of HGF-stimulated primary mouse hepatocytes. (**A**) Primary mouse hepatocytes were isolated by liver perfusion and cultivated at subconfluence according to the depicted scheme. After 24 h, hepatocytes were stimulated with 40 ng/ml HGF or remained untreated. Cells were lysed at different time points within the measurement interval indicated by the red solid line. (**B**) Time-resolved investigation of G1/S transition components by quantitative immunoblotting was performed. Primary mouse hepatocytes were stimulated with 40 ng/ml HGF or remained untreated, and cells were lysed using total cellular lysis buffer at the indicated time points. Proteins were subjected to immunoprecipitations (IP) with the indicated antibodies. To allow for data normalization, recombinant GST- or SBP-tagged calibrator proteins were added to the lysates before performing the IP. Precipitated proteins were analyzed by quantitative immunoblotting. To reduce correlated errors, samples were loaded in a randomized order. Experiments were performed at least three times. One representative dataset is shown.

2.3 Figure S3

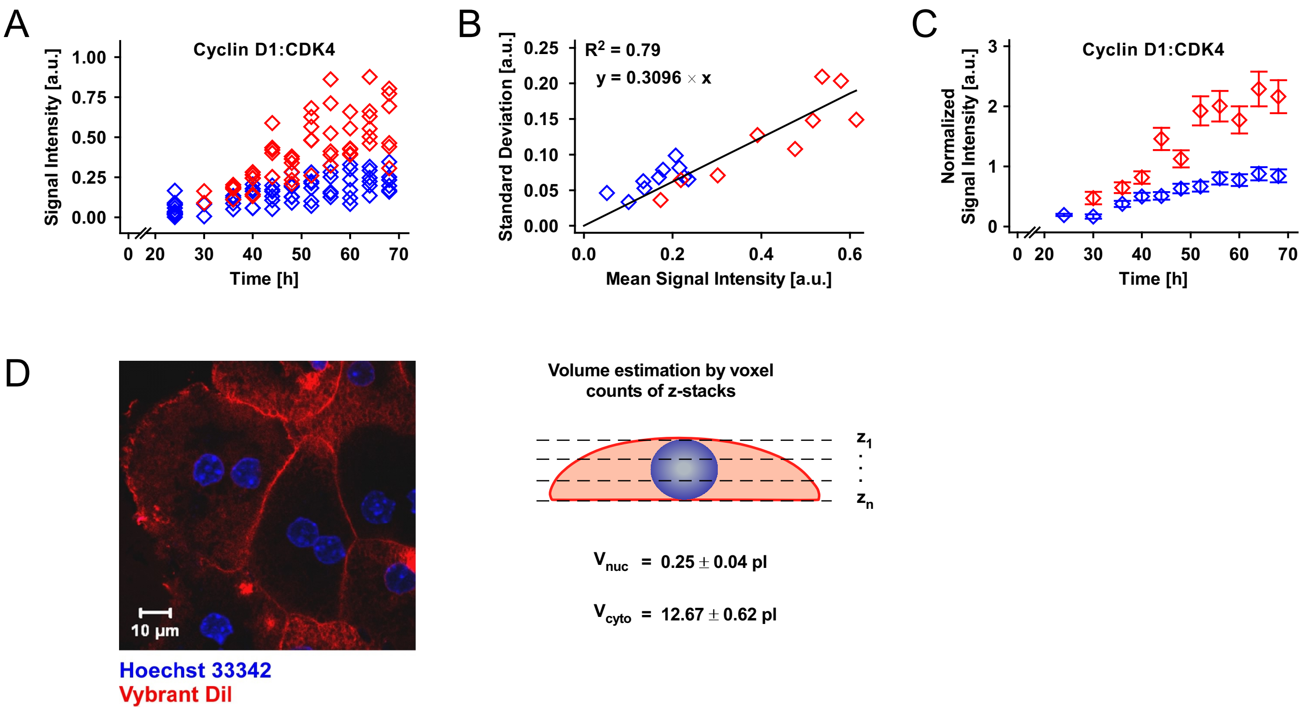

Figure S3. Data processing and error estimation of quantitative data was performed to provide merged data with equally distributed error bars and cytoplasmic and nuclear volumes were determined in primary mouse hepatocytes. (**A**) The process is illustrated for the protein species "Cyclin D1:CDK4", representing the amount of Cyclin D1 in complex with CDK4. Scaled immunoblotting data in biological replicates are shown. Primary mouse hepatocytes were stimulated with 40 ng/ml HGF (red) or remained untreated (blue). Cells were lysed at the indicated time points using total cell lysis buffer and proteins were subjected to immunoprecipitation using anti-CDK4 antibodies. Data was recorded by quantitative immunoblotting. Diamonds represent data obtained in six experiments that were scaled to each other and normalized to recombinant SBP-CDK4 as previously described [2]. (**B**) For estimation of the error bars, a constant relative error was assumed. For each data point the mean signal intensity, and, if more than two biological replicates were available, the standard deviation were calculated, and plotted against each other (open diamonds). *Solid line:* linear regression. *Slope:* relative error. (**C**) The processed data set with estimated absolute errors is shown. The mean signal intensity of each protein species was set to 1. *Diamonds:* normalized mean signal intensity, *error bars:* absolute estimated error corresponding to the product of relative estimated error determined in B, and normalized mean signal strength, divided by the square root of the number of replicates *a.u.:* arbitrary units. (**D**) Primary mouse hepatocytes were incubated with Vybrant Dil for plasma membrane and Hoechst 33342 for DNA staining. By confocal microscopy, z-stacks of three cells were recorded. After deconvolution, cell boundaries were manually marked for each stack and volumes were determined by voxel count. Nuclear volumes were estimated by approximating a spherical shape taking its diameter as basis of the calculation.

2.4 Figure S4

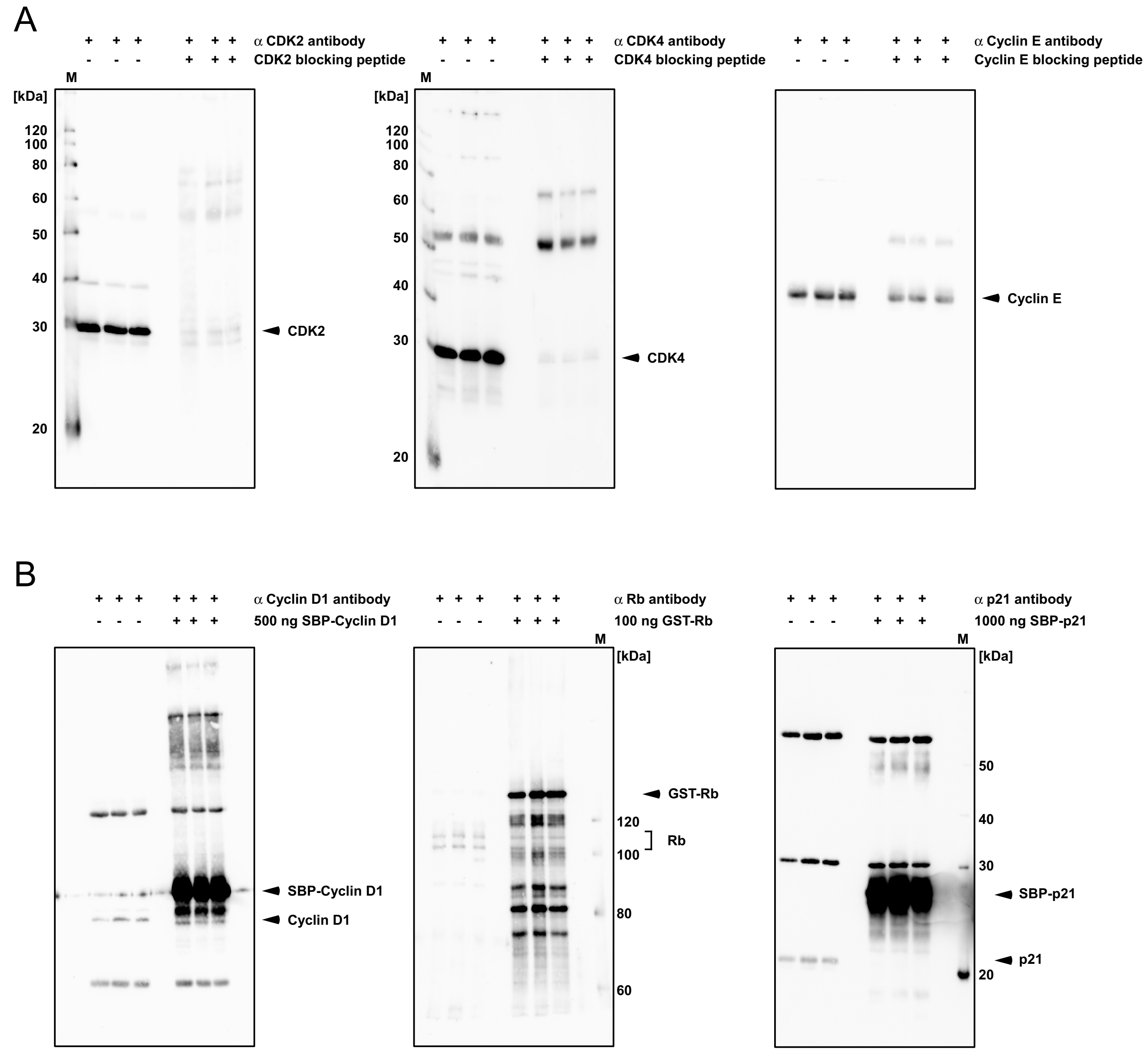

Figure S4. Control experiments demonstrating quality and specificity of the antibodies used. Primary mouse hepatocytes were stimulated with 40 ng/ml HGF for 48 h and immunoprecipitations were performed. (**A**) Blocking peptides for the antibodies directed against CDK2, CDK4 and Cyclin E were purchased from the manufacturer. The antibodies were pre-incubated with a 5-fold (by weight) excess of blocking peptide according to the manufacturer's instructions. Cellular proteins were subjected to immunoprecipitations (IP) with the indicated antibodies and subjected to quantitative immunoblotting. (**B**) The antibodies directed against Cyclin D1, Rb and p21 were pre-incubated with the indicated amounts of the recombinant proteins SBP-Cyclin D1, GST-Rb and SBP-p21, respectively. Cellular proteins were subjected to immunoprecipitations (IP) with the indicated antibodies and subjected to quantitative immunoblotting. M: protein weight marker.

2.5 Figure S5

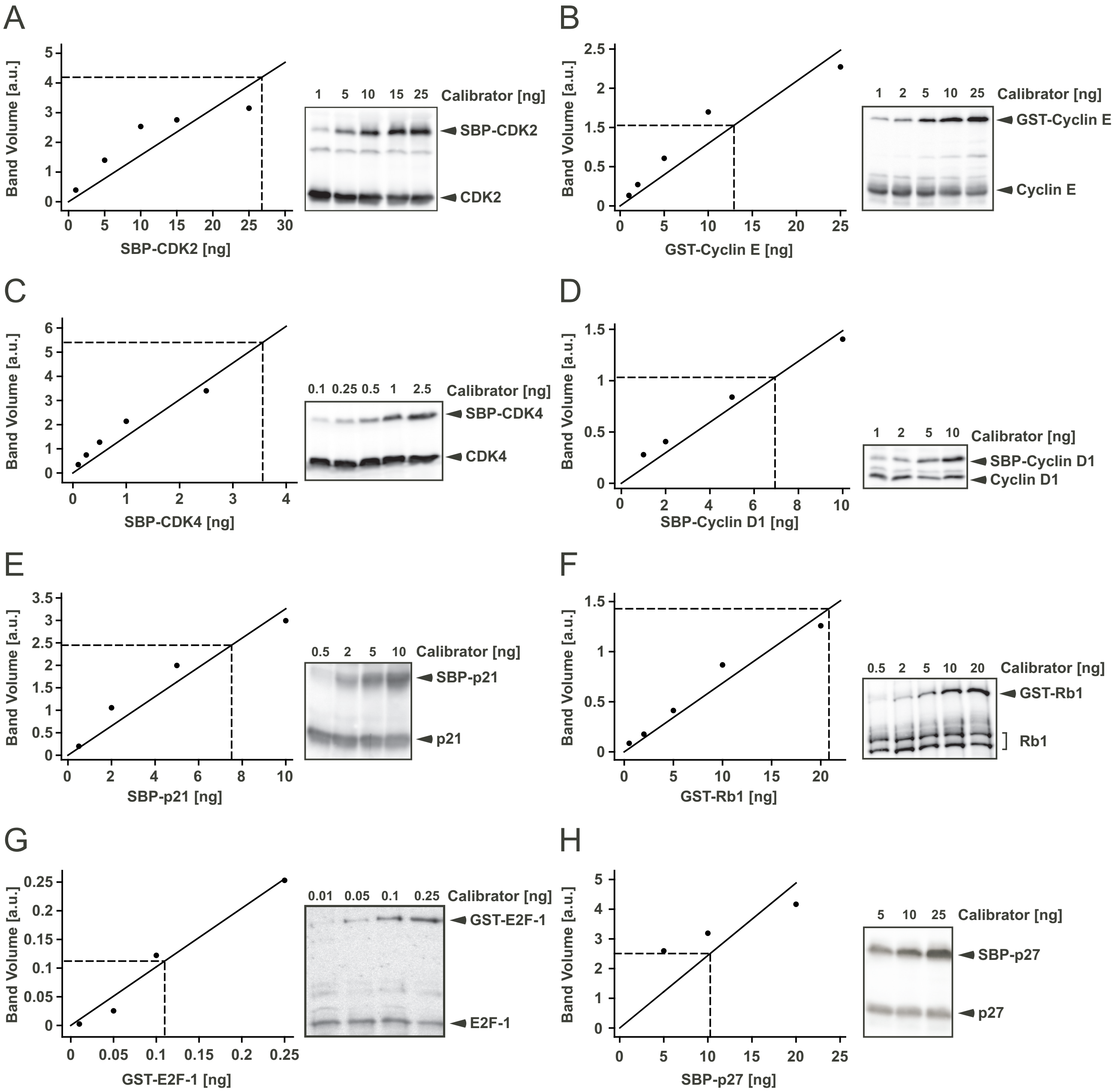

Figure S5. Recombinant calibrator proteins were employed to determine absolute amounts of cell cycle components. (**A–H**) Primary mouse hepatocytes were lysed at t = 56 h (32 h after stimulation with 40 ng/ml HGF, corresponding to the restriction point determined in Figure 1) using total cell lysis buffer and subjected to immunoprecipitation. Prior to immunoprecipitation, a dilution series of recombinant calibrator protein was added to the lysates. Signals were recorded by quantitative immunoblotting. Standard curves were calculated based on the signal of the calibrator proteins. Filled circles represent calibrator protein signals; solid lines indicate the linear regressions. Since a background correction was performed during quantification of the immunoblots, linear regressions without offsets were performed. Dashed lines mark the average amount of the endogenous proteins. *a.u.:* arbitrary units.

2.6 Figure S6

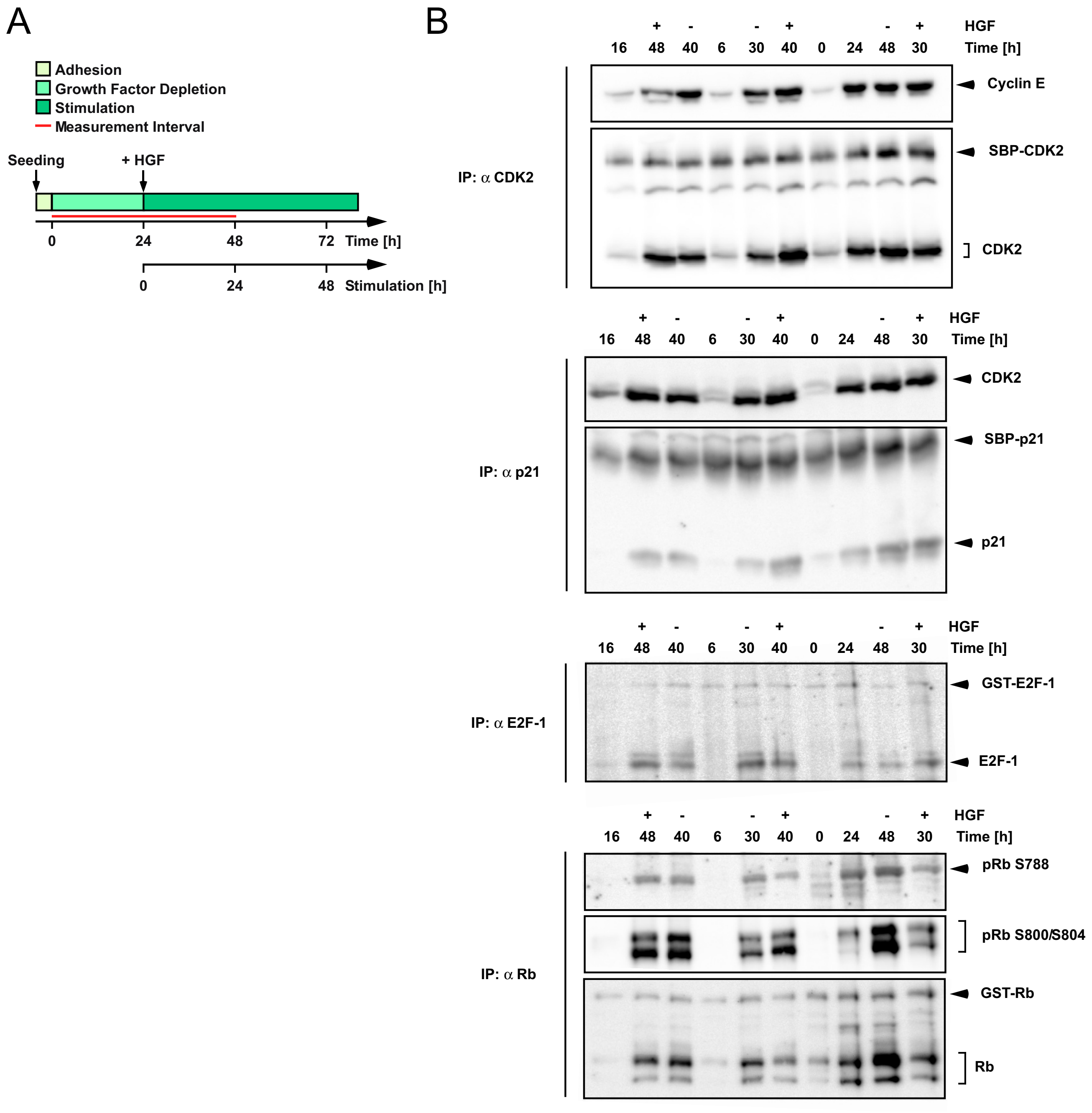

Figure S6. Quantitative immunoblot data of primary mouse hepatocytes in the growth factor depletion phase. (**A**) Primary mouse hepatocytes were isolated by liver perfusion and cultivated according to the depicted scheme. After 24 h, hepatocytes were stimulated with 40 ng/ml HGF or remained untreated. Cells were lysed at different time points within the measurement interval indicated by the red solid line, corresponding to the growth factor depletion phase as well as the initial stimulation phase to allow comparison of this data set with the data set depicted in Figure S2. (**B**) Time-resolved investigation of G1/S transition components by quantitative immunoblotting was performed. Primary mouse hepatocytes were growth factor depleted for 24 h, stimulated with 40 ng/ml HGF or remained untreated, and cells were lysed using total cellular lysis buffer at the indicated time points. Total proteins were subjected to immunoprecipitations (IP) with the indicated antibodies. To allow for data normalization, recombinant GST- or SBP-tagged calibrator proteins were added to the lysates before performing the IP. Precipitated proteins were analyzed by quantitative immunoblotting. To reduce correlated errors, samples were loaded in a randomized order. Experiments were performed at least three times. One representative data set is shown. Data was merged with the data depicted in Figure S2 as described in Figure S3.

2.7 Figure S7

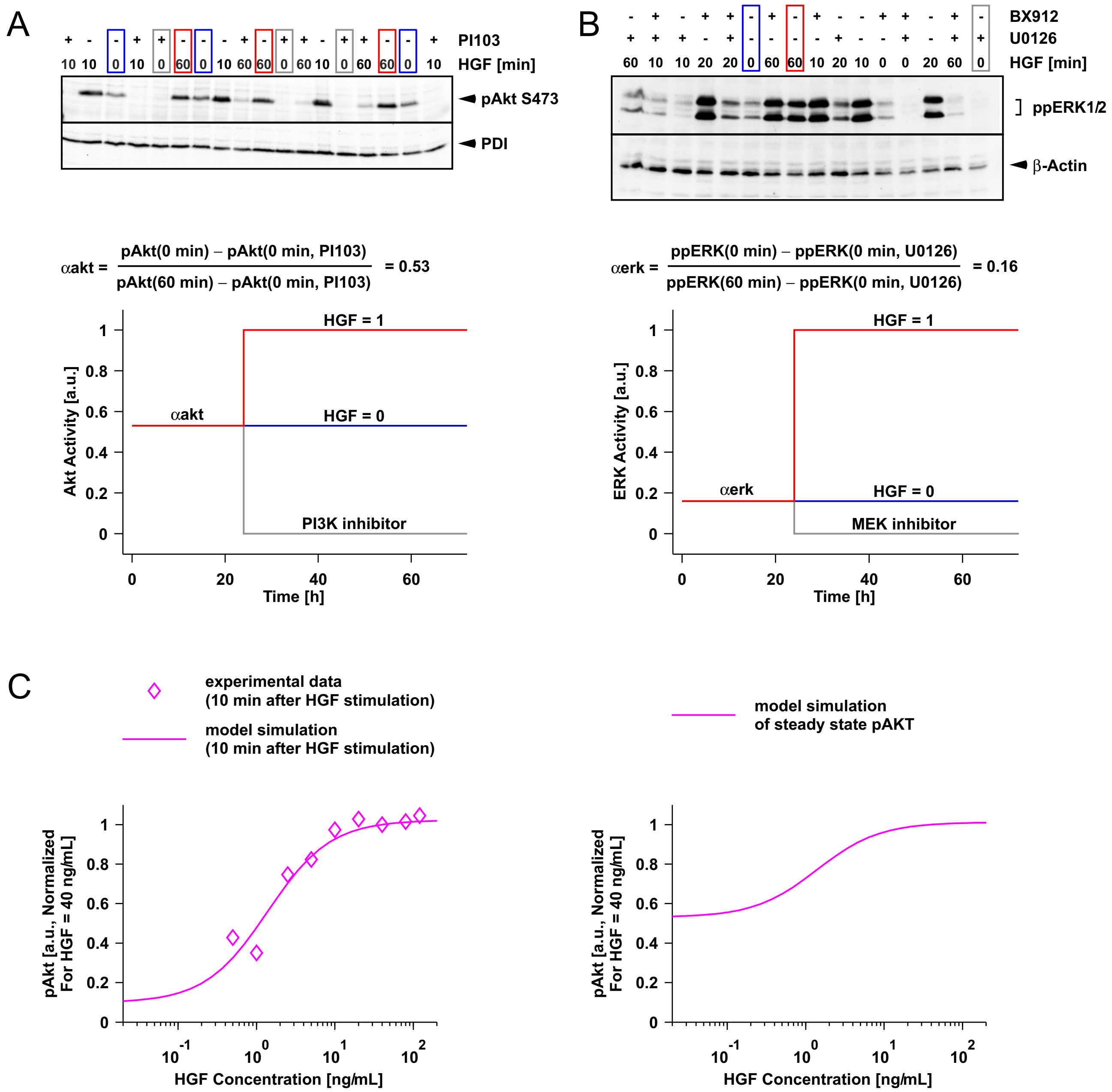

Figure S7. The basal activities of Akt (**A**) and ERK (**B**) were determined in unstimulated primary mouse hepatocytes. Primary mouse hepatocytes were isolated by liver perfusion and growth factors were depleted. After 24 h, cells were stimulated with 40 ng/ml HGF, pretreated for 30 min with PI3K inhibitor (5 μM PI103), MEK inhibitor (20 μM U0126), PDK1 inhibitor (20 μM BX912), or left untreated. Phosphorylation of Akt and ERK were determined by quantitative immunoblotting. Boxes indicate samples that were used in this analysis. Basal activities of ERK and Akt (α_{erk} and α_{akt}, respectively) were calculated as the phosphorylation ratio of HGF-stimulated to untreated cells, subtracting the phosphorylation levels of inhibitor-treated cells as background. (**C**). Primary mouse hepatocytes were isolated by liver perfusion and growth factors were depleted. After 24 h, cells were stimulated with increasing doses of HGF and cells were lysed after 10 min. Dose-dependent phosphorylation levels of Akt were determined in a bead-based multiplexing assay. The data was employed to determine inflection point and steepness of dose-dependency at 10 min after stimulation (*left panel*). To determine the dose response curve for steady state levels of pAkt, the basal activity determined in (A) was additionally taken into account (*right panel*).

2.8 Figure S8

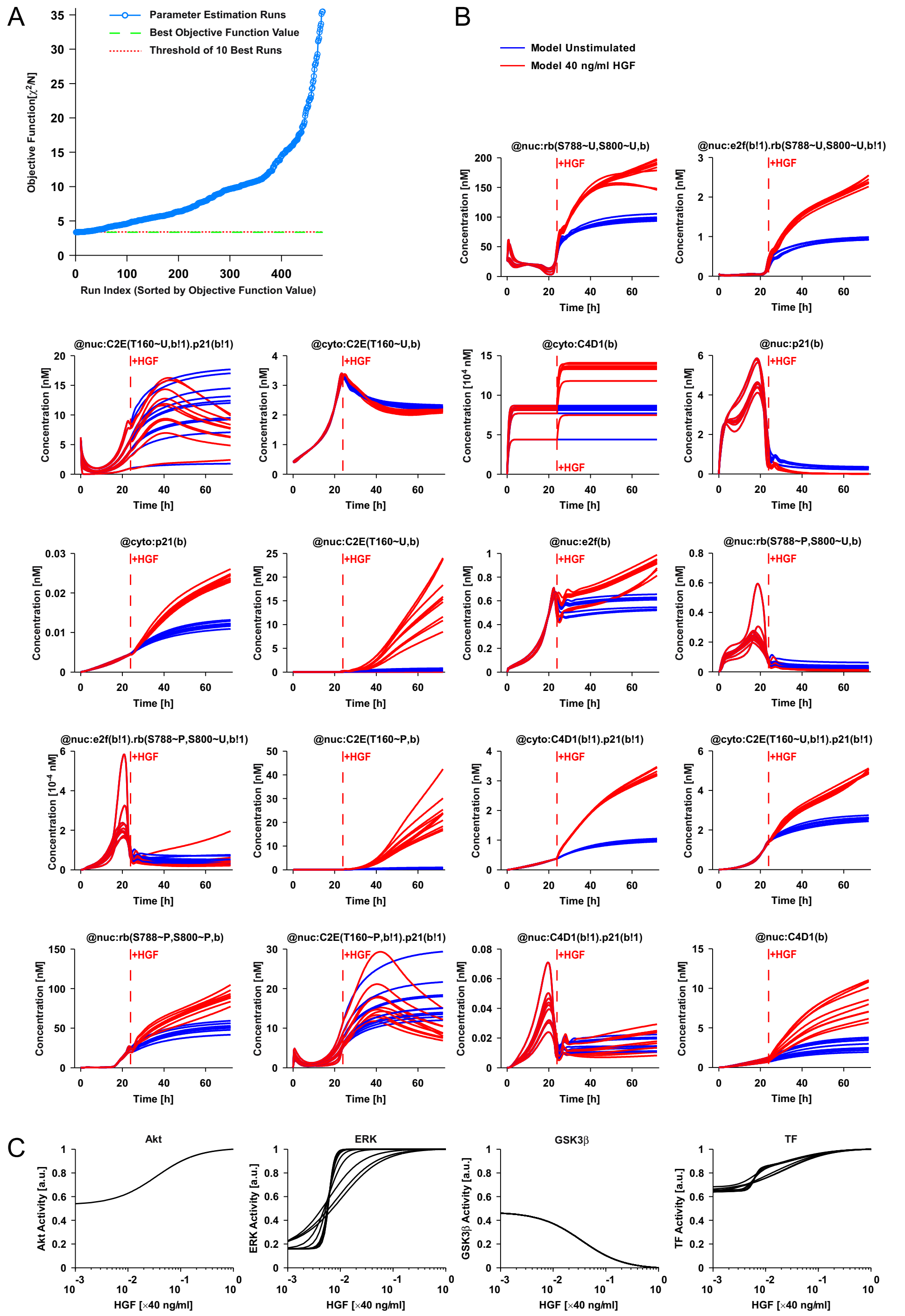

Figure S8. Calibration of the mathematical model based on the experimental data depicted in Figure 4. (**A**) In total, 500 parameter estimation runs were performed. The runs were sorted by the corresponding value of the objective function. A lower value of the objective function indicates a better goodness-of-fit. The 10 best parameter estimation runs with a similar value of the objective function that were further analyzed are indicated. (**B**) The model variables corresponding to the 10 best parameter estimations are displayed over time. See Table S5 for the equations mapping these variables to the measured species (Figure 4). The dynamics between 0 and 24 h (growth factor depletion phase) is the same for both the unstimulated and the 40 ng/ml HGF-stimulated condition. (**C**) The dose-dependency of the input variables (Figure 3A) is shown. The HGF-dependent activity of Akt and consequently GSK3β was experimentally determined in Figure S6. The HGF-dependent activity of ERK and consequently the activity of the transcription factors (TF) were estimated by the model based on the experimental data (Figure 4) and the trajectories corresponding to the 10 best parameter estimation runs are displayed.

2.9 Figure S9

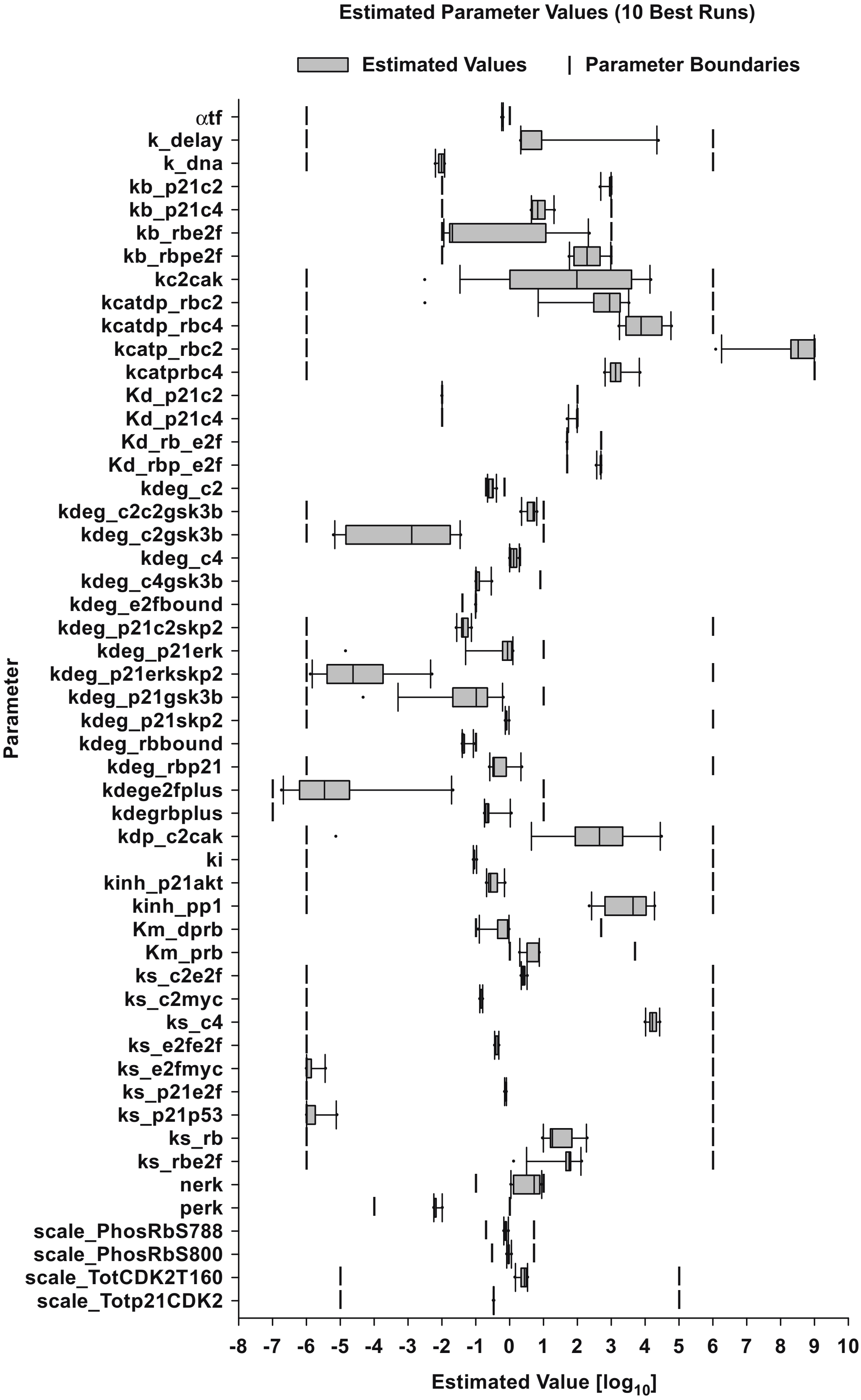

Figure S9. The parameter values of the 10 best parameter estimation runs are shown as box plot. Parameter estimation was performed over up to 12 orders of magnitude, parameter boundaries are displayed as vertical lines. The box plot displays the median, 10th, 25th, 75th and 90th percentiles as horizontal boxes with error bars. Outliers (5th and 95th percentiles) are shown as dots.

2.10 Figure S10

Parameter	Run 1	Run 2	Run 3	Run 4	Run 5	Run 6	Run 7	Run 8	Run 9	Run 10
αtf	6.0129×10^{-1}	6.1743×10^{-1}	6.2338×10^{-1}	6.5167×10^{-1}	6.2204×10^{-1}	6.0926×10^{-1}	5.8762×10^{-1}	6.0584×10^{-1}	6.1202×10^{-1}	6.1147×10^{-1}
k_delay	2.3666×10^{1}	2.4968×10^{4}	2.2623×10^{0}	2.1640×10^{0}	2.2044×10^{0}	2.2398×10^{0}	3.9069×10^{0}	2.1612×10^{0}	2.1973×10^{0}	2.1302×10^{0}
k_dna	9.4979×10^{-3}	9.9844×10^{-3}	8.2458×10^{-3}	1.1337×10^{-2}	1.1989×10^{-2}	7.1384×10^{-3}	6.3247×10^{-3}	9.1316×10^{-3}	1.1656×10^{-2}	1.0023×10^{-2}
kb_p21c2	9.9794×10^{2}	7.0358×10^{2}	9.6376×10^{2}	9.8576×10^{2}	9.9898×10^{2}	9.9723×10^{2}	4.7251×10^{2}	9.6974×10^{2}	9.9893×10^{2}	9.9991×10^{2}
kb_p21c4	1.4308×10^{1}	2.1126×10^{1}	7.0893×10^{0}	4.3776×10^{0}	4.7318×10^{0}	4.5618×10^{0}	1.0055×10^{1}	4.9988×10^{0}	6.4396×10^{0}	8.4967×10^{0}
kb_rbe2f	2.2998×10^{2}	4.4542×10^{1}	1.8303×10^{-2}	1.0747×10^{-2}	1.6228×10^{-2}	2.3040×10^{-2}	7.7122×10^{-1}	2.0183×10^{-2}	2.0166×10^{-2}	1.7071×10^{-2}
kb_rbpe2f	1.8222×10^{2}	8.6884×10^{2}	5.7604×10^{1}	2.3514×10^{2}	3.4038×10^{2}	2.0430×10^{2}	9.8968×10^{2}	1.4919×10^{2}	8.3912×10^{1}	6.8320×10^{1}
kc2cak	3.1699×10^{-1}	3.1231×10^{-3}	1.3044×10^{3}	6.1201×10^{1}	3.9118×10^{1}	3.5153×10^{3}	1.4936×10^{4}	5.5286×10^{3}	1.2704×10^{0}	1.3291×10^{2}
kcatdp_rbc2	3.1384×10^{-3}	7.0232×10^{1}	3.3645×10^{3}	3.8625×10^{2}	2.6681×10^{3}	8.5065×10^{2}	9.5989×10^{2}	1.3881×10^{3}	1.5579×10^{3}	5.1081×10^{2}
kcatdp_rbc4	2.8920×10^{3}	1.7072×10^{3}	6.0536×10^{4}	3.2500×10^{3}	1.9260×10^{4}	1.0194×10^{4}	5.0348×10^{3}	3.4331×10^{4}	3.0373×10^{4}	2.2384×10^{3}
kcatp_rbc2	7.1423×10^{6}	1.2292×10^{6}	3.2633×10^{8}	9.9912×10^{8}	6.2442×10^{8}	3.0323×10^{8}	3.3941×10^{8}	9.9722×10^{8}	2.6818×10^{8}	9.9976×10^{8}
kcatprbc4	2.7978×10^{3}	1.5896×10^{3}	1.3345×10^{3}	6.4418×10^{2}	8.4060×10^{2}	1.0086×10^{3}	7.1830×10^{3}	1.0224×10^{3}	1.3606×10^{3}	1.6330×10^{3}
Kd_p21c2	1.0002×10^{-2}	1.0002×10^{-2}	1.0001×10^{-2}	1.0000×10^{-2}	1.0009×10^{-2}	1.0000×10^{-2}	1.0002×10^{-2}	1.0003×10^{-2}	1.0000×10^{-2}	1.0001×10^{-2}
Kd_p21c4	9.9997×10^{1}	5.0353×10^{1}	9.9930×10^{1}	9.6380×10^{1}	9.9949×10^{1}	9.9972×10^{1}	9.9986×10^{1}	9.5697×10^{1}	9.9962×10^{1}	9.9793×10^{1}
Kd_rb_e2f	5.0003×10^{1}	5.0006×10^{1}	5.0000×10^{1}	5.0001×10^{1}	5.0000×10^{1}	5.0000×10^{1}	5.0009×10^{1}	5.0001×10^{1}	5.0031×10^{1}	5.0000×10^{1}
Kd_rbp_e2f	4.8148×10^{2}	4.9889×10^{2}	4.9960×10^{2}	3.6362×10^{2}	4.9967×10^{2}	4.9995×10^{2}	4.9992×10^{2}	4.6192×10^{2}	4.9976×10^{2}	4.8451×10^{2}
kdeg_c2	2.2575×10^{-1}	2.2406×10^{-1}	3.1431×10^{-1}	4.1771×10^{-1}	3.5394×10^{-1}	2.8914×10^{-1}	2.4546×10^{-1}	3.2093×10^{-1}	3.1205×10^{-1}	3.1076×10^{-1}
kdeg_c2c2gsk3b	5.5884×10^{0}	6.4514×10^{0}	3.8904×10^{0}	5.1969×10^{0}	5.3775×10^{0}	3.3355×10^{0}	2.1403×10^{0}	3.3882×10^{0}	4.8622×10^{0}	5.0967×10^{0}
kdeg_c2gsk3b	1.5509×10^{-5}	6.3049×10^{-6}	6.2407×10^{-5}	2.2087×10^{-3}	1.1955×10^{-5}	5.5991×10^{-3}	3.6104×10^{-2}	2.0962×10^{-2}	1.6418×10^{-2}	3.6148×10^{-4}
kdeg_c4	1.0143×10^{0}	1.3491×10^{0}	1.6173×10^{0}	1.1024×10^{0}	1.1381×10^{0}	1.0000×10^{0}	1.6817×10^{0}	1.2989×10^{0}	1.5854×10^{0}	1.9704×10^{0}
kdeg_c4gsk3b	1.0764×10^{-1}	3.0218×10^{-1}	1.0720×10^{-1}	1.0287×10^{-1}	1.1616×10^{-1}	1.0157×10^{-1}	1.0223×10^{-1}	1.1937×10^{-1}	1.0140×10^{-1}	1.5108×10^{-1}
kdeg_e2fbound	9.9995×10^{-2}	9.9987×10^{-2}	9.9998×10^{-2}	9.9999×10^{-2}	1.0000×10^{-1}	9.9988×10^{-2}	9.9982×10^{-2}	9.9991×10^{-2}	1.0000×10^{-1}	9.9999×10^{-2}
kdeg_p21c2skp2	4.0108×10^{-2}	4.1230×10^{-2}	4.1233×10^{-2}	7.5802×10^{-2}	6.3938×10^{-2}	3.4633×10^{-2}	2.6384×10^{-2}	4.4721×10^{-2}	5.8078×10^{-2}	5.0420×10^{-2}
kdeg_p21erk	7.3649×10^{-1}	6.6349×10^{-1}	5.0382×10^{-1}	1.4219×10^{-5}	1.1288×10^{0}	8.8758×10^{-1}	8.8288×10^{-1}	1.2592×10^{0}	8.6265×10^{-1}	1.2487×10^{0}
kdeg_p21erkskp2	2.8298×10^{-4}	2.9642×10^{-6}	8.0529×10^{-6}	5.1135×10^{-3}	2.7195×10^{-5}	1.9874×10^{-5}	1.3383×10^{-4}	1.3046×10^{-6}	1.4678×10^{-4}	4.4091×10^{-6}
kdeg_p21gsk3b	4.6401×10^{-3}	2.6257×10^{-2}	3.0484×10^{-1}	6.5852×10^{-1}	1.6114×10^{-1}	1.3525×10^{-1}	4.7246×10^{-5}	7.2057×10^{-2}	1.9229×10^{-1}	7.2931×10^{-2}
kdeg_p21skp2	7.5057×10^{-1}	7.7405×10^{-1}	7.9185×10^{-1}	9.6967×10^{-1}	8.6829×10^{-1}	7.5521×10^{-1}	7.5356×10^{-1}	8.1217×10^{-1}	7.9138×10^{-1}	7.9323×10^{-1}
kdeg_rbbound	8.8996×10^{-2}	4.0850×10^{-2}	4.0006×10^{-2}	4.0005×10^{-2}	4.0004×10^{-2}	4.0011×10^{-2}	4.9655×10^{-2}	4.4917×10^{-2}	4.0011×10^{-2}	4.0026×10^{-2}
kdeg_rbp21	8.6357×10^{-1}	7.7180×10^{-1}	3.3056×10^{-1}	2.5666×10^{-1}	3.0240×10^{-1}	3.7884×10^{-1}	2.3201×10^{0}	3.4863×10^{-1}	3.5645×10^{-1}	3.3497×10^{-1}
kdege2fplus	4.1815×10^{-5}	2.1290×10^{-2}	4.2354×10^{-6}	2.2032×10^{-6}	6.8617×10^{-7}	1.8304×10^{-7}	4.0658×10^{-6}	4.1744×10^{-7}	1.0786×10^{-5}	2.7544×10^{-6}
kdegrbplus	2.5776×10^{-1}	2.3652×10^{-1}	1.9880×10^{-1}	1.8129×10^{-1}	1.9343×10^{-1}	2.2561×10^{-1}	1.1337×10^{0}	2.2713×10^{-1}	2.2501×10^{-1}	1.9835×10^{-1}
kdp_c2cak	1.0128×10^{2}	4.4365×10^{1}	1.1054×10^{3}	4.7165×10^{2}	4.3057×10^{2}	1.3781×10^{3}	7.3775×10^{-6}	4.6321×10^{3}	3.0927×10^{4}	3.7168×10^{2}
ki	9.1833×10^{-2}	1.0749×10^{-1}	8.6587×10^{-2}	9.1772×10^{-2}	9.1193×10^{-2}	8.4878×10^{-2}	8.9329×10^{-2}	8.8692×10^{-2}	8.8724×10^{-2}	8.8278×10^{-2}
kinh_p21akt	4.3966×10^{-1}	7.3913×10^{-1}	2.3524×10^{-1}	2.0795×10^{-1}	2.6761×10^{-1}	2.4544×10^{-1}	4.3889×10^{-1}	2.7792×10^{-1}	2.7251×10^{-1}	2.8115×10^{-1}
kinh_pp1	1.6635×10^{4}	1.9322×10^{4}	8.7666×10^{3}	5.9322×10^{2}	6.2844×10^{3}	1.6107×10^{3}	2.2635×10^{2}	2.5980×10^{3}	6.4902×10^{3}	6.7935×10^{2}
Km_dprb	1.1899×10^{-1}	2.1162×10^{-1}	9.2137×10^{-1}	8.8991×10^{-1}	9.0905×10^{-1}	8.5960×10^{-1}	5.2821×10^{-1}	9.1248×10^{-1}	9.7241×10^{-1}	7.2805×10^{-1}
Km_prb	2.0346×10^{0}	1.9940×10^{0}	7.0406×10^{0}	7.3383×10^{0}	7.0852×10^{0}	7.4466×10^{0}	3.7131×10^{0}	7.5569×10^{0}	7.3610×10^{0}	7.5024×10^{0}
ks_c2e2f	2.1994×10^{0}	2.2279×10^{0}	2.6346×10^{0}	3.3421×10^{0}	2.9408×10^{0}	2.4915×10^{0}	2.4355×10^{0}	2.7615×10^{0}	2.6728×10^{0}	2.6236×10^{0}
ks_c2myc	1.5751×10^{-1}	1.5543×10^{-1}	1.3524×10^{-1}	1.5153×10^{-1}	1.4085×10^{-1}	1.3205×10^{-1}	1.6104×10^{-1}	1.3907×10^{-1}	1.3637×10^{-1}	1.3169×10^{-1}
ks_c4	1.4299×10^{4}	1.0075×10^{4}	2.1481×10^{4}	1.2997×10^{4}	1.5584×10^{4}	1.4047×10^{4}	2.2514×10^{4}	1.7448×10^{4}	2.1391×10^{4}	2.7474×10^{4}
ks_e2fe2f	4.5960×10^{-1}	4.8563×10^{-1}	3.8425×10^{-1}	3.6193×10^{-1}	3.8310×10^{-1}	3.9595×10^{-1}	4.5589×10^{-1}	3.9444×10^{-1}	3.9133×10^{-1}	3.8961×10^{-1}
ks_e2fmyc	2.4917×10^{-6}	1.0003×10^{-6}	1.0000×10^{-6}	1.0001×10^{-6}	1.0001×10^{-6}	1.0000×10^{-6}	1.0189×10^{-6}	1.0020×10^{-6}	3.6821×10^{-6}	1.0047×10^{-6}
ks_p21e2f	8.1162×10^{-1}	8.0438×10^{-1}	7.1692×10^{-1}	7.2836×10^{-1}	7.3308×10^{-1}	7.2052×10^{-1}	8.0396×10^{-1}	7.3552×10^{-1}	7.3076×10^{-1}	7.2142×10^{-1}
ks_p21p53	3.8414×10^{-6}	1.1437×10^{-6}	1.0015×10^{-6}	1.0023×10^{-6}	1.0005×10^{-6}	1.0000×10^{-6}	1.0057×10^{-6}	1.0050×10^{-6}	7.9631×10^{-6}	1.0316×10^{-6}
ks_rb	7.2525×10^{1}	6.7690×10^{1}	1.7692×10^{1}	9.4044×10^{0}	1.4346×10^{1}	2.1840×10^{1}	1.9727×10^{2}	1.7970×10^{1}	1.8877×10^{1}	1.6721×10^{1}
ks_rbe2f	2.0013×10^{1}	1.3202×10^{0}	5.4867×10^{1}	5.6380×10^{1}	5.7664×10^{1}	5.9132×10^{1}	1.3553×10^{2}	6.6127×10^{1}	6.2507×10^{1}	5.6967×10^{1}
nerk	1.1466×10^{0}	1.0957×10^{0}	6.3619×10^{0}	2.7433×10^{0}	8.8778×10^{0}	6.8264×10^{0}	1.3640×10^{0}	4.3043×10^{0}	7.5031×10^{0}	8.7407×10^{0}
perk	8.5731×10^{-3}	1.0322×10^{-2}	6.0040×10^{-3}	5.7202×10^{-3}	6.0650×10^{-3}	5.9900×10^{-3}	5.9645×10^{-3}	5.7766×10^{-3}	6.0091×10^{-3}	6.0714×10^{-3}
scale_PhosRbS788	6.7391×10^{-1}	6.9922×10^{-1}	7.7640×10^{-1}	8.4084×10^{-1}	7.8553×10^{-1}	7.5445×10^{-1}	9.3471×10^{-1}	7.9413×10^{-1}	7.7595×10^{-1}	7.7667×10^{-1}
scale_PhosRbS800	8.2377×10^{-1}	8.7672×10^{-1}	9.4556×10^{-1}	1.0226×10^{0}	9.5754×10^{-1}	9.1868×10^{-1}	1.1454×10^{0}	9.6135×10^{-1}	9.4706×10^{-1}	9.4674×10^{-1}
scale_TotCDK2T160	2.7284×10^{0}	2.9249×10^{0}	2.2696×10^{0}	3.4268×10^{0}	3.2556×10^{0}	1.9366×10^{0}	1.4494×10^{0}	2.3422×10^{0}	3.0931×10^{0}	2.7806×10^{0}
scale_Totp21CDK2	3.3979×10^{-1}	3.4656×10^{-1}	3.3724×10^{-1}	3.3120×10^{-1}	3.4069×10^{-1}	3.4150×10^{-1}	3.3642×10^{-1}	3.4114×10^{-1}	3.3788×10^{-1}	3.4269×10^{-1}

Figure S10. Estimated parameter values for the 10 best parameter estimation run are shown. Parameter estimation runs are sorted by increasing objective function value (decreasing goodness-of-fit).

2.11 Figure S11

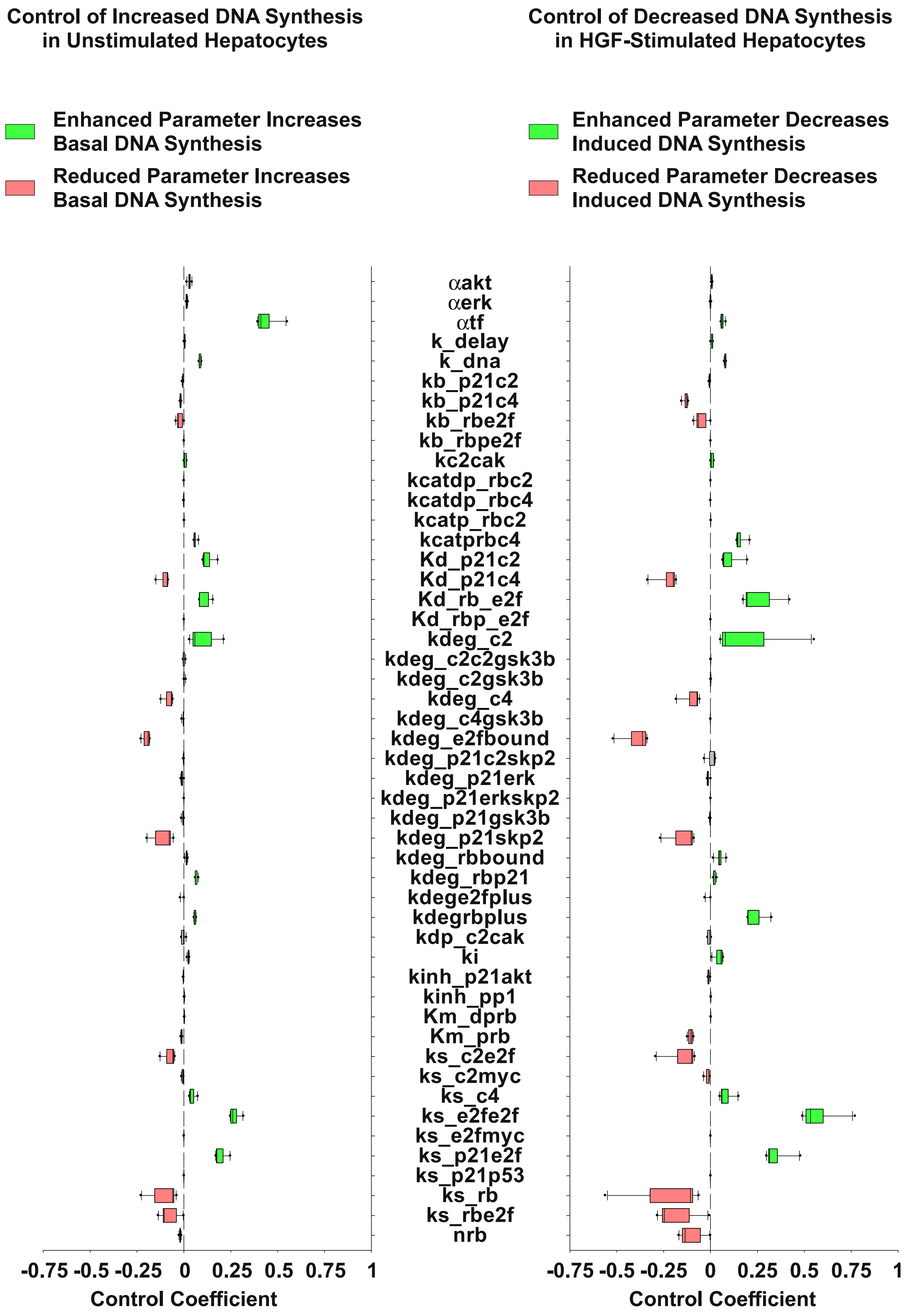

Figure S11. Control coefficients of the model parameters with respect to basal DNA synthesis (left side of plot) and with respect to induced DNA synthesis (right side of plot) were calculated for the 10 best parameter estimations. Control coefficients are displayed with the median, 10th, 25th, 75th and 90th percentiles as horizontal boxes with error bars. Outliers (5th and 95th percentiles) are shown as dots.

2.12 Figure S12

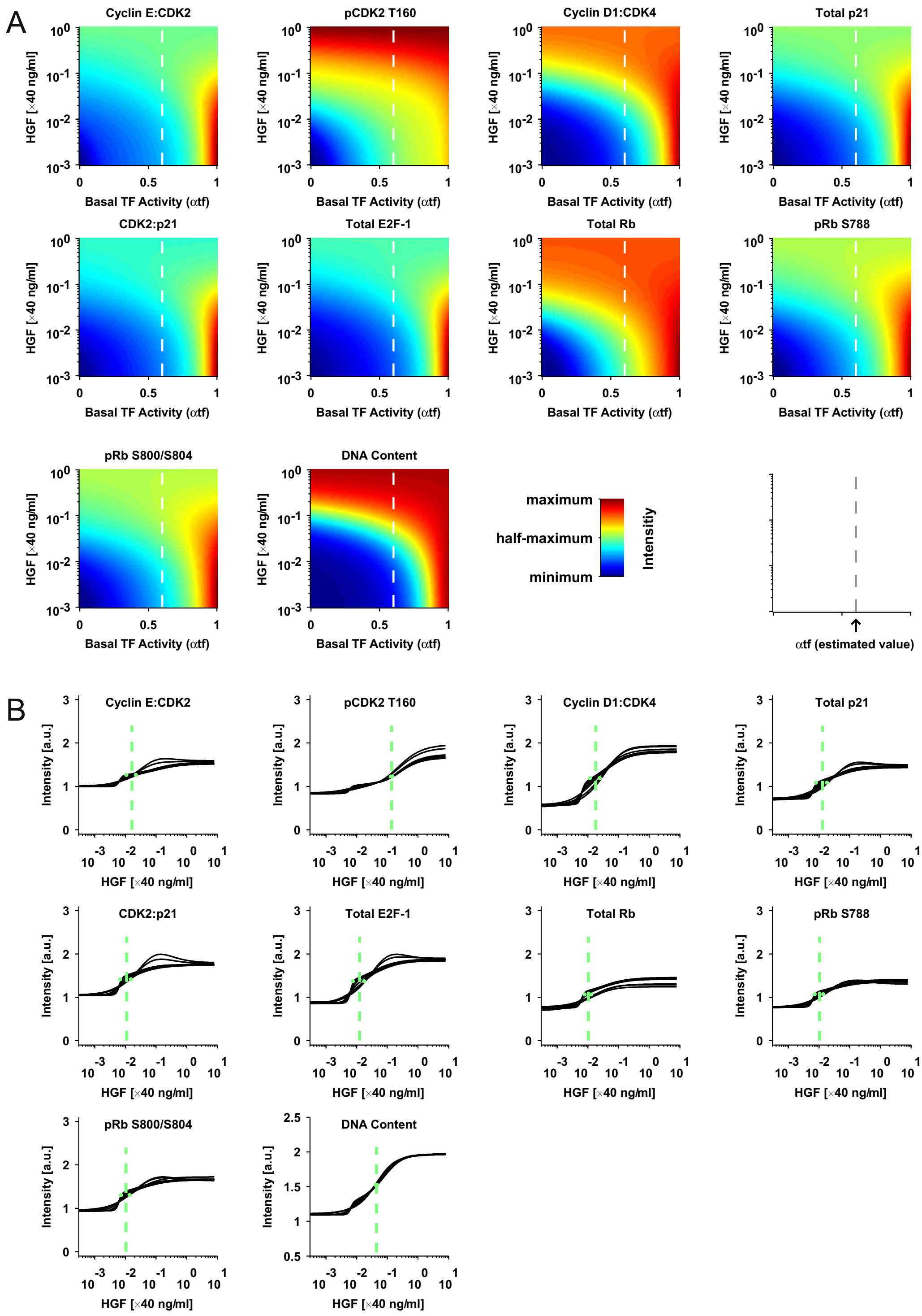

Figure S12. Dependency of G1/S transition components on HGF concentration. (**A**) The intensity of the cell cycle proteins and of the DNA content at 72 h was calculated as a function of HGF and basal TF activity. Intensity is depicted with colors from blue (minimum intensity) to red (maximum intensity) for the cell cycle proteins. For DNA content, the range is displayed between one (no change) and two (doubling of the DNA). Basal activity of TF as estimated in Figure S7C is indicated with dashed white lines. Analysis is based on the best parameter estimation round. (**B**) For each cell cycle protein and for DNA content, the model predicted HGF-dependency at 72 h was calculated based on the 10 best parameter estimations. The average HGF concentration inducing half-maximum intensity was calculated and indicated with vertical dashed green lines. The uncertainty is characterized with green dots at half-maximum intensity.

2.13 Figure S13

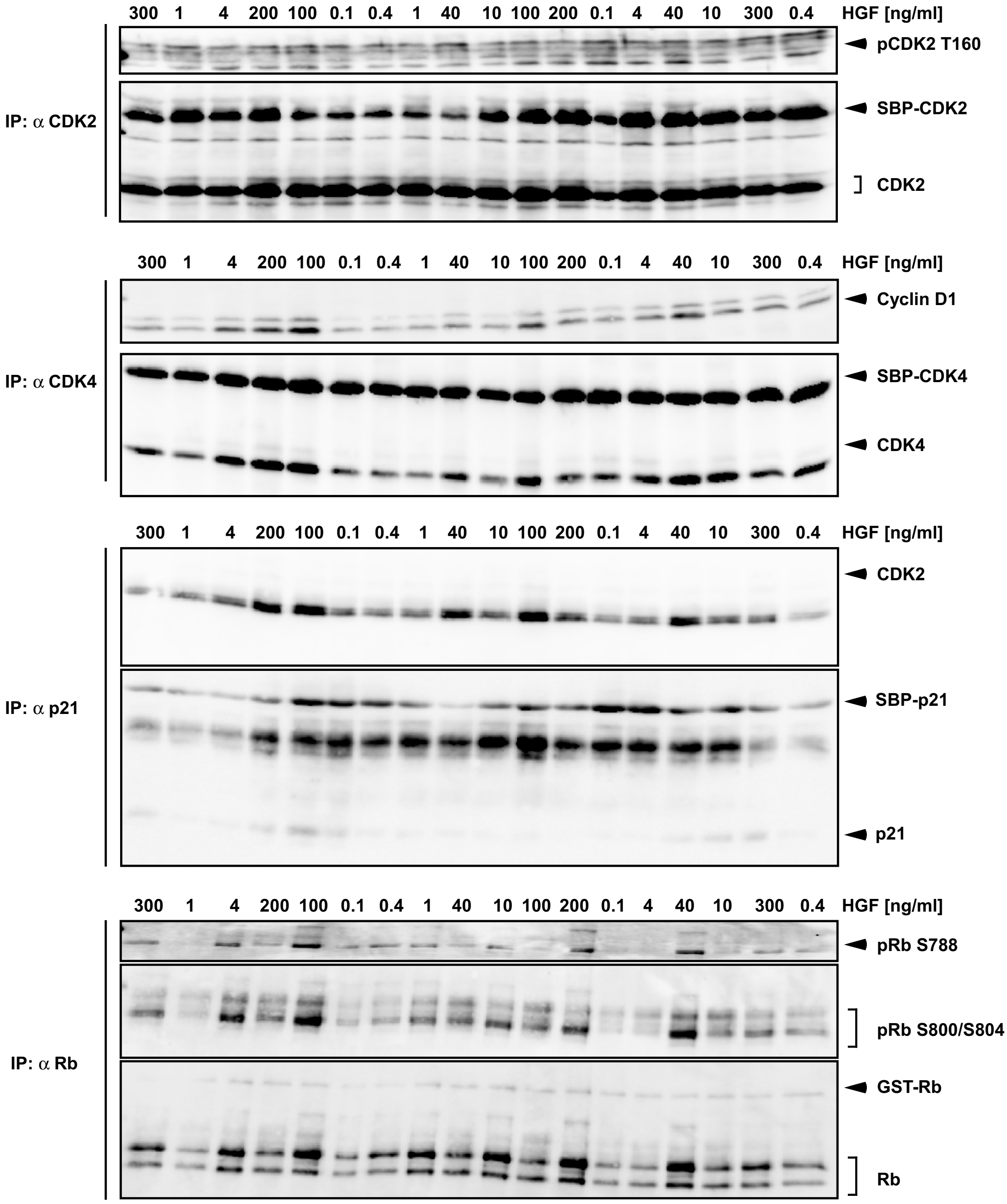

Figure S13. Quantitative immunoblot data of primary mouse hepatocytes stimulated with different concentrations of HGF for model validation. (**A**) Primary mouse hepatocytes were isolated by liver perfusion and cultivated as described above. After 24 h, hepatocytes were stimulated with with indicated doses of HGF. Cells were lysed after 48 h using total cellular lysis buffer. Total proteins were subjected to immunoprecipitations (IP) with the indicated antibodies and precipitated proteins were analyzed by quantitative immunoblotting. To reduce correlated errors, samples were loaded in a randomized order. Experiments were performed at least three times. One representative data set is shown. Data was merged as described in Figure S3 and the EC_{50} was calculated as the inflection point of a four-parameter Hill function. A nonlinear regression of signal intensity (y) as a function of HGF concentration (x) was performed.

2.14 Figure S14

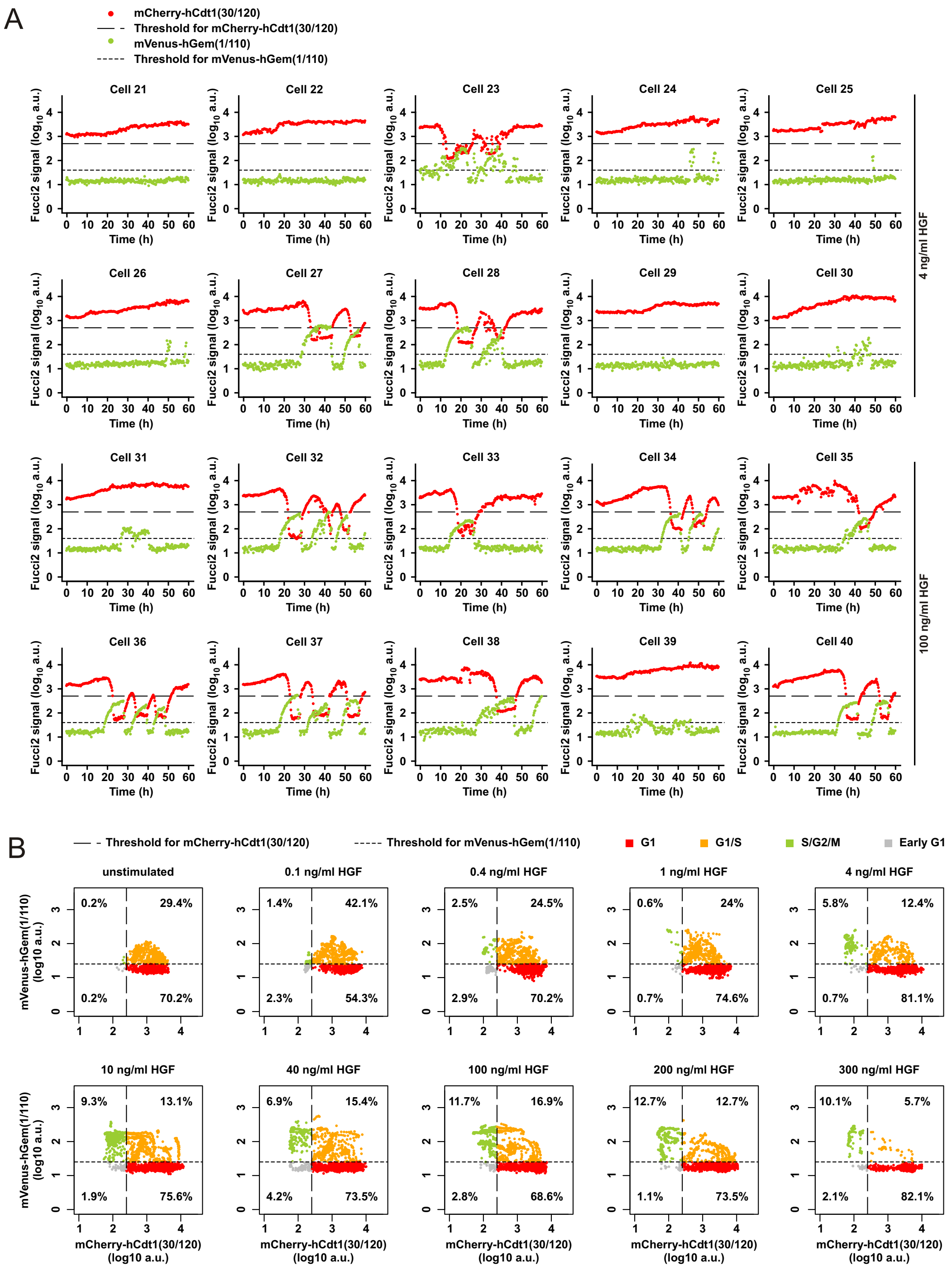

Figure S14. Single cells tracks and dose response analysis of Fucci2 primary mouse hepatocytes. (**A**) Primary mouse hepatocytes of C57BL/6N-Fucci2 animals were transduced with Histone2B-mCerulean by an adeno-associated virus-based protocol to enable tracking of the cells. Live cell microscopy was performed of the Fucci2 primary mouse hepatocytes stimulated with 4 ng/ml HGF or 100 ng/ml HGF (sampling rate of 15 min for up to 60 h) and 20 cells were manually tracked. Thresholds were defined for the Fucci2 signals. Figure 8A shows the cell cycle phases of this data set. (**B**) Primary mouse hepatocytes of C57BL/6N-Fucci2 animals were transduced with Histone2B-mCerulean by an adeno-associated virus-based protocol to enable segmentation of the cell nuclei. Live cell microscopy was performed of the Fucci2 primary mouse hepatocytes stimulated with indicated concentrations of HGF (sampling rate of 15 min for up to 60 h). Fucci2 signals of cell nuclei between 42 and 54 h after stimulation were plotted and percentage of nuclei in the respective cell cycle phases were calculated based on thresholds for the Fucci2 signals using the unstimulated condition as a reference. This data set represents the biological replicate of Figure 8C.

3 Description of the model species and regulatory mechanisms

3.1 Cyclins and cyclin-dependent kinases

Because the concentration of total CDK4 remained constant in primary mouse hepatocytes (Fig 2A–2E), the increase in the concentration of the Cyclin D1:CDK4 complex is attributed to Cyclin D1 regulation. Cyclin D1 is upregulated by transcription factors (TF) including Myc [3, 4] and AP-1 [5]. It is targeted for degradation following phosphorylation on T286 by GSK3β [6] and subjected to GSK3β-independent degradation [7]. CDK2 is present in large excess compared to all other components (Fig 2B–2O), suggesting that Cyclin E is always present in complex with CDK2. Gene expression of Cyclin E is induced by the transcription factor (TF) Myc [8] and E2F-1 [9]. Negative regulation includes basal degradation as well as degradation induced by GSK3β [10]. Phosphorylation of CDK2 at T160 occurs in the nucleus [11]. Enzymatically active CDK2 requires phosphorylation at T160 and the release of p21 [12].

3.2 p21

p21 expression can be induced by the transcription factor (TF) p53 [13] as well as free E2F-1 [14]. We assume that E2F-1 loading on the p21 promoter requires cooperative p53 binding. Degradation of p21 is regulated by two mechanisms: (i) After phosphorylation by GSK3β and ERK2 free p21 is exported to the cytoplasm and degraded [15, 16]. (ii) p21 is phosphorylated and subsequently targeted for degradation by either active CDK2 [17] or ERK [15]. Cyclin D1, CDK2 and CDK4 lack a nuclear localization sequence (NLS) [18] and require p21, which contains a NLS, for nuclear import in primary rat hepatocytes [19]. This process can be inhibited by phosphorylation of p21 by Akt [20].

3.3 Rb

During G1, Rb is bound to E2F-1, which prevents E2F-1 from being activated and inducing transcription of S phase genes [21]. We consider both basal and induced synthesis of Rb [22] by free E2F. Rb is a stable protein with a half-life of at least 10 hours [23] and E2F-1 bound Rb is assumed to be even more stable due to "cooperative stability" [24, 25]. Active CDK2 and CDK4 inhibit Rb by phosphorylation of multiple sites leading to the release of E2F-1 and the induction of S phase genes [26]. Based on the observations that a combination of CDK4/6 and CDK2 kinase activities is required for full phosphorylation of Rb [27] and the inability of Cyclin E:CDK2 to phosphorylate Rb in the absence of prior phosphorylation by Cyclin D1:CDK4 complexes [28], we focus on sequential phosphorylation of Rb on S788 and S800/S804 (corresponding to S795 and S807/S811 for human Rb) known to be specific for CDK4 [29] and CDK2 [30], respectively. In our model Rb phosphorylation by both CDKs is described by Hill kinetics with a fixed Hill coefficient of 3 to emphasize that several sites are phosphorylated. The first phosphorylation of Rb by active CDK4 at S788 allows partial transcriptional activation of E2F-1 still bound to Rb. Partially active E2F-1 and free E2F-1 induce the expression of selected genes, including the Cyclin E gene [9, 31]. The second phosphorylation step of Rb at S800/S804 leads to dissociation of the complex, releasing fully active E2F-1. Rb dephosphorylation occurs sequentially [32]; Cyclin D1:CDK4 sites are assumed to be dephosphorylated first, followed by Cyclin E:CDK2 sites. Since it was reported that the dephosphorylation of Rb can be inhibited by active CDK2 [33, 34], we included this positive feedback loop into our model.

3.4 E2F-1 and DNA synthesis

Free E2F-1 induces its own synthesis [35]. Furthermore, the transcription factor (TF) Myc leads to E2F-1 induction [25, 35]. We distinguish the degradation of free E2F-1 from bound E2F-1 as it was shown that bound E2F-1 is more stable [36, 37]. Free E2F-1 indirectly enhances the degradation of CDK-bound p21 [38–41]. Free E2F-1 upregulates the expression of genes contributing to the formation of prereplication complexes and active Cyclin E:pCDK2 T160 stimulates the formation of such complexes [42]. After a delay, this leads to G1/S transition and synthesis of DNA. G1/S transition regulation in unstimulated and HGF-stimulated primary mouse hepatocytes.

4 Description of the model's ODEs

4.1 Definition of the rules used to generate the ODEs

Nº	BioNetGen syntax	Reaction rate	Notes
**Synthesis of cyclin D1**			
1	@cyto:I() → @cyto:C4D1(b)	ks_c4 · tf	Cyclin D1 expression is induced by Myc [3, 4], AP-1 [5] but also other IEGs like CREB [43] or ETS1/2 [44]. The term ks_c4 embeds also its repression by JunB [5].
**Degradation of cyclin D1**			
2	@nuc:C4D1(b) → @nuc:Trash()	kdeg_c4 + kdeg_c4gsk3b · gsk3b	Cyclin D1 is degraded in the cytoplasm by FBX4 after nuclear export following phosphorylation by GSK3β [6]. This process is only feasible on nuclear C4D1 if no inhibitor is bound since they prevent CRM1 binding and export [45]. However, it can be applied to all C4D1 in the cytoplasm since they do not need to be exported. Furthermore, Alt *et al.* [45] showed that phosphorylation of cyclin D is maintained in cyclin D:CDK4:p21 complexes so we assume that this process is independent of CKI binding. We also include a basal degradation term.
3	@nuc:C4D1(b!+) → @nuc:Trash()	kdeg_c4 DeleteMolecules	
4	@cyto:C4D1() → @cyto:Trash()	kdeg_c4 + kdeg_c4gsk3b · gsk3b DeleteMolecules	
**Synthesis of cyclin E**			
5	@cyto:I() → @cyto:C2E(b,T160∼U)	ks_c2myc·tf + ks_c2e2f·(actfreeE2F + actboundE2F)	Cyclin E expression is induced by Myc [8] and E2F (free or bound to phosphorylated Rb after release of HDAC [9]).
**Degradation of cyclin E**			
6	C2E() → Trash()	kdeg_c2 +kdeg_c2gsk3b · gsk3b DeleteMolecules	The constitutive turnover of monomeric cyclin E by Cullin 3 [46] can not be explicitly included since we do not differentiate free cyclin E from CDK2-bound cyclin E. But it is partly included in the parameter kdeg_c2, which describes a basal degradation for all cyclin E. kdeg_c2gsk3b describes a degradation process occurring after phosphorylation on T380 by GSK3β and ubiquitination by dimeric Fbw7 [10].
7	C2E(b,T160∼P) → Trash()	kdeg_c2c2gsk3b · gsk3b +kdeg_c2	Cyclin E is degraded after double phosphorylation followed by ubiquitination by monomeric Fbw7. Phosphorylation occurs on T380 GSK3β and S384 by CDK2 [10, 47]. Since CDK2 kinase activity is required, only free C2E complexes are affected. kdeg_c2c2gsk3b describes cyclin E degradation after double phosphorylation by GSK3β and C2E followed by ubiquitination by monomeric Fbw7. kdeg_c2 includes a double phosphorylation by C2E alone followed by ubiquitination by monomeric Fbw7 as well as a single phosphorylation by C2E followed by ubiquitination by dimeric Fbw7.
**CDK2 kinase activation**			
8	@nuc:C2E(b,T160∼U) ↔ @nuc:C2E(b,T160∼P)	kp_c2cak, kdp_c2cak	CDK2 kinase is activated by phosphorylation on T160 in the nucleus by CAK in its free form [48]. Indeed, p27 binding blocks access to ATP on the CDK2 active site but its phosphorylation on Tyrosine induces its release [49, 50]. We suppose this is also the case after p21 binding.

Continued on next page

Nº	BioNetGen syntax	Reaction rate	Notes
**Synthesis of p21**			
9	@cyto:I() → @cyto:p21(b)	(ks_p21e2f · actfreeE2F +ks_p21p53) · tfp21	p21 expression is induced by p53 [19] and E2F transcription factors [51]. We assume that E2F requires a cofactor (here p53) like for its auto-transcription.
**Binding of p21 to C4D1/C2E**			
10	C4D1(b) + p21(b) ↔ C4D1(b!1).p21(b!1)	kb_p21c4, kd_p21c4	p21 can bind either C2E or C4D1 [52] (possibly in both cytoplasm and nucleus).
11	C2E(b) + p21(b) ↔ C2E(b!1).p21(b!1)	kb_p21c2, kd_p21c2	
**Import of p21 and with them of C4D1/C2E by piggybacking**			
12	@cyto:p21() → @nuc:p21()	$\text{kimport}/V_{ratio}$ MoveConnected	p21 nuclear import and the import of its associated cyclin:CDK complex was confirmed with C2E and C4D1 in primary hepatocytes [19, 53]. Nuclear import of p21 is inhibited by phosphorylation on T145 by Akt [20, 54]. The flag "MoveConnected" is actually not used since all components of a molecule are moved when using the notation @cyto:xx. It would be needed if we used xx@cyto. But we still leave it for clarity.
12b	@cyto:p21() → @cyto:Trash()	$\text{kimport} \cdot (1-1/V_{ratio})$	Those terms were added to adjust the reaction rate for cytoplasmic p21 as it should depend on the volume ratio. Furthermore no "DeleteMolecules" is needed as the whole complex should be concerned.
**Degradation of p21**			
13	@nuc:p21(b!+) → @nuc:Trash()	(kdeg_p21erkskp2 · erk +kdeg_p21c2skp2 · actC2E +kdeg_p21skp2) · actfreeE2F DeleteMolecules	p21 is phosphorylated by CDK2 and ERK2 [15] on S130 and finally degraded after ubiquitination by Skp2 (here approximated by E2F as it is directly expressed by free E2F). We assume it must be bound to a CDK to be presented to the proteasome [17]. However, although phosphorylation stimulates p21 ubiquitination, it is not absolutely required [17]. In Hela cells, phosphorylation by ERK2 seems to induce nuclear export of p21 before degradation [15]. However, we omit this possibility as Skp2 is only nuclear. Also, since cyclin:CDK complexes bind preferably the cy1 domain of p21 and are then inhibited and since only cyclin E:CDK2 bound to cy2 are active, we assume that only CDK-bound p21 are phosphorylated by active cyclin E:CDK2 complexes [55].
14	p21(b) → Trash()	kdeg_p21erk · erk +kdeg_p21gsk3b · gsk3b	GSK3β and ERK2 were shown to induce p21 degradation after phosphorylation on T57 [56]. But since this site is situated in its cdk-binding region, we suppose it affects only free p21.
**Synthesis of Rb**			
15	@nuc:I() → @nuc:rb(b,S788~U,S800~U)	ks_rb +ks_rbe2f·actfreeE2F	Basal expression and E2F dependent synthesis of Rb [22].
**Degradation of Rb**			
16	@nuc:rb(b) → @nuc:Trash()	kdeg_rbfree	Bound Rb is very stable [25] and we suppose that its half-life decreases when it is released like in the case of E2F.
17	@nuc:rb(b!+) → @nuc:Trash()	kdeg_rbbound DeleteMolecules	
18	@nuc:rb() → @nuc:Trash()	kdeg_rbp21·actfreep21 DeleteMolecules	p21 mediates retinoblastoma protein degradation [57].

Continued on next page

Nº	BioNetGen syntax	Reaction rate	Notes
**Synthesis of E2F**			
19	@nuc:I() → @nuc:e2f(b)	(ks_e2fmyc +ks_e2fe2f · actfreeE2F) · tf	E2F induces its own transcription but Myc was shown to be essential for the activation of the E2F genes. It binds the E2F gene promoter to allow loading of E2F1 [35]. Myc is also supposed to be able to stimulate E2F transcription on its own as performed in [25].
**Degradation of E2F**			
20	@nuc:e2f(b) → @nuc:Trash()	kdeg_e2ffree	We distinguish free E2F from bound E2F as it was shown that bound E2F is more stable [36].
21	@nuc:e2f(b!+) → @nuc:Trash()	kdeg_e2fbound DeleteMolecules	
**Binding of E2F and Rb**			
22	@nuc:rb(b,S788∼U,S800∼U) + @nuc:e2f(b) ↔ @nuc:rb(b!1,S788∼U,S800∼U).e2f(b!1)	kb_rbe2f, kd_rbe2f	Rb binds E2F only under its un- or hypophosphorylated form. Hyperphosphorylated Rb (double-phosphorylated in our case) does not bind E2F [58].
23	@nuc:rb(b,S788∼P,S800∼U) + @nuc:e2f(b) ↔ @nuc:rb(b!1,S788∼P,S800∼U).e2f(b!1)	kb_rbpe2f, kd_rbpe2f	
**Phosphorylation of Rb and release of E2F**			
24	@nuc:rb(b,S788∼U,S800∼U) → @nuc:rb(b,S788∼P,S800∼U)	kcatp_rbc4 · actC4D1p21 · $\text{actfreeRb}^{\text{nrb}-1}$ $/(\text{Km\_prb}^{\text{nrb}}$ $+\text{actfreeRb}^{\text{nrb}})$	We suppose a sequential phosphorylation of Rb and although it contains many CDK phosphorylation sites [59], we focus only on 2 sites known to be specific to either CDK4 or CDK2. This is motivated by Hatakeyama *et al.* [27] who demonstrated that a combination of CDK4/6 and CDK2 kinase activities are necessary for full phosphorylation of Rb. This was partially confirmed by Connell-Crowley *et al.* [29] who showed that phosphorylation by CDK4 is not sufficient to inactivate Rb although it might be required for initiating its inactivation. They further showed that phosphorylation by CDK2 alone or CDK4 alone has little effect on E2F inhibition by Rb and that both Cyclin D:CDK4 and cyclin E:CDK2 complexes can prevent E2F association. The first site is thus phosphorylated by active C4D1 (free C4D1 is not supposed to be active as the cyclin D:CDK4 complex is unstable and needs p21 for assembly [60]. So free C4D1 might then represent mostly free cyclin D1). p21 does not reduce CDK4 activity [61].
25	@nuc:rb(b!1,S788∼U,S800∼U).e2f(b!1) → @nuc:rb(b!1,S788∼P,S800∼U).e2f(b!1)	kcatp_rbc4 · actC4D1p21 · $\text{actboundRb}^{\text{nrb}-1}$ $/(\text{Km\_prb}^{\text{nrb}}$ $+\text{actboundRb}^{\text{nrb}})$	
26	@nuc:rb(b,S788∼P,S800∼U) → @nuc:rb(b,S788∼P,S800∼P)	kcatp_rbc2 · actC2E · $\text{actfreeRbphospho}^{\text{nrb}-1}$ $/(\text{Km\_prb}^{\text{nrb}}$ $+\text{actfreeRbphospho}^{\text{nrb}}$	We assume here that phosphorylation on the second site is only possible after prior phosphorylation on the first site by CDK4 as performed in [62]. We also summarized E2F release and Rb double phosphorylation into one reaction.
27	@nuc:rb(b!1,S788∼P,S800∼U).e2f(b!1) → @nuc:rb(b,S788∼P,S800∼P) + @nuc:e2f(b)	kcatp_rbc2 · actC2E · $\text{actboundRbphospho}^{\text{nrb}-1}$ $/(\text{Km\_prb}^{\text{nrb}}$ $+\text{actboundRbphospho}^{\text{nrb}}$	

Continued on next page

Nº	BioNetGen syntax	Reaction rate	Notes
**Rb dephosphorylation**			
28	@nuc:rb(b,S788~P,S800~P) → @nuc:rb(b,S788~P,S800~U)	kcatdp_rbc2 · actC2E · act-freeRb2phospho^{nrb-1} /(Km_dprb^{nrb} +act-freeRb2phospho^{nrb} · 1 /(1+kinh_pp1 · actC2E)	Rb is dephosphorylated by the PP1 phosphatase in a sequential fashion [63]. PP1 is however phosphorylated on T320 by active CDK2, which inhibits its phosphatase activity [34].
29	@nuc:rb(b,S788~P,S800~U) → @nuc:rb(b,S788~U,S800~U)	kcatdp_rbc4 · actC2E · actfreeRbphos-pho^{nrb-1} /(Km_dprb^{nrb} +act-freeRbphospho^{nrb} · 1 /(1+kinh_pp1 · actC2E)	
30	@nuc:rb(b!1,S788~P,S800~U).e2f(b!1) → @nuc:rb(b!1,S788~U,S800~U).e2f(b!1)	kcatdp_rbc4 · actC2E · actboundRbphos-pho^{nrb-1} /(Km_dprb^{nrb} +act-boundRbphospho^{nrb} · 1 /(1+kinh_pp1 · actC2E)	
**DNA synthesis**			
31	@nuc:dnapre() → @nuc:dnapre1()	k_dna·actC2E·actfreeE2F	Free E2F upregulates not only the expression of G1 phase genes but also of genes contributing to the formation of prereplication complexes such as cdc6, MDM2-7 or NPAT. Cyclin E:CDK2 complexes stimulate the formation of such complexes as well as transcription of histone encoding genes by phosphorylating NPAT and MCM2-7 [42].
31b	@nuc:dnapre1() → @nuc:dnapre2()<br>@nuc:dnapre2() → @nuc:dnapre3()<br>@nuc:dnapre3() → @nuc:dnapre4()<br>@nuc:dnapre4() → @nuc:dnapre5()<br>@nuc:dnapre5() → @nuc:Trash()	k_delay<br>k_delay<br>k_delay<br>k_delay<br>k_delay	This chain simulates a delay between the activation of S phase proteins and the actual DNA synthesis [64].

Table S3. Documentation of the rules describing the dynamical model. Input variables describing HGF stimulated signalling pathways are described in purple. Compartments are represented in blue, abbreviations as listed in Table S4 in green. Volume parameters are depicted in orange. Species I() and Trash() are set to be constant (no ODE is generated) and equal to 1 and 0 respectively, in both nucleus and cytoplasm.

4.2 Species definitions used in the reaction rates

Abbreviation	Species
actC4D1p21	@nuc:C4D1(b!1).p21(b!1)
actC2E	@nuc:C2E(b,T160~P)
actfreeE2F	@nuc:e2f(b)
actboundE2F	@nuc:rb(b!1,S788~P,S800~U).e2f(b!1)
actfreep21	@nuc:p21(b,T145~U)
actfreeRbphospho	@nuc:rb(b,S788~P,S800~U)
actfreeRb2phospho	@nuc:rb(b,S788~P,S800~P)
actboundRbphospho	@nuc:rb(b!1,S788~P,S800~U).e2f(b!1)
actfreeRb	@nuc:rb(b,S788~U,S800~U)
actboundRb	@nuc:rb(b!1,S788~U,S800~U).e2f(b!1)

Table S4. Species abbreviations used in the reaction rules.

5 Algebraic equations

5.1 Algebraic equations describing the signal transduction pathways

Parameter	Definition
erk	$= (1 - \mathrm{inherk}) \cdot ((1 - \mathrm{aerk}) \cdot (f(\mathrm{hgf}, \mathrm{perk}, \mathrm{nerk}) + \mathrm{aerk})$
akt	$= (1 - \mathrm{inhakt}) \cdot ((1 - \mathrm{aakt}) \cdot (f(\mathrm{hgf}, \mathrm{pakt}, \mathrm{nakt}) + \mathrm{aakt})$
gsk3b	$= 1 - \mathrm{akt}$
tf	$= (1 - \mathrm{atf}) \cdot (\mathrm{erk} \cdot (1 - \mathrm{gsk3b})) + \mathrm{atf}$
tfp21	$= (1 - \mathrm{inhp53}) \cdot \mathrm{tf}$

where $f(\mathrm{x}, \mathrm{p}, \mathrm{n}) = (\mathrm{p}^{\mathrm{n}} + 1) \cdot \dfrac{\mathrm{x}^{\mathrm{n}}}{\mathrm{x}^{\mathrm{n}} + \mathrm{p}^{\mathrm{n}}}$ [65]

Table S5. Signal transduction network controlled by HGF. These equations describe the level of activity reached for the input variables erk, akt, gsk3b, tf and tfp21 at steady state for a given HGF concentration. HGF, inherk, inhakt and inhp53 are inputs that are set according to the experiment (inherk, inhakt and inhp53 are set to 1 in case of treatment with the MEK inhibitor U0126, the Akt Inhibitor VIII and the p53 inhibitor Pifithrin, respectively).

5.2 Definitions of the model's observables

Measured concentration	Equation
cyclin E:CDK2	scale_TotcycECDK2·(V_{nuc}·@nuc:C2E() + V_{cyto}·@cyto:C2E()) / (V_{nuc}+V_{cyto})
pCDK2 T160	scale_TotCDK2T160·(V_{nuc}·@nuc:C2E(T160~P) + V_{cyto}·@cyto:C2E(T160~P)) / (V_{nuc}+V_{cyto})
cyclin D:CDK4	scale_TotcycDCDK4·(V_{nuc}·@nuc:C4D1(b!+) + V_{cyto}·@cyto:C4D1(b!+)) / (V_{nuc}+V_{cyto})
Total p21	scale_Totp21·(V_{nuc}·@nuc:p21() + V_{cyto}·@cyto:p21()) / (V_{nuc}+V_{cyto})
CDK2:p21	scale_Totp21CDK2·(V_{nuc}·@nuc:p21(b!1).C2E(b!1) + V_{cyto}·@cyto:p21(b!1).C2E(b!1)) / (V_{nuc}+V_{cyto})
Total E2F-1	(scale_TotE2F+scale_TotRb)·V_{nuc}·@nuc:e2f() / (V_{nuc}+V_{cyto})
Total Rb	scale_TotRb·V_{nuc}·@nuc:rb() / (V_{nuc}+V_{cyto})
pRb S788	scale_PhosRbS788·V_{nuc}·@nuc:rb(S788~P) / (V_{nuc}+V_{cyto})
pRb S800/S804	scale_PhosRbS800·V_{nuc}·@nuc:rb(S800~P) / (V_{nuc}+V_{cyto})
DNA content	2 - (@nuc:dnapre() + @nuc:dnapre1() + @nuc:dnapre2() + @nuc:dnapre3() + @nuc:dnapre4() + @nuc:dnapre5())

Table S6. List of observables. Those equations have to be expanded to all species corresponding to the associated patterns.

6 Parameter descriptions and start values before fitting

Parameter	Description	Value	Fixed	References
V_{nuc}	Nuclear volume	0.25	*	
V_{cyto}	Cytoplasmic volume	12.67	*	
V_{ratio}	Ratio nuclear/cytoplasmic volumes	V_{nuc}/V_{cyto}	*	
perk	EC50 of ERK	0.1		
nerk	Hill coefficient for ERK activation	4		
pakt	EC50 of Akt	0.0348	*	
nakt	Hill coefficient for Akt activation	1.0957	*	
aerk	Basal ERK activity	0.16	*	
aakt	Basal Akt activity	0.53	*	
atf	Basal TF activity	0.2		
ks_c4	Synthesis rate of C4D1	100		
kdeg_c4	Basal degradation rate of C4D1	1.5		Cyclin D1 has a half-life of 3.5h if T286 is mutated [60]
kdeg_c4gsk3b	Degradation rate of C4D1 after phosphorylation by GSK3β	1.5		
ks_c2myc	Synthesis rate of C2E by Myc	100		
ks_c2e2f	Synthesis rate of C2E by E2F	100		
kdeg_c2c2gsk3b	Degradation rate of C2E after phosphorylation by C2E and GSK3β	0.2		
kdeg_c2gsk3b	Degradation rate of C2E after phosphorylation by GSK3β	0.3		half-life of cyclin E = 30 minutes [25] (ln(2)/0.5-ln(2)/2)
kdeg_c2	Basal degradation rate of C2E	0.3466		half-life of 2h if T380 is mutated [66]
kp_c2cak	Phosphorylation rate of C2E by CAK	kdp_c2cak+kc2cak		
kdp_c2cak	Phosphorylation rate of C2E by CAK	0.1		
kc2cak		10		
ks_p21p53	Synthesis rate of p21 by p53	100		
ks_p21e2f	Synthesis rate of p21 by E2F	100		
kdeg_p21erkskp2	Degradation rate of p21 after phosphorylation by ERK and ubiquitination by Skp2	0.1		
kdeg_p21c2skp2	Degradation rate of p21 after phosphorylation by C2E and ubiquitination by Skp2	1		
kdeg_p21skp2	Degradation rate of p21 after ubiquitination by Skp2 (without phosphorylation)	0.1		

Continued on next page

Parameter	Description	Value	Fixed	References
kdeg_p21gsk3b	Degradation rate of p21 after phosphorylation on T57 by GSK3β	1		
kdeg_p21erk	Degradation rate of p21 after phosphorylation on T57 by ERK	1		
kd_p21c4	Dissociation rate of p21:C4D1 complexes	Kd_p21c4$\cdot$kb_p21c4		$3600 \cdot 1.89 \cdot 10^{-4} \cdot h^{-1}$. Value from [50] for p27:C4D1.
kb_p21c4	Binding rate of p21:C4D1 complexes	4.6728		$12.98 \cdot 10^{5} \cdot 3600 \cdot 10^{-9} nM^{-1} \cdot h^{-1}$. Value from [50] for p27:C4D1
Kd_p21c4	Dissociation constant of p21:C4D1 complexes	0.1456		0.6804/4.6728
kd_p21c2	Dissociation rate of p21:C2E complexes	Kd_p21c2$\cdot$kb_p21c2		
kb_p21c2	Binding rate of p21:C2E complexes	4.6728		
Kd_p21c2	Dissociation constant of p21:C2E complexes	0.1456		
kinh_p21akt	Inhibition of p21 import after Akt phosphorylation	10		
kimport	Import rate of p21	ki/(1+kinh_p21akt*akt)		
ki		1		
ks_e2fe2f	Autotranscription of E2F-1	100		
ks_e2fmyc	Synthesis rate of E2F by Myc	10		
kdeg_e2fbound	Degradation rate of bound E2F	0.0693		[36]
kdeg_e2ffree	Degradation rate of free E2F	kdeg_e2fbound+kdege2fplus		Half-life of 2 hours [36]
kdege2fplus		0.2773		
ks_rb	Basal synthesis rate of Rb	10		
ks_rbe2f	Synthesis rate of Rb by E2F	100		
kdeg_rbp21	Degradation rate of Rb induced by p21	0.05		
kdeg_rbbound	Degradation rate of bound Rb	0.0693		Half-life of 12 hours [25]
kdeg_rbfree	Degradation rate of free Rb	kdeg_rbbound+kdegrbplus		Half-life of 6 hours [25]
kdegrbplus		0.2773		
kb_rbe2f	Binding rate of Rb:E2F complexes	10		
kd_rbe2f	Dissociation rate of Rb:E2F complexes	kb_rbe2f$\cdot$Kd_rb_e2f		
Kd_rb_e2f	Dissociation constant of Rb:E2F complexes	110		$0.11 \cdot 10^{-6}$M, [32]
kb_rbpe2f	Binding rate of phosphorylated Rb:E2F complexes	10		
kd_rbpe2f	Dissociation rate of phosphorylated Rb:E2F complexes	kb_rbpe2f$\cdot$Kd_rbp_e2f		
Kd_rbp_e2f	Dissociation constant of phosphorylated Rb:E2F complexes	370		$0.37 \cdot 10^{-6}$M, [32]

Continued on next page

Parameter	Description	Value	Fixed	References
kcatprbc4	Phosphorylation rate of Rb by C4D1	100		
kcatp_rbc4	Phosphorylation rate of Rb by C4D1	kcatprbc4·(1-inhc4d1)		
kcatp_rbc2	Phosphorylation rate of Rb by C2E	100		
kcatdp_rbc4	Dephosphorylation rate of Rb after phosphorylation by C4D1	100		
kcatdp_rbc2	Dephosphorylation rate of Rb after phosphorylation by C2E	100		
kinh_pp1	Inhibition of PP1 activity after phosphoryaltion by C2E	10		
Km_dprb	Michaelis constant for Rb dephosphorylation	10		[25]
Km_prb	Michaelis constant for Rb phosphorylation	920		[25]
nrb	Hill coefficient for Rb (de)phosphorylation	3	*	
k_dna	Induction rate of DNA synthesis	1		
k_delay	Parameter for inclusion of a delay before actual DNA synthesis	1		

Table S7. List of parameters and their default start values before parameter fitting. Default units: concentration in nM, time in hours, volume in pl. A $*$ in the column "Fixed" denotes a parameter that was fixed during the fitting process.

7 Determination of scaling factors and initial values

Quantitative data is available for all total concentrations at one give time point (t=56h). From this information, the corresponding scaling factors and initial values can be extracted. Since total concentrations are defined in a volume corresponding to the whole cell, scaling factors must be computed correspondingly.

symbol	description	unit
V_{comp}	volume of a compartment comp, where *comp* can either be *cell*, *nuc* or *cyto* corresponding to the cellular, nuclear or cytoplasmic compartment.	pL
N_A	Avogadro constant (we use the value $6.022 \times 10^{23} mol^{-1}$)	molecules×mol^{-1}
n_{56}	average number of molecules in a cell at t=56 h	molecules
$c_{x,comp}$	concentration at t=x h within a compartment with a volume V_{comp}.	nmol×L^{-1} (nM)
y_x	experimental data at t=x h	a.u.
s	scaling factor	nM^{-1}

Table S8. Parameters to define scaling factors and initial values.

7.1 Cytoplasmic and nuclear volumes of primary mouse hepatocytes

Primary mouse hepatocytes were isolated and seeded at a density of 2×10^5 cells per well in collagen I coated Labtek chambers (Nalgene Nunc). Cells were stained for 30 min with 1 $\mu g/ml$ Vybrant Dil (Invitrogen) and 1:5000 Hoechst 33342 (Sigma-Alldrich) at 37°C in cultivation medium. After three washing steps in cultivation medium cells were analyzed with a Leica SP2 confocal microscope (63 × /1.4 oil objective) using an opened pinhole (2.6 Airy units). Cells were excited with 345 nm (Hoechst DNA stain) and 549 nm (Vybrant Dil) and z-stacks were recorded. Images were merged with ImageJ and adjustments of brightness and contrast were performed using Photoshop CS2. For volume determination recorded z-stacks were deconvoluted by ImageJ. Cell boundaries were marked by hand for all stacks and volumes were estimated by voxel count.

7.2 Scaling factors

First, the total concentrations are determined at t=56h:

$$c_{56,cell} = \frac{n_{56}}{N_A \cdot V_{cell} \cdot 10^{-21}} \quad (1)$$

The associated scaling factor can then be computed using:

$$s = \frac{y_{56}}{c_{56,cell}} \quad (2)$$

Using those equations, we obtain the scaling factors listed in Table S9.

protein	n_{tot}	y_{tot}	s	y_0	$c_{0,cell}$
E2F1	452	1.6699	**28.7418**	0.0334	0.0012
Rb	49697	1.6637	**0.2605**	0.1293	0.4964
p21	99346	2.2066	**0.1728**	0.0208	0.1204
C4D1[1]	27575	2.0026	**0.5651**	–	0
C2E[1]	52764	1.2808	**0.1889**	0.0995	0.5267

Table S9. Computed scaling factors (s) and initial values for total concentrations ($c_{0,cell}$).

[1]For the cyclin:CDK complexes, the number of molecules of both Cyclin and CDK were measured separately and the lower was chosen to compute the scaling factors.

7.3 Initial values

To compute initial values, we consider each species in their respective compartment (either nucleus or cytoplasm) so that the corresponding value can be computed as follows:

$$c_{0,comp} = \frac{y_0 \cdot V_{cell}}{s \cdot V_{comp}} \tag{3}$$

We first compute $c_{0,cell}$ for all total concentrations to find out which components of a complex are in excess. Table S9 reveals that Rb and p21 is in excess compared to E2F and C2E, respectively.

We suppose that all E2F are bound to unphosphorylated Rb. The remaining Rb are free and unphosphorylated. Furthermore, we assume that all p21 are bound to unphosphorylated C2E in the nucleus and the remaining C2E are unphosphorylated and in the cytoplasm. We obtain the initial values listed in Table S10. All other species are initially set to 0.

**species**	**initial concentration** (in nM)
@nuc::e2f(b!1).rb(S788~U,S800~U,b!1)	0.0601
@nuc::rb(S788~U,S800~U,b)	25.5914
@nuc::C2E(T160~U,b!1).p21(b!1)	6.2223
@cyto::C2E(T160~U,b)	0.4150

Table S10. Initial values for the model species

8 Calibration of the mathematical model

Model calibration was performed by estimation of model parameters to minimize an objective function that corresponds to the distance of model simulation to experimental data, taking the errors of the data into account. The lower the resulting value of the objective function is, the better the model-data compliance is. Because both our mathematical model and our quantitative data are of high complexity, parameter estimation is challenging. To ensure that the best parameter values are found to represent the experimental data, we performed 1000 parameter rounds with random initial parameter guesses. We have previously shown that for reliable parameter estimation, the optimum has to be discovered several times [67]. Even if a parameter set is found that represents a global minimum in parameter space, the problem of non-identifiability may arise [68]. One reason for non-identifiability are parameters that can compensate each other leading to the same (minimal) value of the objective function. A technique to deal with this problem is to base model analysis not only on one set of parameters corresponding to the best parameter estimation run. Instead, model simulations and analyses are performed based on several parameter sets that were obtained in different parameter estimation runs [69]. Thus, we based all our subsequent analyses on ten parameter sets which yielded the lowest values for the objective function and therefore represent the best fits (Figure S7A). Model calibration was performed simultaneously based on both the time-resolved signal intensity of G1/S transition components and the DNA content of primary mouse hepatocytes under various treatments. In Figure 4, the model simulations corresponding to these ten parameter sets are displayed as solid lines. As expected from the similar low objective function values of these parameter estimation runs, the ten individual model trajectories are overlapping with little variation.

Supplementary References

1. Chambers, J. M. and Hastie, T. J. **Linear models**. In *Statistical Models in S*. Wadsworth & Brooks/Cole, 1992.

2. Schilling, M., Maiwald, T., Bohl, S., Kollmann, M., Kreutz, C., Timmer, J., and Klingmüller, U. **Computational processing and error reduction strategies for standardized quantitative data in biological networks**. *FEBS J*, 272(24):6400–6411, December 2005.

3. Pérez-Roger, I., Kim, S.-H., Griffiths, B., Sewing, A., and Land, H. **Cyclins D1 and D2 mediate myc-induced proliferation via sequestration of p27^{Kip1} and p21^{Cip1}**. *EMBO J*, 18(19):5310–5320, October 1999.

4. Bernard, S. and Eilers, M. **Control of Cell Proliferation and Growth by Myc Proteins**. In Kaldis, P., editor, *Cell Cycle Regulation*, volume 42 of *Results and Problems in Cell Differentiation*, pages 329–342. Springer-Verlag, 2006.

5. Bakiri, L., Lallemand, D., Bossy-Wetzel, E., and Yaniv, M. **Cell cycle-dependent variations in c-Jun and JunB phosphorylation: a role in the control of cyclin D1 expression**. *EMBO J*, 19(9):2056–2068, May 2000.

6. Lin, D. I., Barbash, O., Kumar, K. G. S., Weber, J. D., Harper, J. W., Klein-Szanto, A. J. P., Rustgi, A., Fuchs, S. Y., and Diehl, J. A. **Phosphorylation-dependent ubiquitination of cyclin D1 by the SCF(FBX4-alphaB crystallin) complex**. *Mol Cell*, 24(3):355–366, November 2006.

7. Alao, J. P. **The regulation of cyclin D1 degradation: roles in cancer development and the potential for therapeutic invention**. *Mol Cancer*, 6:24, 2007.

8. Pérez-Roger, I., Solomon, D. L., Sewing, A., and Land, H. **Myc activation of cyclin E/Cdk2 kinase involves induction of *cyclin E* gene transcription and inhibition of p27^{Kip1} binding to newly formed complexes**. *Oncogene*, 14(20):2373–2381, May 1997.

9. Möröy, T. and Geisen, C. **Cyclin E**. *The International Journal of Biochemistry & Cell Biology*, 36(8): 1424–1439, August 2004.

10. Welcker, M. and Clurman, B. E. **FBW7 ubiquitin ligase: a tumour suppressor at the crossroads of cell division, growth and differentiation**. *Nat Rev Cancer*, 8(2):83–93, February 2008.

11. Morgan, D. O. **Principles of CDK regulation**. *Nature*, 374(6518):131–4, 1995.

12. Morgan, D. O. **Cyclin-dependent kinases: engines, clocks, and microprocessors**. *Annual review of cell and developmental biology*, 13:261–91, 1997.

13. He, G., Siddik, Z. H., Huang, Z., Wang, R., Koomen, J., Kobayashi, R., Khokhar, A. R., and Kuang, J. **Induction of p21 by p53 following DNA damage inhibits both Cdk4 and Cdk2 activities**. *Oncogene*, 24(18):2929–43, 2005.

14. Gartel, A. L., Goufman, E., Tevosian, S. G., Shih, H., Yee, A. S., and Tyner, A. L. **Activation and repression of p21(WAF1/CIP1) transcription by RB binding proteins**. *Oncogene*, 17(26):3463–9, 1998.

15. Hwang, C. Y., Lee, C., and Kwon, K.-S. **Extracellular signal-regulated kinase 2-dependent phosphorylation induces cytoplasmic localization and degradation of p21^{Cip1}**. *Mol Cell Biol*, 29(12): 3379–3389, June 2009.

16. Rossig, L., Badorff, C., Holzmann, Y., Zeiher, A. M., and Dimmeler, S. **Glycogen synthase kinase-3 couples AKT-dependent signaling to the regulation of p21Cip1 degradation**. *J Biol Chem*, 277(12): 9684–9, 2002.

17. Bornstein, G., Bloom, J., Sitry-Shevah, D., Nakayama, K., Pagano, M., and Hershko, A. **Role of the SCF^{Skp2} Ubiquitin Ligase in the Degradation of p21^{Cip1} in S Phase**. *The Journal of Biological Chemistry*, 278 (28):25752–25757, July 2003.

18. Bockstaele, L., Coulonval, K., Kooken, H., Paternot, S., and Roger, P. P. **Regulation of CDK4**. *Cell Div*, 1:25, 2006.

19. Wierød, L., Rosseland, C. M., Lindeman, B., Oksvold, M. P., Grøsvik, H., Skarpen, E., and Huitfeldt, H. S. **Activation of the p53-p21(Cip1) pathway is required for CDK2 activation and S-phase entry in primary rat hepatocytes**. *Oncogene*, 27(19):2763–2771, April 2008.

20. Zhou, B. P., Liao, Y., Xia, W., Spohn, B., Lee, M. H., and Hung, M. C. **Cytoplasmic localization of p21Cip1/WAF1 by Akt-induced phosphorylation in HER-2/neu-overexpressing cells**. *Nat Cell Biol*, 3(3):245–252, March 2001.

21. Cobrinik, D. **Pocket proteins and cell cycle control**. *Oncogene*, 24(17):2796–809, 2005.

22. Kim, T. A., Ravitz, M. J., and Wenner, C. E. **Transforming growth factor-β regulation of retinoblastoma gene product and E2F transcription factor during cell cycle progression in mouse fibroblasts.** *J Cell Physiol*, 160(1):1–9, July 1994.

23. Mihara, K., Cao, X. R., Yen, A., Chandler, S., Driscoll, B., Murphree, A. L., T'Ang, A., and Fung, Y. K. **Cell cycle-dependent regulation of phosphorylation of the human retinoblastoma gene product**. *Science*, 246(4935):1300–3, 1989.

24. Buchler, N. E., Gerland, U., and Hwa, T. **Nonlinear protein degradation and the function of genetic circuits**. *Proc Natl Acad Sci U S A*, 102(27):9559–64, 2005.

25. Yao, G., Lee, T. J., Mori, S., Nevins, J. R., and You, L. **A bistable Rb-E2F switch underlies the restriction point**. *Nat Cell Biol*, 10(4):476–482, April 2008.

26. Sherr, C. J. **The Pezcoller lecture: cancer cell cycles revisited**. *Cancer Res*, 60(14):3689–95, 2000.

27. Hatakeyama, M., Brill, J. A., Fink, G. R., and Weinberg, R. A. **Collaboration of G_1 cyclins in the functional inactivation of the retinoblastoma protein**. *Genes Dev*, 8(15):1759–1771, August 1994.

28. Lundberg, A. S. and Weinberg, R. A. **Functional inactivation of the retinoblastoma protein requires sequential modification by at least two distinct cyclin-cdk complexes**. *Mol Cell Biol*, 18(2):753–61, 1998.

29. Connell-Crowley, L., Harper, J. W., and Goodrich, D. W. **Cyclin D1/Cdk4 regulates retinoblastoma protein-mediated cell cycle arrest by site-specific phosphorylation**. *Mol Biol Cell*, 8(2):287–301, February 1997.

30. Rickheim, D. G., Nelsen, C. J., Fassett, J. T., Timchenko, N. A., Hansen, L. K., and Albrecht, J. H. **Differential regulation of cyclins D1 and D3 in hepatocyte proliferation**. *Hepatology*, 36(1):30–8, 2002. URL `http://www.ncbi.nlm.nih.gov/pubmed/12085346`.

31. Trimarchi, J. M. and Lees, J. A. **Sibling rivalry in the E2F family**. *Nat Rev Mol Cell Biol*, 3(1):11–20, 2002. URL `http://www.ncbi.nlm.nih.gov/pubmed/11823794`.

32. Rubin, S. M., Gall, A.-L., Zheng, N., and Pavletich, N. P. **Structure of the Rb C-terminal domain bound to E2F1-DP1: a mechanism for phosphorylation-induced E2F release**. *Cell*, 123(6):1093–1106, December 2005.

33. Berndt, N., Dohadwala, M., and Liu, C. W. **Constitutively active protein phosphatase 1alpha causes Rb-dependent G1 arrest in human cancer cells**. *Curr Biol*, 7(6):375–86, 1997.

34. Liu, C. W., Wang, R.-H., Dohadwala, M., Schönthal, A. H., Villa-Moruzzi, E., and Berndt, N. **Inhibitory phosphorylation of PP1α catalytic subunit during the G_1/S transition**. *J Biol Chem*, 274(41): 29470–29475, October 1999.

35. Leung, J. Y., Ehmann, G. L., Giangrande, P. H., and Nevins, J. R. **A role for Myc in facilitating transcription activation by E2F1**. *Oncogene*, 27(30):4172–4179, July 2008.

36. Helin, K. **Regulation of cell proliferation by the E2F transcription factors**. *Current Opinion in Genetics & Development*, 8(1):28–35, February 1998.

37. Hofmann, F., Martelli, F., Livingston, D. M., and Wang, Z. **The retinoblastoma gene product protects E2F-1 from degradation by the ubiquitin-proteasome pathway**. *Genes Dev*, 10(23):2949–59, 1996.

38. Bilodeau, M., Talarmin, H., Ilyin, G., Rescan, C., Glaise, D., Cariou, S., Loyer, P., Guguen-Guillouzo, C., and Baffet, G. **Skp2 induction and phosphorylation is associated with the late G1 phase of proliferating rat hepatocytes**. *FEBS Lett*, 452(3):247–53, 1999.

39. Wei, W., Ayad, N. G., Wan, Y., Zhang, G. J., Kirschner, M. W., and Kaelin, W. G., J. **Degradation of the SCF component Skp2 in cell-cycle phase G1 by the anaphase-promoting complex**. *Nature*, 428 (6979):194–8, 2004.

40. Yung, Y., Walker, J. L., Roberts, J. M., and Assoian, R. K. **A Skp2 autoinduction loop and restriction point control**. *J Cell Biol*, 178(5):741–7, 2007.

41. Zhang, L. and Wang, C. **F-box protein Skp2: a novel transcriptional target of E2F**. *Oncogene*, 25 (18):2615–27, 2006.

42. Woo, R. A. and Poon, R. Y. C. **Cyclin-dependent kinases and S phase control in mammalian cells**. *Cell Cycle*, 2(4):316–324, 2003.

43. Coqueret, O. **Linking cyclins to transcriptional control**. *Gene*, 299(1-2):35–55, October 2002.

44. Albanese, C., Johnson, J., Watanabe, G., Eklund, N., Vu, D., Arnold, A., and Pestell, R. G. **Transforming p21ras mutants and c-Ets-2 activate the cyclin D1 promoter through distinguishable regions**. *J Biol Chem*, 270(40):23589–23597, October 1995.

45. Alt, J. R., Gladden, A. B., and Diehl, J. A. **p21(Cip1) Promotes cyclin D1 nuclear accumulation via direct inhibition of nuclear export**. *J Biol Chem*, 277(10):8517–8523, March 2002.

46. McEvoy, J. D., Kossatz, U., Malek, N., and Singer, J. D. **Constitutive turnover of cyclin E by Cul3 maintains quiescence**. *Mol Cell Biol*, 27(10):3651–3666, May 2007.

47. Siu, K. T., Rosner, M. R., and Minella, A. C. **An integrated view of cyclin E function and regulation**. *Cell Cycle*, 11(1):57–64, January 2012.

48. Ray, A., James, M. K., Larochelle, S., Fisher, R. P., and Blain, S. W. **p27Kip1 inhibits cyclin D-cyclin-dependent kinase 4 by two independent modes**. *Mol Cell Biol*, 29(4):986–999, February 2009.

49. Galea, C. A., Wang, Y., Sivakolundu, S. G., and Kriwacki, R. W. **Regulation of cell division by intrinsically unstructured proteins: intrinsic flexibility, modularity, and signaling conduits**. *Biochemistry*, 47(29):7598–7609, July 2008.

50. Ou, L., Waddell, M. B., and Kriwacki, R. W. **Mechanism of Cell Cycle Entry Mediated by the Intrinsically Disordered Protein p27^{Kip1}**. *ACS Chem Biol*, 7(4):678—-682, February 2012.

51. Huang, H. and Tindall, D. J. **Dynamic FoxO transcription factors**. *J Cell Sci*, 120(Pt 15):2479–2487, August 2007.

52. Sherr, C. J. and Roberts, J. M. **CDK inhibitors: positive and negative regulators of G_1-phase progression**. *Genes Dev*, 13:1501–1512, 1999.

53. Albrecht, J. H., Rieland, B. M., Nelsen, C. J., and Ahonen, C. L. **Regulation of G_1 cyclin-dependent kinases in the liver: role of nuclear localization and p27 sequestration**. *Am J Physiol*, 277(6 Pt 1): G1207–G1216, December 1999.

54. Child, E. S. and Mann, D. J. **The intricacies of p21 phosphorylation: protein/protein interactions, subcellular localization and stability**. *Cell Cycle*, 5(12):1313–1319, June 2006.

55. Zhu, H., Nie, L., and Maki, C. G. **Cdk2-dependent Inhibition of p21 stability via a C-terminal cyclin-binding motif**. *J Biol Chem*, 280(32):29282–29288, August 2005.

56. Rössig, L., Badorff, C., Holzmann, Y., Zeiher, A. M., and Dimmeler, S. **Glycogen synthase kinase-3 couples AKT-dependent signaling to the regulation of p21^{Cip1} degradation**. *J Biol Chem*, 277(12): 9684–9689, March 2002.

57. Broude, E. V., Swift, M. E., Vivo, C., Chang, B.-D., Davis, B. M., Kalurupalle, S., Blagosklonny, M. V., and Roninson, I. B. **p21(Waf1/Cip1/Sdi1) mediates retinoblastoma protein degradation**. *Oncogene*, 26 (48):6954–6958, October 2007.

58. Chellappan, S. P., Hiebert, S., Mudryj, M., Horowitz, J. M., and Nevins, J. R. **The E2F transcription factor is a cellular target for the RB protein**. *Cell*, 65(6):1053–1061, June 1991.

59. Knudsen, E. S. and Wang, J. Y. **Differential regulation of retinoblastoma protein function by specific Cdk phosphorylation sites**. *J Biol Chem*, 271(14):8313–8320, April 1996.

60. Cheng, M., Olivier, P., Diehl, J. A., Fero, M., Roussel, M. F., Roberts, J. M., and Sherr, C. J. **The p21^{Cip1} and p27^{Kip1} CDK 'inhibitors' are essential activators of cyclin D-dependent kinases in murine fibroblasts**. *EMBO J*, 18(6):1571–1583, March 1999.

61. LaBaer, J., Garrett, M. D., Stevenson, L. F., Slingerland, J. M., Sandhu, C., Chou, H. S., Fattaey, A., and Harlow, E. **New functional activities for the p21 family of CDK inhibitors**. *Genes Dev*, 11(7):847–862, April 1997.

62. Tashima, Y., Hamada, H., Okamoto, M., and Hanai, T. **Prediction of key factor controlling G1/S phase in the mammalian cell cycle using system analysis**. *J Biosci Bioeng*, 106(4):368–374, October 2008.

63. Rubin, E., Mittnacht, S., Villa-Moruzzi, E., and Ludlow, J. W. **Site-specific and temporally-regulated retinoblastoma protein dephosphorylation by protein phosphatase type 1**. *Oncogene*, 20(29):3776–3785, June 2001.

64. MacDonald, N. **Time delay in simple chemostat models**. *Biotechnol Bioeng*, 18(6):805–812, June 1976.

65. Mitsos, A., Melas, I. N., Morris, M. K., Saez-Rodriguez, J., Lauffenburger, D. A., and Alexopoulos, L. G. **Non Linear Programming (NLP) formulation for quantitative modeling of protein signal transduction pathways**. *PLoS One*, 7(11):e50085, 2012.

66. Won, K.-A. and Reed, S. I. **Activation of cyclin E/CDK2 is coupled to site-specific autophosphorylation and ubiquitin-dependent degradation of cyclin E**. *EMBO J*, 15(16):4182–4193, August 1996.

67. Raue, A., Schilling, M., Bachmann, J., Matteson, A., Schelke, M., Kaschek, D., Hug, S., Kreutz, C., Harms, B. D., Theis, F. J., Klingmuller, U., and Timmer, J. **Lessons learned from quantitative dynamical modeling in systems biology**. *PLoS One*, 8(9):e74335, 2013.

68. Raue, A., Kreutz, C., Maiwald, T., Bachmann, J., Schilling, M., Klingmuller, U., and Timmer, J. **Structural and practical identifiability analysis of partially observed dynamical models by exploiting the profile likelihood**. *Bioinformatics*, 25(15):1923–9, 2009.

69. Chen, W. W., Schoeberl, B., Jasper, P. J., Niepel, M., Nielsen, U. B., Lauffenburger, D. A., and Sorger, P. K. **Input-output behavior of ErbB signaling pathways as revealed by a mass action model trained against dynamic data**. *Mol Syst Biol*, 5:239, 2009.
