# Supplementary material for: T160-phosphorylated CDK2 defines threshold for HGF-dependent proliferation in primary hepatocytes
Source: Mol Syst Biol. 2015 Mar 14;11(3):0795. doi: 10.15252/msb.20156032 (PMC4380929; doi:10.15252/msb.20156032)
Supplement: Supplementary file 3 [file msb0011-0795-sd3.pdf]

T160-phosphorylated CDK2 defines threshold for HGF-dependent proliferation in primary hepatocytes

Stephanie Mueller, Jérémy Huard, Katharina Waldow, Xiaoyun Huang, Lorenza A. D'Alessandro, Sebastian Bohl, Kathleen Börner, Dirk Grimm, Steffen Klamt, Ursula Klingmüller, Marcel Schilling

Corresponding author: Marcel Schilling, DKFZ

Review timeline:

Submission date:	17 January 2014
Editorial Decision:	26 February 2014
Re-submission:	01 August 2014
Editorial Decision:	11 September 2014
Re-submission:	13 January 2015
Editorial Decision:	03 February 2015
Revision received:	12 February 2015
Accepted:	18 February 2015

Editor: Maria Polychronidou

Transaction Report:

(Note: With the exception of the correction of typographical or spelling errors that could be a source of ambiguity, letters and reports are not edited. The original formatting of letters and referee reports may not be reflected in this compilation.)

1st Editorial Decision 26 February 2014

Thank you again for submitting your work to Molecular Systems Biology. We have now heard back from the three referees whom we asked to evaluate your manuscript. As you will see from the reports below, the referees raise substantial concerns on your work, which, I am afraid to say, preclude its publication in Molecular Systems Biology.

Overall, the referees acknowledge that the presented time-resolved quantitative analysis of G1/S transition components in hepatocytes is potentially interesting. However, they raise significant concerns regarding the conclusiveness of the study and point out that as it stands, the main findings are not convincingly supported. Considering the overall substantial points raised, we feel that we have no choice but to return this manuscript with the message that we cannot offer to publish it.

Nevertheless, as the reviewers did have positive words for the goals and the potential relevance of the study, we would like to indicate that we would be willing to reconsider a revised and extended manuscript based on this work. Any new submission would need to include new experimental data demonstrating the proposed central role of CDK2 T160 phosphorylation in controlling hepatocyte proliferation and rigorously addressing the overall concerns raised by the reviewers. We recognize that this would involve substantial further experimentation, but we think that, as pointed out by the referees, such additional analyses are essential for robustly supporting the main conclusions of the study.

A resubmitted work would have a new number and receipt date. If you do decide to follow this

course then it would be helpful to enclose with your re-submission an account of how the work has been altered in response to the points raised in the present review. As you probably understand, we can give no guarantee about the eventual acceptability of a revised version of this work.

I am sorry that the review of your work did not result in a more favorable outcome on this occasion, but I hope that you will not be discouraged from sending your work to Molecular Systems Biology in the future. In any case, thank you for the opportunity to examine this work.

Reviewer #1:

In this study, the authors attempt to quantitatively examine the expression of key cell cycle proteins and during the process of HGF-mediated rat hepatocyte proliferation in culture. One of the interesting features of the study is that they attempt to quantify the abundance of cell cycle proteins and cyclin/cdk/inhibitor complexes at different stages after mitogen stimulation. They then use mathematical models (which are out of my field of expertise) to try to decipher key events. Using these models, they deduce that Thr-160 phosphorylation of cdk2 is "a gate-keeping mechanism contributing to the maintenance of hepatocyte quiescence and the prevention of malignancies." Comments include the following:

1. While the premises are interesting, nothing in this study examines whether cdk2 phosphorylation is related to malignancy, and I wonder why they include this in the abstract.
2. Their data show that phosphorylation of cdk2 at this site correlates with events in the G1/S transition, as have been shown previously in hepatocytes and other systems. However, they have obviously not demonstrated experimentally that this is an important event (although I suspect it is).
3. A number of older studies have indicated that cyclin D1 is the rate limiting step in the progression of hepatocytes through the G1 restriction point and commits the cell to replication. In the current study, the authors suggest that cdk2 phosphorylation is a more important event. It would be useful for the authors to discuss this in light of prior data regarding cyclin D1.
4. The fact that hepatocyte isolation triggers expression of early cell cycle genes has been well documented in the past. The authors should acknowledge this prior body of work.
5. The authors have not shown that "p21 rather than p27 contributed to Cyclin D1:CDK4 complex assembly." Although p21 is binds to the complex, it is not clear from these studies that p21 plays a role in assembly of D1/K4 complexes. In fact, after PH in p21-/- mice, cyclin D1:CDK4 complex activation occurs earlier than in wild-type mice, so p21 is clearly not necessary for this.
6. Obviously, it is critical that the authors have high-quality antibodies and western blots in order to do their quantitative analysis. It would be helpful for them to demonstrate the quality of their antibodies for key proteins (e.g. cyclin E1) by doing controls with blocking peptides or knockdown.
7. The assumptions used in their models may or may not be valid in hepatocytes.

Minor issues:
1. First line of abstract: Many factors contribute to liver regeneration, and I'm not sure that "Liver regeneration is ... primarily achieved by hepatocyte growth factor (HGF)-induced proliferation."
2. The authors should note that older studies have studies the mitogen restriction point in rat hepatocytes (1, 2), and was determined to be at similar time points after plating the cells.

Reviewer #2:

Summary:
This study aims to identify molecular components that regulate DNA replication during the cell cycle in hepatocytes. An understanding of how the cell cycle is controlled could help reveal why these cells divide in response to specific cues, and how uncontrolled division is avoided. To address these questions, the authors performed quantitative and semi-quantitative experimental measurements of cell cycle readouts in primary mouse hepatocytes. These measurements were used to parameterize a computational model that consisted of two parts: a logical model describing inputs, and a chemical kinetic model describing dynamics of cell cycle components. A key conclusion extracted from the model is that phosphorylation of T160 in CDK2 must cross a threshold level for DNA replication to be induced.

General remarks:
The study could be of interest to those involved in quantitative investigation of the cell cycle. The

modeling aspect improves on a previous study of the system that relied on logical modeling only. The experimental measurements seem valuable, covering a fairly large number of readouts at multiple timepoints in the presence and absence of HGF.

However, the primary use of experimental data seems to have been in estimating parameters in the model. Model predictions arising from the proposed mechanisms were not tested much, if at all. As a result, we are not fully convinced of the major conclusion, i.e. the role of CDK2 T160 as a gatekeeper. Can the authors perform tests of model predictions to strengthen their conclusions?

Major points:

1) In some cases, differences between experimental readouts seem to be overstated. For example, it is said that expression of CDK4 remained constant over time while CDK2 increased slightly. From looking at the plots in Fig 2A, it seems that both proteins increased slightly in the HGF-stimulated cells, following an initial drop.

2) Similarly, it is said that CDK2-bound p27 remained constant. The figure indicates that CDK2-bound p27 increases over time, with the stimulated and unstimulated cells looking much the same.

3) As a result of these ambiguities, the meaning of the following sentence is not evident: "The results allowed us to define the following regulated G1/S transition components in primary mouse hepatocytes..." It is unclear how this conclusion was reached. What is the feature shared by all of the components listed? Is this feature *not* shared by the components omitted? For example, why is p21:CDK4 not included?

4) The model diagram in Fig 3B is not easily interpreted (what do the dots on lines mean?), and is not connected to the underlying model in a clear way. Since this diagram represents a rule-based model, the authors may consider taking advantage of published, standardized guidelines for visualizing such models. Arrows in the diagram could be cross-referenced with numbered rules, which seem to already be part of the supplement. These changes would go a long way towards communicating the model's content to a reader.

5) The authors state that they measured basal "activity" of Akt and ERK. What they seem to have actually measured is phosphorylation level of these two proteins. How was phosphorylation level translated into enzymatic activity (if that is what was done) and used in the model?

6) A number of observables are selected without much explanation, and are plotted in Fig S6. In the discussion of this figure in the text, it is stated that "we indeed observed their temporal behavior in the absence and presence of HGF, validating the predictive power of our model". We do not see how one draws conclusions about predictive power (the ability to make testable predictions) based on the fact that a model can simply generate timecourses. Are the molecules represented here particularly amenable to experimental measurement? We would expect not, since many of them refer to specific phosphoforms and bound species.

7) Although the proposed importance of CDK2 T160 is made clear, the underlying mechanisms that bring about this role are not. Why does it rely so much more strongly on HGF than the other readouts? Are there conditions under which it would lose its strong dependence on HGF? Considering these types of questions could help develop the model's actual predictive power.

8) Finally, why was it necessary to use logical modeling? Is it not possible to represent HGF's impact in terms of chemical kinetics?

© European Molecular Biology Organization

Minor points:

1) For a broad audience, it would be helpful if the more specialized terms (e.g., resection, T loop) were defined.

2) In Fig 2 and other similar figures that contain many plots, it would be helpful if the plots were labeled individually (A, B, C, etc.) and referenced in the text. That would make it easier for a reader to quickly go between the data and the discussion.
3) A typo on page 12, 2nd paragraph: "while the G1/S transition components where low" should be "were".

4) In the caption of Fig 2A, the meaning of the vertical dashed lines should be stated. I assume it corresponds to the estimated restriction point.

5) Although the model is described in the main text, it reads more like a review and does not say much about how the information was translated into a model, what assumptions were used, etc. A reader may refer to details in the supplement, but a more concrete description in the text itself would be useful.

6) Table S5, the logical network, is in need of some explanation of what the variables are meant to represent.

Reviewer #3:

Review of the manuscript 'T160-phosphorylated CDK2 defines the threshold for HGF-dependent proliferation in primary hepatocytes' by Mueller et al for MSB. The authors perform a nice time-course experiment of primary hepatocytes exposed to HGF, which induces them to enter the cell cycle. The authors track over 10 distinct protein species quantitatively with dense temporal sampling in both the experiment (HGF induced) and the control (fig. 2). This is a nice experiment since the hepatocytes are relatively well-synchronized and such comprehensive time courses are generally lacking for primary cells. The authors also show that Cdk4, Akt, Mek and p53 inhibitors are required for S-phase in their system (fig. 4b). Also, the authors perform a nice experiment to determine where in this time course the majority of cells are committed to division and no longer required HGF stimulation, i.e., the restriction point (fig. 1). Next, the authors make a differential equations model and fit their time course curves. However, nothing related to the modeling informs any further experimentation. It is simply assumed that the fitting procedure gives and informative model and calculations proceed from there. Therefore, the conclusions from figures 5 and 6 can only be viewed as highly tentative. The main issue I have with this manuscript is that the main conclusions are essentially drawn from a correlation from a single time-course experiment. No manipulations are performed to test hypotheses, which gives the feeling that this a work half finished. Provocatively, the authors main claim is that CAK phosphorylation on T160 of Cdk2 is the rate-limiting step for the R-point. Yet, nothing is done to test this hypothesis. For this manuscript to be suitable for publication in MSB, the authors should endeavor to test this hypothesis (or another non-obvious hypothesis) much more fully. A single correlative piece of evidence is not sufficient.

Re-submission	01 August 2014

Point-by-point answers to reviewer comments

Reviewer #1:

In this study, the authors attempt to quantitatively examine the expression of key cell cycle proteins and during the process of HGF-mediated rat hepatocyte proliferation in culture. One of the interesting features of the study is that they attempt to quantify the abundance of cell cycle proteins and cyclin/cdk/inhibitor complexes at different stages after mitogen stimulation. They then use mathematical models (which are out of my field of expertise) to try to decipher key events. Using these models, they deduce that Thr-160 phosphorylation of cdk2 is "a gate-keeping mechanism contributing to the maintenance of hepatocyte quiescence and the prevention of malignancies."

Comments include the following:

1. While the premises are interesting, nothing in this study examines whether cdk2 phosphorylation is related to malignancy, and I wonder why they include this in the abstract.

We agree with the reviewer that the connection of CDK2 phosphorylation with malignancies is not the scope of the presented work. Therefore, we removed this statement from the abstract.
Abstract, Page 2: “In conclusion, we identify CDK2 phosphorylation as a gate-keeping mechanism contributing to the maintenance of hepatocyte quiescence in the absence of HGF.”

2. Their data show that phosphorylation of cdk2 at this site correlates with events in the G1/S transition, as have been shown previously in hepatocytes and other systems. However, they have obviously not demonstrated experimentally that this is an important event (although I suspect it is).

We agree with the reviewer that experimental validation of our model-based insights was missing. Our model analyses predicted a ranking of the G1/S transition components upon dose-response experiments, with pCDK2 T160 requiring the highest concentration of HGF for activation. To

GERMAN
CANCER RESEARCH CENTER
IN THE HELMHOLTZ ASSOCIATION

50 Years – Research for
A Life Without Cancer

verify this model prediction, we stimulated primary mouse hepatocytes with HGF concentrations ranging from 0.01 to 400 ng/ml and measured the G1/S transition components after 24 h. In line with our model predictions, the calculation of EC_{50} values revealed that pCDK2 T160 ranked as the G1/S transition component with the highest requirement for HGF and thus verified the role of CDK2 phosphorylated at T160 as a gatekeeper. The results are depicted in the new Fig. 7 and the immunoblots are shown in the new Supplementary Fig. S12. The results are summarized in the main text of the manuscript:
Results, Page 16: "To experimentally validate these model predictions, we performed dose-response experiments with HGF. We focused on the G1/S transition components that were addressable by robust measurement, i.e. the complexes of p21:CDK2 and Cyclin D1:CDK4 as well as the phospho-species pRb 788, pRb S800/S804 and pCDK2 T160. Figure 7A–E displays the model-predicted dose-response curves for these components assuming 24 h stimulation with HGF at concentrations ranging from 0.01 to 400 ng/ml indicating the EC_{50} as shown in Fig 6L. The corresponding EC_{50} values for these five components (Fig 7F) suggest a specific ranking with pCDK2 T160 requiring the highest HGF concentration. To experimentally verify our model predictions, we quantified these G1/S transition components by quantitative immunoblotting in primary mouse hepatocytes stimulated with HGF concentrations ranging from 0.04 to 400 ng/ml for 24 h (Supplementary Figure S12). For each averaged data set, we estimated a four-parameter Hill function that was calculated by performing a nonlinear regression of signal intensity as a function of HGF concentration (Figs 7G–K). Interestingly, in line with our model predictions, pCDK2 T160 is indeed the cell cycle component with the highest EC_{50} value (around 30 ng/ml HGF), confirming its role as gatekeeper for HGF-induced DNA synthesis in primary hepatocytes."
Discussion, Page 23: "Importantly, we validated this model prediction experimentally. As predicted, pCDK2 T160 was the G1/S transition component with the highest EC_{50} concerning HGF sensitivity. In general, the experimentally determined EC_{50} values of all analyzed G1/S transition components were approximately one order of magnitude higher than predicted. This might be due to the fact that we assumed a linear relationship between intracellular signaling, cell cycle regulation and DNA synthesis. Nevertheless, the ranking of the experimentally derived EC_{50} values was in line with our model predictions. Interestingly, the average EC_{50} value of pCDK2 was around 30 ng/ml, which is close to the HGF concentration employed in our time-course experiments that result in robust activation of intracellular signaling and proliferation of primary mouse hepatocytes (40 ng/ml HGF). Thus, the properties of pCDK2 T160 determine a threshold to safeguard HGF-controlled proliferation in hepatocytes."

3. A number of older studies have indicated that cyclin D1 is the rate limiting step in the progression of hepatocytes through the G1 restriction point and commits the cell to replication. In the current study, the authors suggest that cdk2 phosphorylation is a more important event. It would be useful for the authors to discuss this in light of prior data regarding cyclin D1.

GERMAN
CANCER RESEARCH CENTER
IN THE HELMHOLTZ ASSOCIATION

50 Years – Research for
A Life Without Cancer

Presumably the reviewer refers to the work of Albrecht and Hansen (Albrecht and Hansen (1999) Cell Growth Differ 10: 397-404) who suggested that Cyclin D1 expression is sufficient to promote progression through G1 in rat hepatocytes. Wierod et al. (Wierod et al. (2008) Oncogene 27: 2763-2771) reported that p21 expression is required for EGF-induced DNA synthesis in rat hepatocytes. The requirement for Cyclin D1 is in line with our results as CDK2 phosphorylation is a downstream process of both Cyclin D1:CDK4 activation and p21-mediated nuclear import of CDK2. We now discuss this point in the main text:

Discussion, Page 23: "It has previously been suggested (Albrecht & Hansen, 1999) that Cyclin D1 expression is sufficient to promote progression through G1 in rat hepatocytes. On the other hand, Wierod et al. (Wierod et al, 2008) reported that p21 expression is required for EGF-induced DNA synthesis in rat hepatocytes. Both results are in agreement with our findings of pCDK2 T160 as a gatekeeper, as CDK2 phosphorylation is a downstream process of both Cyclin D1:CDK4 activation and p21-mediated nuclear import of CDK2."

> *4. The fact that hepatocyte isolation triggers expression of early cell cycle genes has been well documented in the past. The authors should acknowledge this prior body of work.*

As suggested by the reviewer, we now acknowledge this prior body of work:

Results, Page 12: "Since it has been shown that stress induced by the isolation procedure and plating of the cells can activate signaling pathways including the PI3K/Akt and the Raf/MEK/ERK pathway (Fausto et al, 1986; Loyer et al, 1996) that may contribute to a growth factor independent increase in G1/S transition components, we determined the basal activation of these pathways"

> *5. The authors have not shown that "p21 rather than p27 contributed to Cyclin D1:CDK4 complex assembly." Although p21 is binds to the complex, it is not clear from these studies that p21 plays a role in assembly of D1/K4 complexes. In fact, after PH in p21-/- mice, cyclin D1:CDK4 complex activation occurs earlier than in wild-type mice, so p21 is clearly not necessary for this.*

We agree with the reviewer that we did not demonstrate that p21 rather than p27 contributes to Cyclin D1:CDK4 complex assembly. We only show that HGF treatment of primary mouse hepatocytes induces regulation of p21 rather than p27. We adapted the text in the manuscript accordingly:

Abstract, Page 2: "In response to HGF Cyclin:CDK complex formation was increased, p21 rather than p27 was regulated, and Rb expression was enhanced."

Results, Page 7: "In addition, the dynamics of p21:CDK4 were similar to the dynamics of Cyclin D1:CDK4. Unstimulated hepatocytes showed only a minor increase in Cyclin D1:CDK4 over time. Interestingly, CDK2-containg complexes showed a rather unexpected behavior. CDK2-bound Cyclin E (Fig 2B) displayed only a small increase upon HGF stimulation and CDK2-bound p21 (Fig 2J) remained constant. CDK2-bound p27 (Fig 2C) increased slightly over time, independent of HGF stimulation."

dkfz. GERMAN CANCER RESEARCH CENTER IN THE HELMHOLTZ ASSOCIATION

50 Years – Research for A Life Without Cancer

6. Obviously, it is critical that the authors have high-quality antibodies and western blots in order to do their quantitative analysis. It would be helpful for them to demonstrate the quality of their antibodies for key proteins (e.g. cyclin E1) by doing controls with blocking peptides or knockdown.

In line with the reviewer's suggestions, we performed control experiments to confirm the specificity of our antibodies and show the results in the new Supplementary Figure S3. We stimulated primary mouse hepatocytes with 40 ng/ml HGF for 48 h and performed immunoprecipitation experiments. For CDK2, CDK4 and Cyclin E blocking peptides were available from the manufacturer and utilized in the competition studies. As depicted in Fig. S3A addition of blocking peptides results in strong reduction of the specific signal for CDK2, CDK4 and Cyclin E. For the other antibodies (Cyclin D1, Rb and p21), we performed competition experiments with recombinant proteins as shown in Fig. S3B. Incubating the protein lysates with competing amounts of recombinant proteins (50 to 100 fold compared to the endogenous protein) prior to immunoprecipitation results in the reduction of the specific signal, while unspecific bands remain unaffected. In summary, our new experiments confirm the high quality and specificity of our antibodies.

These new experiments are described in the materials and methods as well as in the main text:

Results, Page 7: "By performing control experiments using blocking peptides or competing amounts of recombinant proteins (Supplementary Figure S3), we confirmed the specificity and the quality of our antibodies."

7. The assumptions used in their models may or may not be valid in hepatocytes.

The reviewer raises an important point. We now explicitly state our assumptions in the new paragraph on model assumptions in the main text and also discuss hepatocyte-specific G1/S transition regulation mechanisms that are supported by our experiments. To accommodate this paragraph, we moved the general model description to the Supplementary Information. The fact that our mathematical model reproduces the data obtained with primary mouse hepatocytes indicates consistency of our assumptions with our observations, as now stated in our manuscript:

Results, Page 13: "Thus, our model can explain the experimental data, indicating that our model assumptions are consistent with the regulation of G1/S transition in primary mouse hepatocytes."

Minor issues:

1. First line of abstract: Many factors contribute to liver regeneration, and I'm not sure that "Liver regeneration is ... primarily achieved by hepatocyte growth factor (HGF)-induced proliferation."

We agree with the reviewer that many factors contribute to liver regeneration. Therefore we rephrased the first lines of the abstract: "Liver regeneration is a tightly controlled process achieved by proliferation of usually quiescent hepatocytes. However, the specific molecular mechanisms ensuring cell division only in response to proliferative signals such as hepatocyte growth factor (HGF) are not fully understood."

dkfz. GERMAN CANCER RESEARCH CENTER IN THE HELMHOLTZ ASSOCIATION

50 Years – Research for A Life Without Cancer

2. The authors should note that older studies have studies the mitogen restriction point in rat hepatocytes (1, 2), and was determined to be at similar time points after plating the cells.

We thank the reviewer for pointing out these older studies. Because references 1,2 have not been transmitted to us, we have carefully evaluated previously published reports concerning the restriction point of *ex vivo* cultivated primary hepatocytes. Albrecht and Hansen (Albrecht and Hansen (1999) Cell Growth Differ 10(6):397-404) isolated primary rat hepatocytes and cultivated the cells in presence of EGF. Their data indicates a restriction point at 40 to 44 h after cultivation. With a similar experimental setting, Loyer et al. (Loyer et al (1996) J Biol Chem 271(19):11484-92) have determined the restriction point in rat hepatocytes at 42 to 48 h post seeding. Please note that the cultivation procedure in these two reports is different from our experimental setting, as they did not include an adhesion and growth factor depletion phase. We determined the restriction point at 32 h after HGF stimulation, which corresponds to 62 h total cultivation time. In a review on liver regeneration (Michalopoulos (2007) J Cell Physiol 213(2):286-300), it was stated that the peak of the DNA synthesis is faster in rat than in mouse hepatocytes, with a shift of 6–12 h. If we assume that the same is true for the restriction point, our results are indeed in accordance with these reports. We now discuss this in our manuscript:
Discussion, Page 18: "It has previously been reported that the peak of DNA synthesis is faster in rat than in mouse hepatocytes with a shift of 6–12 h (Michalopoulos, 2007). Primary rat hepatocytes have been shown to cross the restriction point after 40–44 h of growth factor stimulation (Albrecht & Hansen, 1999; Loyer et al, 1996). However, the experimental procedure in these two studies did not include an adhesion and growth factor depletion phase, but rather placed the cells directly in growth factor-supplemented medium following isolation. We determined the restriction point in primary mouse hepatocytes at 32 h after HGF stimulation, which corresponds to a total cultivation time of 62 h including adhesion, growth factor depletion, and stimulation phases. Thus, we conclude that similar to the timing of DNA synthesis, the timing of the restriction point is faster in rat than in mouse hepatocytes."

Reviewer #2:

Summary:

This study aims to identify molecular components that regulate DNA replication during the cell cycle in hepatocytes. An understanding of how the cell cycle is controlled could help reveal why these cells divide in response to specific cues, and how uncontrolled division is avoided. To address these questions, the authors performed quantitative and semi-quantitative experimental measurements of cell cycle readouts in primary mouse hepatocytes. These measurements were used to parameterize a computational model that consisted of two parts: a logical model describing inputs, and a chemical kinetic model describing dynamics of cell cycle components. A key conclusion extracted from the model is that phosphorylation of T160 in CDK2 must cross a threshold level for DNA replication to be induced.

General remarks:

The study could be of interest to those involved in quantitative investigation of the cell cycle. The modeling aspect improves on a previous study of the system that relied on logical modeling only. The experimental measurements seem valuable, covering a fairly large number of readouts at multiple timepoints in the presence and absence of HGF.

We thank the reviewer for the encouraging comments.

However, the primary use of experimental data seems to have been in estimating parameters in the model. Model predictions arising from the proposed mechanisms were not tested much, if at all. As a result, we are not fully convinced of the major conclusion, i.e. the role of CDK2 T160 as a gatekeeper. Can the authors perform tests of model predictions to strengthen their conclusions?

We have followed the advice of the reviewer and performed validation experiments of the model predictions, verifying the role of CDK2 phosphorylated at T160 as a gatekeeper. The results are depicted in the new Fig. 7 and the immunoblots are shown in the new Supplementary Figure S12. We summarize our results in the main text of the manuscript:
Results, Page 16: "To experimentally validate these model predictions, we performed dose-response experiments with HGF. We focused on the G1/S transition components that were addressable by robust measurement, i.e. the complexes of p21:CDK2 and Cyclin D1:CDK4 as well as the phospho-species pRb 788, pRb S800/S804 and pCDK2 T160. Figure 7A–E displays the model-predicted dose-response curves for these components assuming 24 h stimulation with HGF at concentrations ranging from 0.01 to 400 ng/ml indicating the EC_{50} as shown in Fig 6L. The corresponding EC_{50} values for these five components (Fig 7F) suggest a specific ranking with pCDK2 T160 requiring the highest HGF concentration. To experimentally verify our model predictions, we quantified these G1/S transition components by quantitative immunoblotting in primary mouse hepatocytes stimulated with HGF concentrations ranging from 0.04 to 400 ng/ml for 24 h (Supplementary Figure S12). For each averaged data set, we estimated a four-parameter Hill function that was calculated by performing a nonlinear regression of signal intensity as a function of HGF concentration (Figs 7G–K). Interestingly, in line with our model predictions, pCDK2 T160 is indeed the cell cycle component with the highest EC_{50} value (around 30 ng/ml HGF), confirming its role as gatekeeper for HGF-induced DNA synthesis in primary hepatocytes."
Discussion, Page 23: "Importantly, we validated this model prediction experimentally. As predicted, pCDK2 T160 was the G1/S transition component with the highest EC_{50} concerning HGF sensitivity. In general, the experimentally determined EC_{50} values of all analyzed G1/S transition components were approximately one order of magnitude higher than predicted. This might be due to the fact that we assumed a linear relationship between intracellular signaling, cell cycle regulation and DNA synthesis. Nevertheless, the ranking of the experimentally derived EC_{50} values was in line with our model predictions. Interestingly, the average EC_{50} value of pCDK2 was around 30 ng/ml, which is close to the HGF concentration employed in our time-course experiments that result in

GERMAN CANCER RESEARCH CENTER
IN THE HELMHOLTZ ASSOCIATION

50 Years – Research for
A Life Without Cancer

robust activation of intracellular signaling and proliferation of primary mouse hepatocytes (40 ng/ml HGF). Thus, the properties of pCDK2 T160 determine a threshold to safeguard HGF-controlled proliferation in hepatocytes."

Major points:

1) In some cases, differences between experimental readouts seem to be overstated. For example, it is said that expression of CDK4 remained constant over time while CDK2 increased slightly. From looking at the plots in Fig 2A, it seems that both proteins increased slightly in the HGF-stimulated cells, following an initial drop.

We agree with the reviewer. We have rephrased the paragraph in the main text, stating now that both proteins increased slightly in the HGF-stimulated cells, following an initial drop.
Results, Page 7: "Subsequent to a minor initial drop, the expression of CDK4 (Fig 2E) and CDK2 (Fig 2A) increased slightly in stimulated hepatocytes, while they appeared rather constant in untreated hepatocytes."

2) Similarly, it is said that CDK2-bound p27 remained constant. The figure indicates that CDK2-bound p27 increases over time, with the stimulated and unstimulated cells looking much the same.

Again, we adapted the interpretation of our experimental finding and state that CDK2-bound p27 increases slightly over time, independent of HGF stimulation:
Results, Page 8: "CDK2-bound p27 (Fig 2C) increased slightly over time, independent of HGF stimulation."

*3) As a result of these ambiguities, the meaning of the following sentence is not evident: "The results allowed us to define the following regulated G1/S transition components in primary mouse hepatocytes..." It is unclear how this conclusion was reached. What is the feature shared by all of the components listed? Is this feature *not* shared by the components omitted? For example, why is p21:CDK4 not included?*

We apologize for not making this point clear. The complex p21:CDK4 is indeed induced by HGF. It has previously been reported (Cheng et al, (1999) EMBO J 18, 1571-1583) that the complex of Cyclin D1 with CDK4 is unstable without CIP/KIP proteins. As Cyclin D1:CDK4 and p21:CDK4 showed very comparable dynamics in our experiments (Fig 2F,G), we assumed that these proteins are primarily associated in the trimeric complex Cyclin D1:CDK4:p21. To prevent adding too much weight on the same observation, we decided to use only the observation of Cyclin D1:CDK4 for parameter estimation. We specify this point in the new paragraph on model assumptions (Page 10). We now also define our criteria for the selection of G1/S transition components in the main text:
Results, Page 8: "For our further analyses, we selected total proteins or phosphorylated proteins as G1/S transition components based on the following criteria: The component is regulated during the observed time frame and the regulation is substantially different if cells are stimulated with 40 ng/ml HGF compared to the untreated condition. Additionally, we

GERMAN
CANCER RESEARCH CENTER
IN THE HELMHOLTZ ASSOCIATION

50 Years – Research for
A Life Without Cancer

selected Cyclin D1:CDK4 as representative of the trimeric complex Cyclin D1:CDK4:p21 to avoid redundant information."

> *4) The model diagram in Fig 3B is not easily interpreted (what do the dots on lines mean?), and is not connected to the underlying model in a clear way. Since this diagram represents a rule-based model, the authors may consider taking advantage of published, standardized guidelines for visualizing such models. Arrows in the diagram could be cross-referenced with numbered rules, which seem to already be part of the supplement. These changes would go a long way towards communicating the model's content to a reader.*

We followed the recommendations of the reviewer and completely revised Fig. 3. We have re-designed the model network following the representation guidelines of Systems Biology Graphical Notation (SBGN) as reported by Kitano and colleagues (Nat Biotechnol. (2009) 27(8):735-41). Furthermore, we realized the very constructive suggestion to cross-reference the arrows in the diagram. Every edge in Fig. 3B is now labeled with the number of the corresponding rule explained in detail in the Supplementary Information.

> *5) The authors state that they measured basal "activity" of Akt and ERK. What they seem to have actually measured is phosphorylation level of these two proteins. How was phosphorylation level translated into enzymatic activity (if that is what was done) and used in the model?*

The reviewer is correct in pointing out that we determined the phosphorylation levels rather than the enzymatic activity of Akt and ERK. We have determined the amount of murine ERK1 doubly-phosphorylated on Thr 203 and Tyr 205 (Thr 183 and Tyr 185 in ERK2), which has previously been shown to correspond to enzymatically active ERK (Boulton et al. (1991) Cell 65, 663-675; Robbins et al. (1993) J. Biol. Chem. 268, 5097-5106). We have confirmed the specificity of the polyclonal antibodies employed in this study to monitor double-phosphorylated ERK by using residue-specific monoclonal antibodies (Schilling et al. (2009). Mol Syst Biol.5:334). For Akt, we measured phosphorylation on Ser 473, which is important for complete activation of Akt (Sarbassov et al. (2005) Science 307(5712):1098-101). This information is included in the main text of the manuscript:
Results, Page 12: "Furthermore, we quantified in HGF-stimulated primary mouse hepatocytes the phosphorylation of Akt on Ser 473 required for complete activation of Akt (Sarbassov et al, 2005), and the double phosphorylation of ERK (Thr 203 and Tyr 205 in murine ERK1; Thr 183 and Tyr 185 in murine ERK2) corresponding to active ERK (Boulton et al, 1991; Robbins et al, 1993)."

> *6) A number of observables are selected without much explanation, and are plotted in Fig S6. In the discussion of this figure in the text, it is stated that "we indeed observed their temporal behavior in the absence and presence of HGF, validating the predictive power of our model". We do not see how one draws conclusions about predictive power (the ability to make testable predictions) based on the fact that a model can simply generate timecourses. Are the molecules represented here particularly amenable to experimental measurement? We would expect not, since many of them refer to specific phosphoforms and bound species.*

We apologize for not sufficiently explaining why we included Fig. S6. A mathematical model consists of many species, most of which are not observable as they are not directly amenable to experimental measurements. For example, we measured phosphorylation of CDK2 on Thr 160, which corresponds to the sum of Cyclin E:pCDK2 and Cyclin E:pCDK2:p21. Frequently, model parameters cannot be uniquely determined based on the experimental data and thus are non-identifiable. Therefore, while the dynamics of the observable species such as the sum of the two complexes is described, the individual model species such as the dynamics of the two separate complexes separately is not predicted by the model. To analyze if this applies to our modeling approach, we plotted all the model species for ten different parameter sets. As most of the species display similar dynamics for all these ten parameter sets, we conclude that all best fits provide consistent dynamics for the species we did not experimentally measure, which is a prerequisite for the model's ability to make testable predictions. However, we do agree that these observations do not imply that our model predicts correctly new experiments and therefore, we removed the sentence "… validating the predictive power of our model". We now explain this in the manuscript:
Results, Page 14: "For example, a protein might participate in additional complexes or become phosphorylated at different sites. Therefore, even if a model can uniquely describe the observed species, other model species that are not amenable to experimental measurements may not be correctly described. If this was the case, the dynamics of the non-observed model species would be very different for each parameter set. To verify if our model is able to uniquely describe the non-observed model species, we plotted the time-resolved dynamics of the internal model variables (Supplementary Figure S7B). For several variables we could observe similar temporal dynamics in the absence and presence of HGF for the ten best parameter sets. This indicates that our model can provide information about species that are not directly experimentally addressable."

> *7) Although the proposed importance of CDK2 T160 is made clear, the underlying mechanisms that bring about this role are not. Why does it rely so much more strongly on HGF than the other readouts? Are there conditions under which it would lose its strong dependence on HGF? Considering these types of questions could help develop the model's actual predictive power.*

The reviewer raises here an interesting point. It is indeed tempting to speculate why the T loop phosphorylation of CDK2 is much more dependent on HGF stimulation than the other species. Activation of CDK2 is a very complex process. In our model we have included CDK2 regulatory mechanisms that that were previously reported or indicated by our experiments. Complex feedback mechanisms involving p21 and E2F-1 result in a highly non-linear CDK2 activation. While it does not exclude other mechanisms, the feedback loops in our model are sufficient to explain the model-predicted and experimentally confirmed gatekeeper function of pCDK2 T160. We pose the following hypotheses: While p21 is an activator of Cyclin D1:CDK4, it has a dual role with respect to Cyclin E:CDK2, being both activator and inhibitor. Therefore, because Cyclin E:CDK2 depends in a non-linear manner on p21 concentrations,

GERMAN
CANCER RESEARCH CENTER
IN THE HELMHOLTZ ASSOCIATION

50 Years – Research for
A Life Without Cancer

particularly high HGF concentrations might be required for full CDK2 activation. Furthermore, the Cyclin E:CDK2 complex is part of feedback loops that similarly might require high HGF concentration to be triggered, contrary to Cyclin D1:CDK4, whose activation depends rather linearly on HGF. We have included these explanations into the discussion of our manuscript:
Discussion, Page 23: "It is tempting to speculate why the T loop phosphorylation of CDK2 is more dependent on HGF stimulation than the other species. We have implemented regulatory mechanisms in our model that resulted in a highly non-linear CDK2 activation. While p21 is an activator of Cyclin D1:CDK4, it has a dual role with respect to Cyclin E:CDK2, being both activator and inhibitor. Consequently, because Cyclin E:CDK2 depends in a non-linear manner on p21 concentrations, we hypothesize that particularly high HGF concentrations might be required for full CDK2 activation. While the Cyclin E:CDK2 complex is part of non-linear regulatory mechanisms requiring high HGF concentration to be triggered, the activation of Cyclin D1:CDK4 depends rather linearly on the HGF concentration. While it does not exclude other mechanisms, the regulatory mechanisms in our model are sufficient to explain the predicted gatekeeper function of pCDK2 T160."

8) Finally, why was it necessary to use logical modeling? Is it not possible to represent HGF's impact in terms of chemical kinetics?

Yes, we have indeed represented HGF's impact in terms of chemical kinetics in a simplified input model. The dependencies between the model variables are based on our previously published hepatocyte-specific logical model (Huard et al. (2012) FEBS J 279(18):3290-313). In contrast to this logical model, our input model consists of variables that can adapt any value between 0 (no activity) and 1 (maximum activity). Additionally, we neglected the time-dependency of the input variables for simplification. To clarify this point, we now only use the term "simplified input model" in the text.

Minor points:

1) For a broad audience, it would be helpful if the more specialized terms (e.g., resection, T loop) were defined.

We have defined all specialized terms in the main text as suggested, including "resection", "partial hepatectomy" and "T loop".

2) In Fig 2 and other similar figures that contain many plots, it would be helpful if the plots were labeled individually (A, B, C, etc.) and referenced in the text. That would make it easier for a reader to quickly go between the data and the discussion.

We have re-labeled all subplots for easier reference. This affects Fig. 2, Fig. 4 and Fig. 6.

3) A typo on page 12, 2nd paragraph: "while the G1/S transition components where low" should be "were".

The typo was corrected.

4) In the caption of Fig 2A, the meaning of the vertical dashed lines should be stated. I assume it corresponds to the estimated restriction point.

GERMAN
CANCER RESEARCH CENTER
IN THE HELMHOLTZ ASSOCIATION

50 Years – Research for
A Life Without Cancer

Yes, the vertical dashed lines correspond to the estimated restriction point. We have added this information to the figure captions of Fig. 2A–N and Fig. 4A–I.

5) Although the model is described in the main text, it reads more like a review and does not say much about how the information was translated into a model, what assumptions were used, etc. A reader may refer to details in the supplement, but a more concrete description in the text itself would be useful.

We thank the reviewer for the idea to focus on the model assumptions in the main text of the manuscript. We now explicitly state our assumptions in the new paragraph on model assumptions in the main text and also discuss hepatocyte-specific G1/S transition regulation mechanisms. We also explain how these assumptions are supported by our experimental data obtained with primary mouse hepatocytes. To accommodate this paragraph, we moved the general model description to the Supplementary Information. The fact that our mathematical model reproduces the primary mouse hepatocyte data indicates consistency of our assumptions with the experimental observations, as stated in our manuscript:
Results, Page 13: "Thus, our model can explain the experimental data, indicating that our model assumptions are consistent with the regulation of G1/S transition in primary mouse hepatocytes."

6) Table S5, the logical network, is in need of some explanation of what the variables are meant to represent.

We have now included the following explanation in the caption of this table featuring the equations of the simplified input model: "Signal transduction network controlled by HGF. These equations describe the level of activity reached for the input variables erk, akt, gsk3b, tf and tfp21 at steady state for a given HGF concentration. HGF, inherk, inhakt and inhp53 are inputs that are set according to the experiment (inherk, inhakt and inhp53 are set to 1 in case of treatment with the MEK inhibitor U0126, the Akt Inhibitor VIII and the p53 inhibitor Pifithrin, respectively)."

Reviewer #3:

Review of the manuscript 'T160-phosphorylated CDK2 defines the threshold for HGF-dependent proliferation in primary hepatocytes' by Mueller et al for MSB. The authors perform a nice time-course experiment of primary hepatocytes exposed to HGF, which induces them to enter the cell cycle. The authors track over 10 distinct protein species quantitatively with dense temporal sampling in both the experiment (HGF induced) and the control (fig. 2). This is a nice experiment since the hepatocytes are relatively well-synchronized and such comprehensive time courses are generally lacking for primary cells. The authors also show that Cdk4, Akt, Mek and p53 inhibitors are required for S-phase in their system (fig. 4b). Also, the authors perform a nice experiment to determine where in this time course the majority of cells are committed to division and no longer required HGF stimulation, i.e., the restriction point (fig. 1).

We thank the reviewer for the encouraging comments.

Next, the authors make a differential equations model and fit their time course curves. However, nothing related to the modeling informs any further experimentation. It is simply assumed that the fitting procedure gives and informative model and calculations proceed from there. Therefore, the conclusions from figures 5 and 6 can only be viewed as highly tentative. The main issue I have with this manuscript is that the main conclusions are essentially drawn from a correlation from a single time-course experiment. No manipulations are performed to test hypotheses, which gives the feeling that this a work half finished. Provocatively, the authors main claim is that CAK phosphorylation on T160 of Cdk2 is the rate-limiting step for the R-point. Yet, nothing is done to test this hypothesis. For this manuscript to be suitable for publication in MSB, the authors should endeavor to test this hypothesis (or another non-obvious hypothesis) much more fully. A single correlative piece of evidence is not sufficient.

The reviewer raises an important point. Inspired by the comments and suggestions of the reviewers, we have performed validation experiments of the model predictions, verifying the role of CDK2 phosphorylated at T160 as a gatekeeper. The results are depicted in the new Fig. 7 and the immunoblots are shown in the new Supplementary Figure S12. We summarize our results in the main text of the manuscript:

Results, Page 16: “To experimentally validate these model predictions, we performed dose-response experiments with HGF. We focused on the G1/S transition components that were addressable by robust measurement, i.e. the complexes of p21:CDK2 and Cyclin D1:CDK4 as well as the phospho-species pRb 788, pRb S800/S804 and pCDK2 T160. Figure 7A–E displays the model-predicted dose-response curves for these components assuming 24 h stimulation with HGF at concentrations ranging from 0.01 to 400 ng/ml indicating the EC_{50} as shown in Fig 6L. The corresponding EC_{50} values for these five components (Fig 7F) suggest a specific ranking with pCDK2 T160 requiring the highest HGF concentration. To experimentally verify our model predictions, we quantified these G1/S transition components by quantitative immunoblotting in primary mouse hepatocytes stimulated with HGF concentrations ranging from 0.04 to 400 ng/ml for 24 h (Supplementary Figure S12). For each averaged data set, we estimated a four-parameter Hill function that was calculated by performing a nonlinear regression of signal intensity as a function of HGF concentration (Figs 7G–K). Interestingly, in line with our model predictions, pCDK2 T160 is indeed the cell cycle component with the highest EC_{50} value (around 30 ng/ml HGF), confirming its role as gatekeeper for HGF-induced DNA synthesis in primary hepatocytes.”

Discussion, Page 23: “Importantly, we validated this model prediction experimentally. As predicted, pCDK2 T160 was the G1/S transition component with the highest EC_{50} concerning HGF sensitivity. In general, the experimentally determined EC_{50} values of all analyzed G1/S transition components were approximately one order of magnitude higher than predicted. This might be due to the fact that we assumed a linear relationship between intracellular signaling, cell cycle regulation and DNA synthesis. Nevertheless, the ranking of the experimentally derived EC_{50} values was in line with our model predictions. Interestingly, the average

EC_{50} value of pCDK2 was around 30 ng/ml, which is close to the HGF concentration employed in our time-course experiments that result in robust activation of intracellular signaling and proliferation of primary mouse hepatocytes (40 ng/ml HGF). Thus, the properties of pCDK2 T160 determine a threshold to safeguard HGF-controlled proliferation in hepatocytes.”

2nd Editorial Decision 11 September 2014

Thank you again for submitting your work to Molecular Systems Biology. We have now heard back from the two referees who agreed to evaluate your study. The previous reviewer #2 (current reviewer #1) has reviewed the revised manuscript. This reviewer is a computational expert and was specifically asked (both this time and during the review of the initial submission) to focus on evaluating the modeling aspect of the study. Since the previous reviewer #1 was not available this time, we invited a new reviewer (#2), who was asked to evaluate the study afresh, with a focus on the biological significance of the main findings.

As you will see from the reports below, reviewer #2 raises substantial concerns on your work, which, I am afraid to say, preclude its publication in Molecular Systems Biology.

In particular, reviewer #2 is not convinced that the main conclusion, namely that CDK2 T160 phosphorylation sets a threshold for hepatocyte proliferation, is robustly supported by the data. Moreover, this reviewer points out that the analysis of population-level effects without considering cell-to-cell heterogeneity, does not seem to provide conclusive insights into the mechanisms underlying decision making at the single cell level, thus raising a fundamental issue that potentially undermines the overall significance of the main findings.

Under these circumstances and considering the low level of support provided by reviewer #2, I see no other choice than to return the manuscript with the message that we cannot offer to publish it. In any case, thank you for the opportunity to examine your work. I hope that the points raised in the reports will prove useful to you and that you will not be discouraged from submitting future work to Molecular Systems Biology.

Reviewer #1:

The authors have thoroughly addressed the concerns we expressed about the original manuscript. The manuscript has been improved.

Reviewer #2:

This manuscript presents a combined quantitative immunoblot and model analysis of primary hepatocyte proliferation. The authors seek to extend previous generic models of the G1-S transition to the primary hepatocytes to shed light on the biologically important process of liver regeneration. To do this, they perform a quantitative analysis of the induction of DNA proliferation and associated molecular events in culture, and then use these data to fit a computational model of the process. One surprising aspect of this analysis is that phosphorylation of CDK2 at T160 appears to have an inflection point at a significantly higher level than other events involved in the process, and the authors conclude that this is a "gatekeeper" mechanism that ensures that both of the upstream signaling pathways, Ras/ERK and PI3K/Akt, must be active to stimulate the decision to enter S-phase. There are several very nice things about this study: the use of isolated primary cells is an appropriate and relevant system, the experiments have been designed carefully, and the immunoblots have been carried out very nicely with controls that allow absolute quantitation. The model appears to be well constructed and appropriately analyzed, and the data are presented clearly. This study would likely be of interest to others studying the G1-S transition, and the data set would likely serve as a useful resource for this field.

However, there is a very substantial mismatch between the data/model analysis and the main conclusion, which is that phosphorylation of CDK2 T160 sets a threshold for the entry into S-phase. There are several issues with the reasoning behind this main conclusion:
- The term "threshold" implies a distinct value at which an event is triggered, but none of the data presented in the paper seem to indicate such a characteristic. All of the data shown are shallow sigmoidal curves that have Hill values near 1. The use this term to refer to the midpoints of such curves is very confusing - the graded nature of the data suggest the opposite of a true threshold.

© European Molecular Biology Organization

- The main argument for why pT160 constitutes a threshold is not stated very clearly in a quantitative way. The argument seems to be that because a) pT160 is required for full activity of CDK2 and b) pT160 has an EC50 higher than other G1-S components, it must be the limiting factor. But this logic glosses over many of the gray areas in the data and attempts to cast them as black and white. For example, DNA synthesis can be detected as low as 0.5 ng/ml HGF (Fig. 4K), but pT160 does not appear to increase significantly until >10 ng/ml (Fig. 7K). One could argue that only a barely detectable level of pT160 is necessary to induce DNA synthesis, but this would be inconsistent with Fig. 2D/4B where a detectable increase in pT160 (relative to unstimulated cells) precedes DNA synthesis by about 24 hours. It is not clear how all of these curves fit together into a consistent model with a simple threshold mechanism.
- The authors point out that the shallowness of the curve in 1B is likely the result of population heterogeneity, which seems to be a likely explanation. But if the kinetics they observe by immunoblot really are the convolution of a complex mixture of cells triggering DNA proliferation at different thresholds or different times, then the population data and model they present are not very informative of how individual cells make decisions.
- A very relevant recent study using a single-cell reporter of CDK2 (PMID: 24075009) has been overlooked. This study shows that the initial induction of CDK2 activity follows a multi-phasic trajectory with cells entering S-phase at the end of the first stage of CDK2 activity (which begins at different times in each cell). Given that similar complexity likely underlies the proliferation decision in this system, it is not clear what can be resolved with an immunoblot approach.
- The model appears to treat DNA synthesis as a continuously variable process, which is appropriate if the model is meant to represent the population of cells. But the argument of the paper is about a discrete event - entry into S-phase vs. maintenance of G1- that occurs at the single cell level, and the relationship between these two views is never made clear.

In short, population-level effects (monitored by immunoblot, generally very graded) and cellular-level behavior (presumably discrete, but with cell-to-cell variability) have not been carefully distinguished, which makes for a confusing and less than convincing case for the paper's major conclusion.

Re-submission 13 January 2015

© European Molecular Biology Organization

GERMAN
CANCER RESEARCH CENTER
IN THE HELMHOLTZ ASSOCIATION

50 Years – Research for
A Life Without Cancer

Point-by-point answers to reviewer comments

Reviewer #1:

The authors have thoroughly addressed the concerns we expressed about the original manuscript. The manuscript has been improved.

We thank the reviewer for his/her time and helpful comments.

Reviewer #2:

This manuscript presents a combined quantitative immunoblot and model analysis of primary hepatocyte proliferation. The authors seek to extend previous generic models of the G1-S transition to the primary hepatocytes to shed light on the biologically important process of liver regeneration. To do this, they perform a quantitative analysis of the induction of DNA proliferation and associated molecular events in culture, and then use these data to fit a computational model of the process. One surprising aspect of this analysis is that phosphorylation of CDK2 at T160 appears to have an inflection point at a significantly higher level than other events involved in the process, and the authors conclude that this is a "gatekeeper" mechanism that ensures that both of the upstream signaling pathways, Ras/ERK and PI3K/Akt, must be active to stimulate the decision to enter S-phase. There are several very nice things about this study: the use of isolated primary cells is an appropriate and relevant system, the experiments have been designed carefully, and the immunoblots have been carried out very nicely with controls that allow absolute quantitation. The model appears to be well constructed and appropriately analyzed, and the data are presented clearly. This study would likely be of interest to others studying the G1-S transition, and the data set would likely serve as a useful resource for this field.

We thank the reviewer for the encouraging comments.

However, there is a very substantial mismatch between the data/model analysis and the main conclusion, which is that phosphorylation of CDK2 T160 sets a threshold for the entry into S-phase. There are several issues with the reasoning behind this main conclusion:

- The term "threshold" implies a distinct value at which an event is triggered, but none of the data presented in the paper seem to indicate such a characteristic. All of the data shown are shallow sigmoidal curves that have Hill values near 1. The use this term to refer to the midpoints of such curves is very confusing - the graded nature of the data suggest the opposite of a true threshold.

We apologize that we were not sufficiently clear regarding the use of the term threshold. In line with the definition of this term, G1/S transition in individual hepatocytes must be controlled by a specific threshold at a certain time. However, to gain insights into mechanisms regulating regeneration of the liver, it is equally important to assess the behavior of hepatocytes at the population level. As an explanation for the sigmoidal response observed by biochemical measurements of the population, we assumed that each hepatocyte undergoes G1/S transition at a slightly different time point. To experimentally test this hypothesis, we performed new experiments with Fucci2 mice-derived hepatocytes. The single cell data shown in the new Figure 1F demonstrate that in the absence of HGF all hepatocytes are in G1 phase. Upon stimulation with HGF, time of entry into S phase is slightly different between individual hepatocytes. By averaging the data, we obtained a sigmoidal curve, congruent with our population measurement. We apologize if we conveyed the impression that with the term threshold we refer to the midpoint of the sigmoidal curves. We only use the midpoints of the sigmoidal curves to compare different population data, such as the G1/S transition components and the average time of G1/S transition at the single cell level. By the similar values of the midpoints and the linear correlation of the extent of CDK2 T160 phosphorylation and cycling hepatocytes (see response to next question), we concluded that CDK2 T160 phosphorylation acts as a threshold in single cells. We now define the term threshold and describe the new experiments and results in the results section on page 8 of the main text:

"In single cells, G1/S transition occurs at a distinct time and is controlled by a specific threshold. In this case, the term threshold corresponds to a distinct value (a concentration of a specific signaling molecule) at which an event (G1/S transition) is triggered in a single cell. If in a cell population all hepatocytes underwent G1/S transition at the same time, the DNA content would increase at this time point. On the other hand, if the different hepatocytes in a population underwent G1/S transition at different times after exposure to HGF, the response would be more graded. To experimentally test this hypothesis, we quantified the time of G1/S transition in the Fucci2 hepatocytes. Figure 1F shows the number of hepatocytes that underwent G1/S transition at least once, plotted in a time-resolved manner. The data could be approximated with a sigmoidal function featuring comparable slope and steepness compared to our cell population data. Our data showed that the timing of G1/S transition varies between individual hepatocytes, with an average G1/S transition time corresponding to ca. 33 h. Therefore, we conclude that individual hepatocytes undergo G1/S transition at different times after HGF stimulation, converting a step-like individual decision to a sigmoidal population response."

GERMAN
CANCER RESEARCH CENTER
IN THE HELMHOLTZ ASSOCIATION

50 Years – Research for
A Life Without Cancer

- The main argument for why pT160 constitutes a threshold is not stated very clearly in a quantitative way. The argument seems to be that because a) pT160 is required for full activity of CDK2 and b) pT160 has an EC50 higher than other G1-S components, it must be the limiting factor. But this logic glosses over many of the gray areas in the data and attempts to cast them as black and white. For example, DNA synthesis can be detected as low as 0.5 ng/ml HGF (Fig. 4K), but pT160 does not appear to increase significantly until >10 ng/ml (Fig. 7K). One could argue that only a barely detectable level of pT160 is necessary to induce DNA synthesis, but this would be inconsistent with Fig. 2D/4B where a detectable increase in pT160 (relative to unstimulated cells) precedes DNA synthesis by about 24 hours. It is not clear how all of these curves fit together into a consistent model with a simple threshold mechanism.

To clarify the points raised by the reviewer and strengthen our argument that CDK2 T160 phosphorylation constitutes a threshold for proliferation, we performed new linear regression analyses between G1/S transition components and hepatocytes in S/G2/M phase. Because the dose response curve in Fig. 4K is rather shallow and does not include the response to high concentrations of HGF, we performed new dose response experiments based on single hepatocytes. By analyzing our new single cell data, we quantified the effect of HGF on G1/S transition for a wide concentration range (new Figure 8C,D,E). We demonstrate that the dose-dependency of G1/S transition has an EC_{50} value towards HGF concentrations that is in line with the EC_{50} value to HGF of CDK T160 phosphorylation. Furthermore, we show a linear correlation between CDK2 T160 phosphorylation and hepatocytes nuclei in S/G2/M phase. We summarize our new results in the results section on page 19 of the main text:

"To analyze the response of all hepatocytes to HGF at the single cell level, we segmented cell nuclei of Histone2B-mCerulean-labelled Fucci2 hepatocytes and quantified the Fucci2 markers in a time window from 42 to 54 h post stimulation. We calculated the percentage of cells in the respective cell cycle phases using the same criteria for the Fucci2 cell cycle markers as above. As expected, the percentage of cells in S/G2/M phase was increasing as a function of HGF (Fig 8C and Supplementary Figure S14B). The percentage of cells in S/G2/M phase in this snapshot analysis did not correspond to the total number of proliferating cells, because a cycling cell only remains in S/G2/M phase for a certain period of time, as shown in Figure 8A. To determine the relationship between HGF concentration and the number of cycling cells, we plotted the number of hepatocyte nuclei in S/G2/M phase versus HGF concentration, performed a sigmoidal regression and calculated the EC_{50} (Fig 8D). The EC_{50} of 16 ng/ml HGF was close to the EC_{50} of pCDK2 T160 to HGF (Fig 7K). This prompted us to hypothesize a linear relationship between pCDK2 T160 and induction of DNA synthesis. We therefore performed a linear regression between the experimentally measured phosphorylation levels of CDK2 at T160 (at 48 h post HGF stimulation) and hepatocyte nuclei in S/G2/M (at 42 to 54 h post HGF stimulation) for corresponding HGF concentrations. We indeed observed a highly significant linear relationship between these two experimental measurements. While all G1/S transition components increased with HGF and correlated with G1/S

transition, the correlation with CDK2 T160 phosphorylation had the lowest p-value (Supplementary Table S2), confirming our model predicted importance of pCDK2 T160 as gatekeeper for G1/S transition in hepatocytes."

> *- The authors point out that the shallowness of the curve in 1B is likely the result of population heterogeneity, which seems to be a likely explanation. But if the kinetics they observe by immunoblot really are the convolution of a complex mixture of cells triggering DNA proliferation at different thresholds or different times, then the population data and model they present are not very informative of how individual cells make decisions.*

It is indeed an interesting question what causes the shallowness of the curve in 1B (time-dependent increase in DNA content). We speculated that the primary mouse hepatocytes in our *ex vivo* culture conditions are rather homogenous, but the start of DNA synthesis is slightly different for each cell. We now provide single cell data (new Figure 1D) that shows that the timing of DNA synthesis is indeed different between individual cells, but that the average cell behavior is similar to the population data. The approach and the results are described in the results section on page 7:

"Our population data shows a continuous increase of DNA content, starting at 24 h after stimulation with 40 ng/ml HGF. To investigate if the shape of this curve is caused by temporal variability of the initiation of DNA synthesis in each cell, we analyzed the cell cycle progression in individual hepatocytes in the same experimental setting. We isolated primary mouse hepatocytes from mice transgenic for the Fucci2 cell cycle sensors established by Abe et al. (2013) (Fucci2 hepatocytes). These mice express mCherry-hCdt1 (amino acids 30 to 120) and mVenus-hGem (amino acids 1 to 110) under control of the Rosa26 promoter to distinguish cell cycle phases at the single cell level, but are otherwise of the same genetic background as the wild type mice used for our population studies (C57BL/6N). Additionally, we transduced the Fucci2 hepatocytes with adeno-associated viral vectors encoding Histone2B-mCerulean to facilitate single cell tracking. We performed live cell microscopy of growth factor depleted Fucci2 hepatocytes stimulated with 40 ng/ml HGF or left untreated (sampling rate of 15 min for up to 60 h) and manually tracked 20 cells (Supplementary Figure S1A). This data set allowed us to define the G1 (red), the G1/S (orange), the S/G2/M (green) and the early G1 (grey) phases of the cell cycle in a time-dependent manner. Fucci2 hepatocytes were in G1 phase after isolation. Without stimulation, hepatocytes remained in G1 throughout the observation period. A few cells entered the G1/S phase, but returned to G1 phase, indicating that the G1/S phase defined by the Fucci2 reported does not necessarily lead to DNA replication. Stimulation with 40 ng/ml HGF induced most of the monitored hepatocytes to undergo G1/S transition. These cells entered S/G2/M phase, executed DNA replication and performed mitosis and cytokinesis, as observed in the transmitted light channel. Immediately following mitosis, cells were in early G1 phase (Fig 1C). To link these single cell results with our population data, we quantified time-dependent G1/S transition (entry into the S/G2/M phase) events in these cells. The cumulative number of G1/S transition events versus time (Fig 1D) is consistent with the measured increase of the DNA

content of the entire population during the observation period. This congruent behavior indicates a similar HGF-dependent proliferation of the hepatocyte population and the averaged Fucci2 hepatocytes. We observed that while most of the cells respond to 40 ng/ml HGF during the observation period, the timing of transition to S/G2/M varied between the individual cells. Therefore, we expected that analysis of the G1/S transition on the population level would reveal a sigmoidal response, rather than a step-like increase."

> *- A very relevant recent study using a single-cell reporter of CDK2 (PMID: 24075009) has been overlooked. This study shows that the initial induction of CDK2 activity follows a multi-phasic trajectory with cells entering S-phase at the end of the first stage of CDK2 activity (which begins at different times in each cell). Given that similar complexity likely underlies the proliferation decision in this system, it is not clear what can be resolved with an immunoblot approach.*

We thank the reviewer for this suggestion. Our experiments with Fucci2 primary mouse hepatocytes indicate that single hepatocytes undergo G1/S transition at different times and show that the population average can be explained by the averaged individual cell behaviors. We furthermore discuss the coincidence of sustained CDK2 T160 phosphorylation and multiple cell cycles rounds in HGF-stimulated hepatocytes. We have now discussed this study in light of our new single cell results in the discussion section on page 21:

"Recently, a bifurcation point at the end of mitosis was detected by a live-cell sensor for CDK2 activity in constantly cycling cells (Spencer et al, 2013). In this report, it was discovered that a large fraction of MCF10A mammalian epithelial cells retains CDK2 activity and immediately commits to the next cell cycle. Additionally, CDK2 activity was shown to act as a threshold for the quiescence or proliferation decision. Interestingly, our single cell results in primary mouse hepatocytes are in line with these observations. We observed a linear correlation between CDK2 phosphorylation at T160 and proliferating hepatocytes for different concentrations of HGF, suggesting that CDK2 phosphorylation acts as a threshold in primary mouse hepatocytes. Additionally, we detected sustained high levels of pCDK2 T160 by quantitative immunoblotting (Fig 4B) in the presence of 40 ng/ml HGF. In line with the findings of Spencer et al. (2013), these sustained levels correlate with an immediate second round of replication for most of the cycling cells, as shown by single cell experiments (Fig 1C)."

> *- The model appears to treat DNA synthesis as a continuously variable process, which is appropriate if the model is meant to represent the population of cells. But the argument of the paper is about a discrete event - entry into S-phase vs. maintenance of G1- that occurs at the single cell level, and the relationship between these two views is never made clear.*

We now show the relationship between the single cell level and the cell population level. Two hypotheses could explain the impact of HGF on primary mouse hepatocytes: (i) HGF induces more cells to undergo G1/S transition, mediated by phosphorylation of CDK2, or (ii) HGF accelerates the kinetics of G1/S transition. As an example, we compared the effect of a low dose of HGF (4 ng/ml) to saturating HGF concentrations (100

ng/ml). Interestingly, we detect that more cells undergo G1/S transition with high HGF concentrations, while the restriction point remains unaffected. We describe these experiments in the results section on page 18:

"To quantitatively link G1/S transition components to cell cycle entry, we analyzed the impact of different doses of HGF on hepatocyte proliferation at the single Fucci2 hepatocyte level. In principle, increasing concentrations of HGF could either induce proliferation at earlier times or increase the number of responding cells. To answer this question, we exemplarily selected 4 ng/ml HGF, a stimulus that induces moderate levels of DNA synthesis (Fig 4K), and 100 ng/ml, a saturating concentration. We performed live cell imaging of Fucci2 primary mouse hepatocytes stimulated with these HGF concentrations and manually tracked 20 cells (Supplementary Figure S14A). We exemplarily show the G1, G1/S, S/G2/M and early G1 phases of the cell cycle in a time-dependent manner for these 20 hepatocytes in Figure 8A. Interestingly, displaying the number of cells that underwent G1/S transition at least once in a time-resolved manner for these two HGF doses (Fig 8B) indicated that an increase in HGF concentration does not result in an accelerated entry into S phase. Rather, the higher HGF concentration caused an increase in the number of responding cells."

Additionally, as described above, we now show the quantitative relationship between single cell and population behavior in Figure 1.

> *In short, population-level effects (monitored by immunoblot, generally very graded) and cellular-level behavior (presumably discrete, but with cell-to-cell variability) have not been carefully distinguished, which makes for a confusing and less than convincing case for the paper's major conclusion.*

We now combine our cell population studies with single cell analysis and thereby establish a bridge from population-level effects to cellular-level behavior. Interestingly, our single cell studies show that increasing HGF concentrations do not change the time to response in individual hepatocytes but rather primarily increase the number of proliferating hepatocytes. Furthermore, our studies establish a linear relation between HGF-dependent CDK2 T160 phosphorylation and HGF-induced hepatocyte cell cycle progression supporting our original conclusion of the gatekeeper function of CDK2 phosphorylation on T160 for hepatocyte proliferation.

3rd Editorial Decision 03 February 2015

Thank you again for submitting your work to Molecular Systems Biology. We have now heard back from the referee (previous referee #2) who was asked to evaluate your manuscript. As you will see below, the referee is satisfied with the modifications made and thinks that the study is now suitable for publication.

Before formally accepting your manuscript we would like to ask you to address the following points:

- We would like to ask you to provide the relevant information for accessing the model using the Biomodels database.

- We would also like to encourage you to include the source data for the figures (or figure panels) that display essential quantitative data.

Thank you for submitting this paper to Molecular Systems Biology.

Reviewer #1:

The authors have very carefully and adequately addressed the points raised.

1st Revision - authors' response 12 February 2015

Thank you very much for your positive evaluation of our manuscript.

As requested, we provide the following items:

ï The relevant information for accessing the model using the Biomodels database is now available on the Materials and Methods section of the manuscript (MODEL1502090000).

ï Source data for figure panels 1B, 1E, 2A-D, 2E-G, 2H, 2I-J, 2K,
2L-N, 2O, 4A-I, 4J, 4K, 4L, 4M, 7G, 7H, 7I, 7J, 7K, 8D and 8E are provided.
